# Genes Co-Expressed with *ESR2* Influence Clinical Outcomes in Cancer Patients: TCGA Data Analysis

**DOI:** 10.3390/ijms25168707

**Published:** 2024-08-09

**Authors:** Julia Maria Lipowicz, Agnieszka Malińska, Michał Nowicki, Agnieszka Anna Rawłuszko-Wieczorek

**Affiliations:** 1 Department of Histology and Embryology, Doctoral School, Poznan University of Medical Sciences, Święcickiego 6 Street, 60-781 Poznań, Poland; julia.lipowicz@student.ump.edu.pl; 2 Department of Histology and Embryology, Poznan University of Medical Sciences, Święcickiego 6 Street, 60-781 Poznań, Poland

**Keywords:** cancer, ESR2, bioinformatic, gene expression, transcriptomic, survival

## Abstract

ERβ has been assigned a tumor suppressor role in many cancer types. However, as conflicting findings emerge, ERβ’s tissue-specific expression and functional role have remained elusive. There remains a notable gap in compact and comprehensive analyses of *ESR2* mRNA expression levels across diverse tumor types coupled with an exploration of its potential gene network. In this study, we aim to address these gaps by presenting a comprehensive analysis of *ESR2* transcriptomic data. We distinguished cancer types with significant changes in *ESR2* expression levels compared to corresponding healthy tissue and concluded that *ESR2* influences patient survival. Gene Set Enrichment Analysis (GSEA) distinguished molecular pathways affected by *ESR2*, including oxidative phosphorylation and epithelial–mesenchymal transition. Finally, we investigated genes displaying similar expression patterns as *ESR2* in tumor tissues, identifying potential co-expressed genes that may exert a synergistic effect on clinical outcomes, with significant results, including the expression of *ACIN1*, *SYNE2*, *TNFRSF13C*, and *MDM4*. Collectively, our results highlight the significant influence of *ESR2* mRNA expression on the transcriptomic landscape and the overall metabolism of cancerous cells across various tumor types.

## 1. Introduction

Estrogen receptor β (ERβ) is one of the three predominant 17β-estradiol (E2) receptors in cells, along with estrogen receptor α (ERα) and G-protein-coupled estrogen receptor 1 (GPER1) [1]. ERβ and ERα are members of the nuclear receptor superfamily, while GPER1 is present on cell membranes [2,3]. ERβ is encoded by the gene *ESR2*, located on chromosome 14, containing eight exons. As *ESR2* is susceptible to alternative splicing, it can be transcribed into six isoforms, with ERβ1 characterized as the wild-type, full-length (530-amino-acids long, 59 kDa) protein [3,4,5]. For a full review and comparison of different ERs and their structure, we recommend perusing studies by Božović et al. [3] and Jia et al. [1].

ERβ displays diverse functions through genomic (nuclear, otherwise called classical) and non-genomic (rapid, extranuclear) pathways [6]. The classical pathways are initiated by the ligand-bound ERβ-E2 complex. This complex translocates into the nucleus, where it orchestrates the transcriptional regulation of target genes. The recognition of target genes can be mediated through the estrogen response element (ERE) canonical binding site (5′-GGTCAnnnTGACC-3′), a genomic target for ERβ, or via interaction with other transcription factors, including AP-1 and Sp-1 [6,7]. On the other hand, non-genomic pathways are carried out through rapid cellular mechanisms, including the protein–kinase signaling cascade or the cross-activation of growth factor signaling, possibly even in the absence of ERβ ligands [2,6,8].

ERβ is primarily expressed in the colon, kidney, lung, male reproductive tissues, and central nervous system [1]. While its precise physiological role remains incompletely understood, studies have suggested its role in immune response, the cardiovascular system, and prostate function [8,9]. In cancer, ERβ has been assigned a tumor suppressor role in many tumors, although exceptions exist, notably in lung cancer [6,8,10,11]. Nonetheless, the literature presents numerous inconsistencies. In breast cancer, ERβ expression levels were almost entirely lost compared to healthy tissue [12], and its re-expression inhibited breast cancer cell proliferation and upregulated apoptosis [13]. However, conflicting findings suggest that ERβ may enhance cell proliferation in breast cancer, with its expression correlating with poorer prognosis in some cases [14,15,16]. In colon cancer, most authors pointed to the protective role of ERβ. Animal models demonstrated that ERβ knock-down in mice leads to an increased number and size of intestinal adenomas, whereas treatment with ERβ-selective agonists presented the opposite effect [17,18]. Prostate cancer research further underscores the complexities surrounding ERβ. Multiple studies have identified it as a tumor suppressor or oncogene [19,20].

As described above, since ERβ discovery in 1996 [21], its tissue-specific expression and functional role have remained contentious due to conflicting findings across various studies. Andersson et al. [22] and Nelson et al. [23] investigated one of the reasons behind the inconsistencies in research, namely, antibody specificity. Their analysis revealed that only a fraction of commercially available antibodies are suitable for ERβ-targeted experiments, with PPZ0506 being the most specific [22,23]. Therefore, the validity of numerous publications may be compromised by the use of non-specific antibodies, particularly in studies based solely on proteomic data. Discrepancies between transcriptomic and proteomic research further underscore this challenge [7,22,23], likely partly due to non-specific protein detection. Further complications come from isoforms of ERβ, which diverge only through the domain encoded by the eighth exon [3,4,5]. Additionally, ERβ’s ability to localize within various cellular compartments, driven by both genomic and non-genomic activities [6], adds another layer of complexity [24,25].

In the literature, while there are studies that provide insight into *ESR2*/ERβ expression profiles across different tissues and cancer types, there remains a notable gap in compact and comprehensive analyses of *ESR2* mRNA expression levels across diverse tumor types coupled with an exploration of its potential functional implications. Furthermore, as a transcription factor, ERβ plays a considerable role in regulating cellular metabolism through different target genes, of which not all have been fully elucidated.

In this study, we aim to address these gaps by presenting a comprehensive analysis of *ESR2* transcriptomic data. We performed a series of bioinformatic analyses to specify ERβ’s role in various tumor types classified by The Cancer Genome Atlas (TCGA). We examined *ESR2* expression levels in tumors and corresponding healthy tissues to identify tumors potentially influenced by ERβ. Moreover, we carried out correlations between *ESR2* expression and tumor stage and grade. Furthermore, in selected tumor types, we extended our analysis to investigate overall survival (OS) and disease-free survival (DFS) in the context of *ESR2* expression. We also employed bioinformatic tools like Gene Set Enrichment Analysis (GSEA, software version 4.3.3.) to distinguish molecular pathways affected by this receptor. Finally, we investigated genes displaying similar expression patterns to ESR2 in tumor tissues, identifying potential co-expressed genes that may synergistically affect OS and DFS outcomes. Collectively, our results highlight the significant influence of *ESR2* mRNA expression on the transcriptomic landscape and overall metabolism of cancerous cells across various tumor types.

## 2. Results

### 2.1. Cancer Tissue and Corresponding Healthy Tissue Present Different ESR2 Expression Levels in Selected Cancer Types

**Figure 1:**
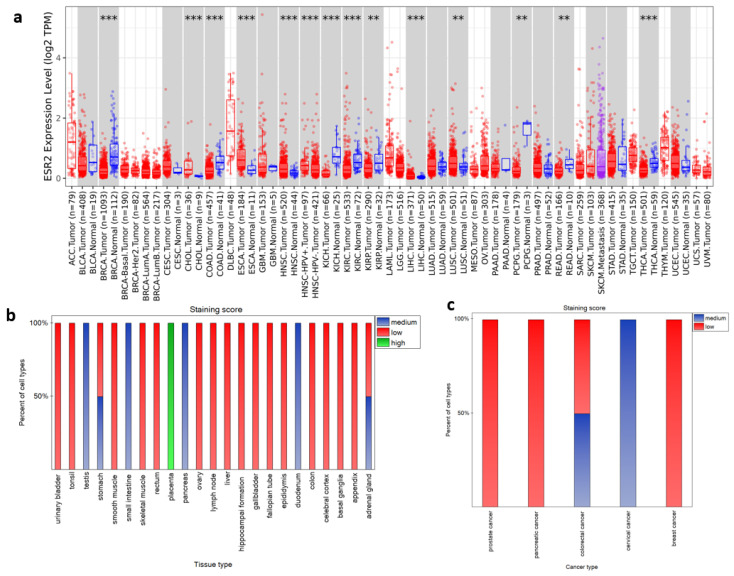
Transcriptomic and proteomic analysis of *ESR2* expression across tissues and cancer types. (**a**) *ESR2* expression levels across all TCGA tumor types and corresponding normal tissue (based on TIMER2.0 calculations). ** *p*-value < 0.01; *** *p*-value < 0.001. (**b**) ERβ protein staining scores in different tissue types (based on HPA data, CAB079300 antibody). (**c**) ERβ protein staining scores in pathological tissue types (based on HPA data, CAB079300 antibody). Abb.: adrenocortical carcinoma (ACC), bladder urothelial carcinoma (BLCA), breast invasive carcinoma (BRCA), cervical squamous cell carcinoma and endocervical adenocarcinoma (CESC), cholangio carcinoma (CESC), colon adenocarcinoma (COAD), lymphoid neoplasm diffuse large B-cell lymphoma (DLBC), esophageal carcinoma (ESCA), glioblastoma multiforme (GBM), head and neck squamous cell carcinoma (HNSC), kidney chromophobe (KICH), kidney renal clear cell carcinoma (KIRC), kidney renal papillary cell carcinoma (KIRP), acute myeloid leukemia (LAML), brain lower-grade glioma (LGG), liver hepatocellular carcinoma (LIHC), lung adenocarcinoma (LUAD), lung squamous cell carcinoma (LUSC), mesothelioma (MESO), ovarian serous cystadenocarcinoma (OV), pancreatic adenocarcinoma (PAAD), pheochromocytoma and paraganglioma (PCPG), prostate adenocarcinoma (PRAD), rectum adenocarcinoma (READ), sarcoma (SARC), skin cutaneous melanoma (SKCM), stomach adenocarcinoma (STAD), testicular germ cell tumor (TGCT), thyroid carcinoma (THCA), thymoma (THYM), uterine corpus endometrial carcinoma (UCEC), uterine carcinosarcoma (UCS), uveal melanoma (UVM).

To establish whether *ESR2* expression varies between cancerous and normal tissue, we analyzed transcriptomic data from the TIMER2.0 database. Figure 1a illustrates the differential expression of *ESR2* across selected tumor types compared to their corresponding normal tissue (tumor name abbreviations can be found in Abbreviations). Analysis of 22 tumor types based on TIMER2.0 data revealed 14 TCGA tumor types with significant differences (*p*-value < 0.05) with regard to *ESR2* expression between tumor and corresponding normal tissue (Figure 1a). Of these tumor types, eight exhibited higher mRNA expression levels in normal tissue, including breast invasive carcinoma (BRCA) (*p*-value < 0.001), colon adenocarcinoma (COAD) (*p*-value < 0.001), and kidney chromophobe (KICH) (*p*-value < 0.001) (Figure 1a). Conversely, five tumor types displayed higher expression in cancerous tissue, including cholangio carcinoma (CHOL) (*p*-value < 0.001) and head and neck squamous cell carcinoma (HNSC) (*p*-value < 0.001) (Figure 1a). Additionally, a significant difference was found between human papilloma virus (HPV)-positive and HPV-negative HNSC samples (*p*-value < 0.001), with the former presenting higher *ESR2* expression levels (Figure 1a).

To further describe our gene of interest, we explored the Human Protein Atlas database. Proteomic data from HPA’s immunostaining (antibody CAB079300) (Figure 1b,c) included 49 normal tissue samples, with detailed information regarding cell types. Data for 9 tissue types were not available and 17 tissue types did not exhibit positive staining in any cell type, including those from breast, endometrium, kidney, and lung (summary of tissue samples presented in HPA ENSG00000140009-ESR2/tissue). Still, in available proteomic data, we found high staining scores for the placenta and medium staining scores for the duodenum, pancreas, small intestine, and testis (Figure 1b). Adrenal glands and stomach tissue presented medium and low staining scores, depending on cell types, as shown in Figure 1b. For specific cell types in different tissue samples and their staining scores, we present Supplementary Figure S1. Furthermore, the HPA database provides immunostaining data on pathological tissues (summary of pathological tissue samples presented in HPA ENSG00000140009-ESR2/pathology). Out of 19 cancerous tissue samples, 14 had negative ERβ antibody staining, including lung cancer, melanoma, renal cancer, and testis cancer (Figure 1c). Furthermore, cervical cancer was classified as a medium staining score, whereas colorectal cancer had varying degrees of staining, ranging from low to medium across different samples (Figure 1c).

Unfortunately, samples used in Human Protein Atlas proteomic data for immunochemistry do not correspond to samples in the TCGA datasets. RNA-seq data from pathological tissues available in the Human Protein Atlas are likewise harnessed from the TCGA repository. Therefore, they do not correspond to proteomic data. Human Protein Atlas does provide HPA RNA-seq results regarding normal tissue samples. However, the number of samples per tissue is not sufficient to calculate the correlation. Therefore, further analyses were carried out based on available TCGA transcriptomic data.

### 2.2. ESR2 Expression Correlates with the Grade and Stage of Several Tumor Types

To determine whether *ESR2* expression level could be described as a potential biomarker in cancer, we searched the TISIDB database for correlations with the grade and stage of tumors.

**Figure 2:**
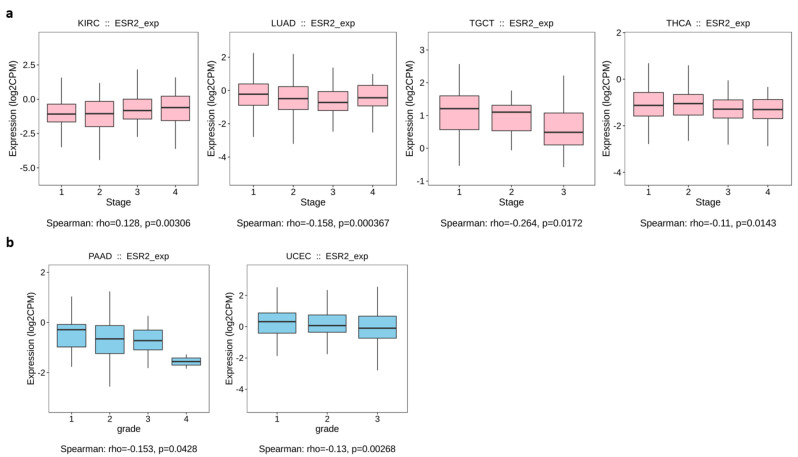
Correlation between *ESR2* expression and stage (**a**) or grade (**b**) in different cancer types. (**a**) Spearman’s correlation between *ESR2* expression level and tumor stage (based on TISIDB data). (**b**) Spearman’s correlation between *ESR2* expression level and tumor grade (based on TISIDB data). Abb.: kidney renal clear cell carcinoma (KIRC), lung adenocarcinoma (LUAD), testicular germ cell tumor (TGCT), thyroid carcinoma (THCA), pancreatic adenocarcinoma (PAAD), uterine corpus endometrial carcinoma (UCEC).

Out of 24 cancer types for which data were available on TISIDB, only 4 yielded significant results (*p*-value < 0.05) for the correlation between *ESR2* expression and tumor stage (Figure 2). We found a positive correlation for kidney renal clear cell carcinoma (KIRC) (*p*-value = 0.00306), in which a higher *ESR2* expression level correlated with advanced tumor stage, as shown in Figure 2a. A negative correlation was found for lung adenocarcinoma (LUAD) (*p*-value = 0.000367), testicular germ cell tumor (TGCT) (*p*-value = 0.0172), and thyroid carcinoma (THCA) (*p*-value = 0.0143), showing lower *ESR2* expression levels correlated with tumor stage (Figure 2a). Furthermore, we observed a significant correlation between *ESR2* expression and tumor grade in two out of eight cancer types. Specifically, pancreatic adenocarcinoma (PAAD) (*p*-value = 0.0428) and uterine corpus endometrial carcinoma (UCEC) (*p*-value = 0.00268) presented negative correlations; hence, lower *ESR2* expression correlated with tumor grade, as shown in Figure 2b. The TISIDB database did not yield other significant results for correlations of *ESR2* expression with the grade or stage of tumors.

### 2.3. ESR2 Expression Level Affects OS and DFS in Various Cancer Types

**Figure 3:**
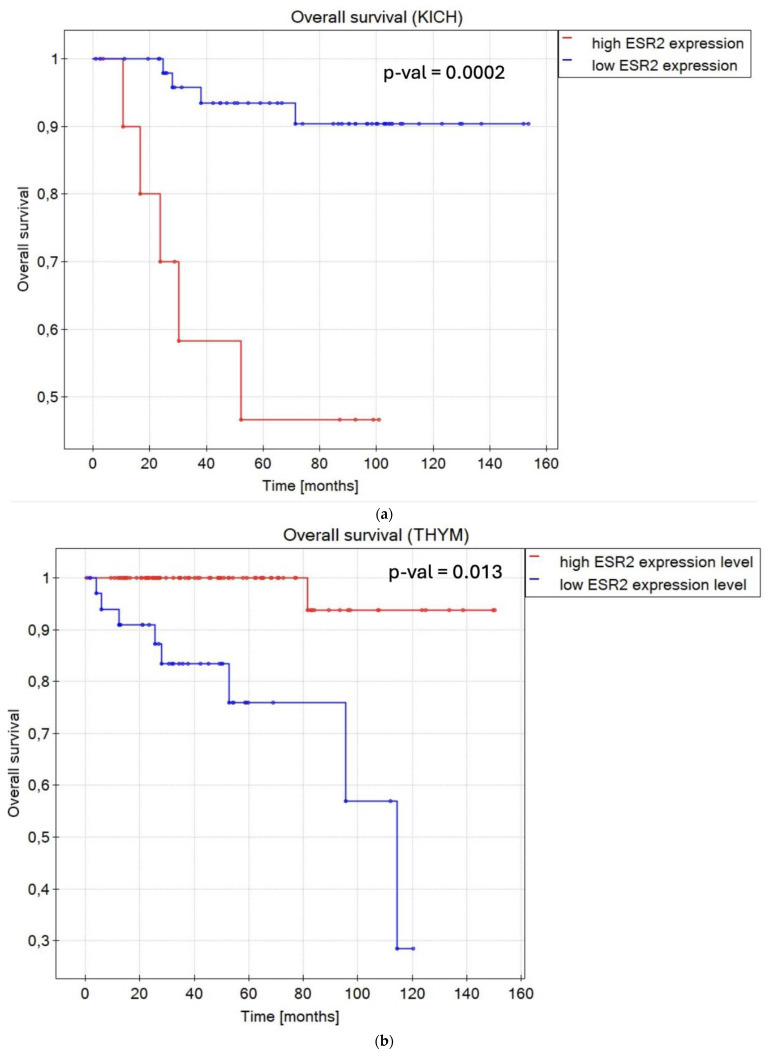

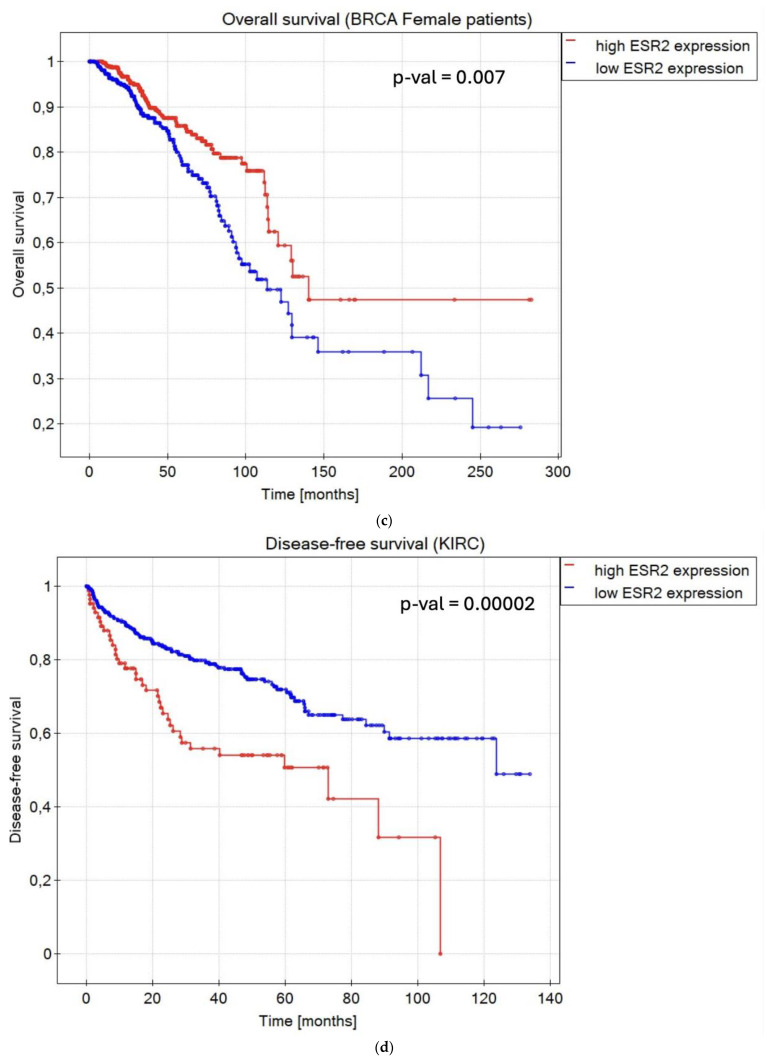

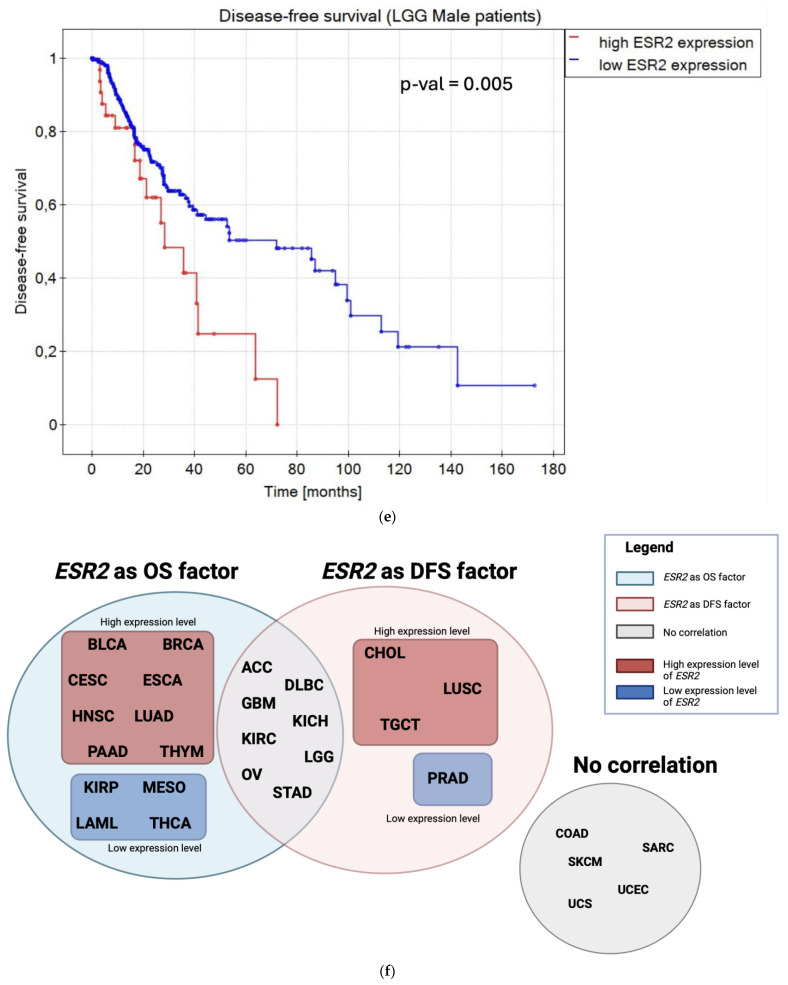
Kapplan–Meier plots of overall survival and disease-free survival, with *ESR2* expression level as a factor. (**a**) Kapplan–Meier plot of OS for KICH patients of both sexes. (**b**) Kapplan–Meier plot of OS for THYM patients of both sexes. (**c**) Kapplan–Meier plot of OS for BRCA female patients. (**d**) Kapplan–Meier plot of DFS for KIRC patients of both sexes. (**e**) Kapplan–Meier plot of DFS for LGG male patients. (**f**) Venn diagram showing *ESR2* expression level as a prognostic factor in terms of OS and DFS in TCGA tumor types. *p*-value < 0.05; FDR < 0.05. Abb.: kidney chromophobe (KICH), thymoma (THYM), breast invasive carcinoma (BRCA), kidney renal clear cell carcinoma (KIRC), brain lower grade glioma (LGG).

To review if *ESR2* may be considered a prognostic factor, we examined the influence of *ESR2* mRNA expression level on overall survival (OS) and disease-free survival (DFS) across various cancer types within the TCGA database (Figure 3). We noticed that *ESR2* presented both positive and negative prognostic capacity, in some cases with regard to patient sex. Tumors that presented a reliance on *ESR2* expression level in terms of OS and DFS were described in the literature as having a sex bias in occurrence and clinical outcomes [26,27,28,29,30,31]. To delineate patients’ subgroups based on *ESR2* expression levels, we employed the Evaluate Cutpoints R application algorithm [32], facilitating the classification of individuals into “high *ESR2* expression level” and “low *ESR2* expression level” groups. Subsequently, we conducted OS and DFS analyses based on this division. Interestingly, 20 out of 29 cancer types presented significant results (*p*-value < 0.05) when we analyzed *ESR2*’s influence on OS. Additionally, 12 cancer types yielded significant results with regard to *ESR2*’s influence on DFS. Representative data are shown (Figure 3); for every significant result plotted, we present Supplementary Figures S2 and S3.

In terms of OS, in renal cancers, KICH (*p*-value = 0.0002) (Figure 3a), and KIRC (*p*-value = 0.000001) (Figure S2), a low *ESR2* expression level was associated with longer OS compared to a high *ESR2* expression level for both sexes. Likewise, low *ESR2* expression was favorable in brain lower-grade glioma (LGG) (*p*-value = 0.000001) (Figure S2). In the case of patients with lymphoid neoplasm diffuse large B-cell lymphoma (DLBC) (*p*-value = 0.001) (Figure S2) and thymoma (THYM) (*p*-value = 0.013) (Figure 3b), a high *ESR2* expression level was a positive prognostic factor. Notably, sex was determined to be a significant factor with regard to OS in cases of adrenocortical carcinoma (ACC) (*p*-value = 0.012) (Figure S2), bladder urothelial carcinoma (BLCA) (*p*-value = 0.01) (Figure S2), BRCA (*p*-value = 0.007) (Figure 3c), esophageal carcinoma (ESCA) (*p*-value = 0.029) (Figure S2), HNSC (*p*-value = 0.005) (Figure S2), kidney renal papillary cell carcinoma (KIRP) (*p*-value = 0.043) (Figure S2), acute myeloid leukemia (LAML) (*p*-value = 0.021) (Figure S2), LUAD (*p*-value = 0.023) (Figure S2), PAAD (*p*-value = 0.03) (Figure S2), stomach adenocarcinoma (STAD) (*p*-value = 0.004) (Figure S2), and THCA (*p*-value = 0.045) (Figure S2). Female patients with high *ESR2* expression levels diagnosed with ACC, BLCA, BRCA, and ESCA presented longer OS compared to those with low *ESR2* expression levels. When it came to male patients, high *ESR2* expression levels in HNSC, LUAD, and PAAD and low *ESR2* expression levels in KIRP, LAML, mesothelioma (MESO), STAD, and THCA were attributed to longer OS. Patient groups with only one sex, cervical squamous cell carcinoma and endocervical adenocarcinoma (CESC) (*p*-value = 0.004) (Figure S2), and ovarian serous cystadenocarcinoma (OV) (*p*-value = 0.002) (Figure S2) (only female samples) presented high *ESR2* expression levels as a favorable factor with regard to OS.

Regarding DFS, *ESR2* expression level was considered a prognostic factor in 14 tumor types. In KICH (*p*-value < 0.000001) (Figure S3), KIRC (*p*-value = 0.00002) (Figure 3d), and glioblastoma multiforme (GBM) (*p*-value = 0.003) (Figure S3), a low *ESR2* expression level was associated with longer DFS, regardless of sex. Sex was determined as a significant factor in six tumor types considering DFS, not counting datasets with only one sex (TGCT and prostate adenocarcinoma (PRAD)). In ACC (*p*-value = 0.007) (Figure S3), female patients presented high *ESR2* expression levels as a favorable factor in terms of DFS. In male patients with DLBC (*p*-value = 0.03) (Figure S3), LGG (*p*-value = 0.005) (Figure 3e), and STAD (*p*-value = 0.003) (Figure S3), low *ESR2* expression was considered a favorable factor in terms of DFS. Similarly, a low *ESR2* expression level was considered a favorable prognostic factor in PRAD (only male samples) (*p*-value = 0.024) (Figure S3), while a high *ESR2* expression level was significantly correlated with longer DFS in TGCT (*p*-value = 0.004) (Figure S3) (only male samples).

However, no significant results were obtained in calculations of remaining TCGA tumor types regarding either overall survival or disease-free survival with *ESR2* expression level as a prognostic factor. Therefore, we categorized tumor types based on the prognostic role of *ESR2* expression (Figure 3f).

### 2.4. ESR2 Affects Various Molecular Pathways in Cancer

To better characterize *ESR2*’s role in various cancer types, we carried out Gene Set Enrichment Analysis (GSEA). We performed an analysis to compare *ESR2*-associated transcriptomic profiles with pre-defined lists of gene signatures. We selected human molecular signatures from different MSigDB collections, namely, Hallmark Gene Sets Collection and C2 Curated Gene Sets Collection. Across 30 cancer types, significant results (*p*-value < 0.05 and FDR < 0.05) were obtained for 18 types in the Hallmark collection analysis and 7 cancer types in the C2 collection.

**Figure 4:**
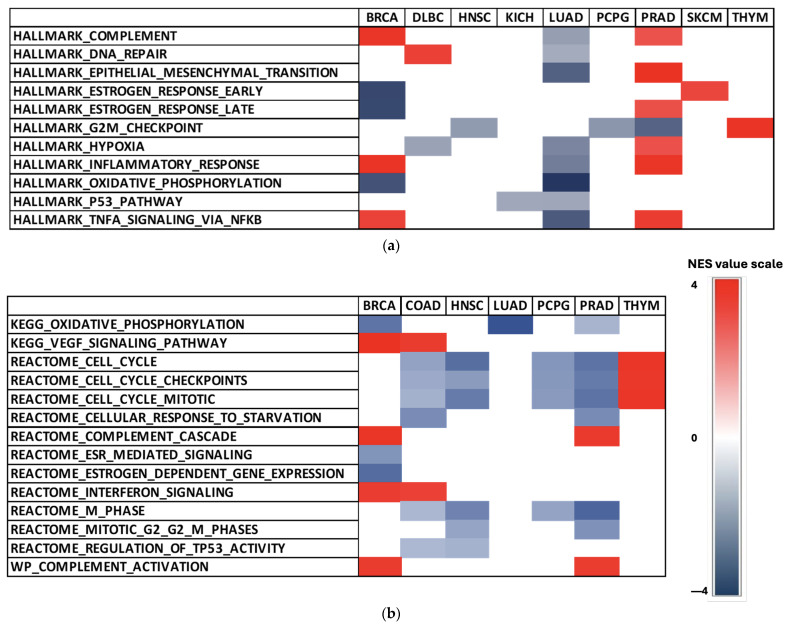
Heatmaps with NES values for selected gene sets in TCGA tumor types. (**a**) Results of Hallmark collection analysis presented as a heatmap. (**b**) Results of C2 collection analysis presented as a heatmap. *p*-value < 0.05; FDR < 0.05. Abb.: breast invasive carcinoma (BRCA), lymphoid neoplasm diffuse large B-cell lymphoma (DLBC), head and neck squamous cell carcinoma (HNSC), kidney chromophobe (KICH), lung adenocarcinoma (LUAD), pheochromocytoma and paraganglioma (PCPG), prostate adenocarcinoma (PRAD), skin cutaneous melanoma (SKCM), thymoma (THYM), colon adenocarcinoma (COAD).

Within Hallmark gene sets, *ESR2* influence was most abundant in BLCA, BRCA, LUAD, MESO, PRAD, and TGCT (Figure 4a). The most enriched sets in the Hallmark collection were the *G2M* checkpoint gene set, epithelial–mesenchymal transition gene set, hypoxia gene set, and *TNFα* signaling via *NFκ*β gene set (Figure 4a).

We found significant enrichment in the estrogen response gene set (Hallmark collection) in BRCA (FDR = 0.000), PRAD (FDR = 0.027), and skin cutaneous melanoma (SKCM) (FDR = 0.032) (Figure 4a). However, it is important to note that the estrogen response gene set included both ERα and ERβ. Therefore, those results should be interpreted with caution. Consistently negative NES values in 13 selected Hallmark gene sets were observed in BLCA, LUAD, MESO, and TGCT (Figure 4a). High NES values of Hallmark sets were achieved in PRAD (Figure 4a), with the epithelial–mesenchymal transition gene set demonstrating the highest score (FDR = 0.000).

We also found significant results for the C2 collection, particularly in gene sets associated with cell cycle and TP53 activity (Figure 4b). The estrogen-dependent gene expression and ESR-mediated signaling gene sets from the Reactome sub-collection in C2 yielded significant NES in BRCA (FDR = 0.000; FDR = 0.001) (Figure 4b). Additionally, low NES values were observed in LUAD (Figure 4b), with the oxidative phosphorylation gene set (KEGG sub-collection) reaching the lowest NES of the analysis (FDR = 0.000).

### 2.5. Various Genes Present Expression Patterns Similar to That of ESR2 in Cancer

As mentioned above, ERβ displays its functions through genomic and non-genomic pathways [6]. Signaling cascades controlled by ERβ include sets of known cofactors, including Akt, AP1, and SP1 [6,7]. However, the entire network of factors cooperating with ERβ is not known. ERβ has been shown to play a significant role in carcinogenesis, glucose homeostasis, and other important pathways [1]; thus, presenting which genes might be associated with this receptor would be valuable. Hence, we decided to look for genes that showed mRNA expression patterns similar to that of *ESR2* in TCGA cancer types.

Employing transcriptomic data from cBioPortal, we established lists of thousands of co-expressed genes for each tumor type. The top co-expressed genes, considering both positive and negative correlations, were selected using Spearman’s coefficient. Subsequently, we conducted a comprehensive search to identify genes that appeared most frequently in these lists, pinpointing potential targets. Our analyses were centered on elucidating the joint impact of *ESR2* and the selected co-expressed genes on overall survival (OS) and disease-free survival (DFS). From the initial pool of tens of thousands of genes, we designated 24 genes as the most promising targets, as listed in Table 1.

**Figure 5:**
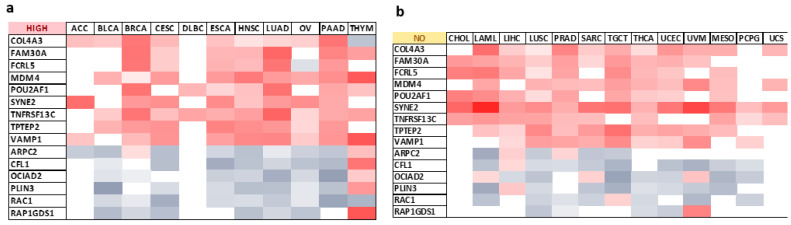

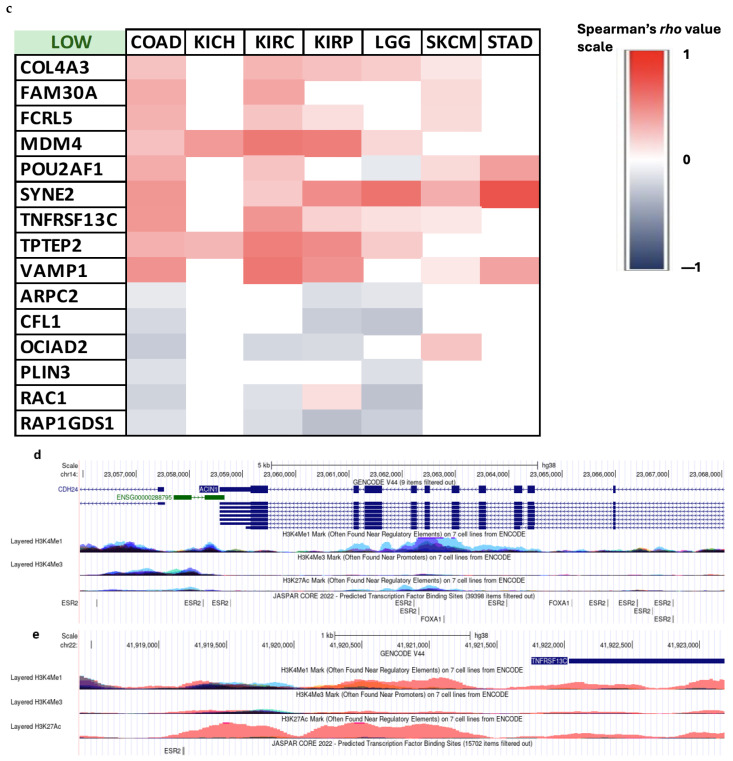
Genes presenting similar expression patterns as *ESR2*. (**a**) Heatmap of Spearman’s correlation score (*rho*) in tumors with high ESR2 expression levels as an extended OS or DFS marker. (**b**) Heatmap of Spearman’s correlation score (*rho*) in tumors with no correlation between *ESR2* expression and extended OS or DFS. (**c**) Heatmap of Spearman’s correlation score (*rho*) in tumors with low *ESR2* expression levels as an extended OS or DFS marker. (**d**) USCS Genome Browser screengrab showing epigenetic marks of open chromatin and ERβ (*ESR2*) and HNF3α (*FOXA1*) binding sites in the *ACIN1* sequence. (**e**) UCSC Genome Browser screengrab showing epigenetic marks of open chromatin and ERβ (*ESR2*) and HNF3α (*FOXA1*) binding sites in the TNFRSF13C sequence. *p*-value < 0.05; FDR < 0.05. Abb.: adrenocortical carcinoma (ACC), bladder urothelial carcinoma (BLCA), breast invasive carcinoma (BRCA), cervical squamous cell carcinoma and endocervical adenocarcinoma (CESC), lymphoid neoplasm diffuse large B-cell lymphoma (DLBC), esophageal carcinoma (ESCA), head and neck squamous cell carcinoma (HNSC), lung adenocarcinoma (LUAD), ovarian serous cystadenocarcinoma (OV), pancreatic adenocarcinoma (PAAD), thymoma (THYM), cholangio carcinoma (CHOL), acute myeloid leukemia (LAML), liver hepatocellular carcinoma (LIHC), lung squamous cell carcinoma (LUSC), prostate adenocarcinoma (PRAD), sarcoma (SARC), testicular germ cell tumor (TGCT), thyroid carcinoma (THCA), uterine corpus endometrial carcinoma (UCEC), uveal melanoma (UVM), mesothelioma (MESO), pheochromocytoma and paraganglioma (PCPG), uterine carcinosarcoma (UCS), colon adenocarcinoma (COAD), kidney chromophobe (KICH), kidney renal clear cell carcinoma (KIRC), kidney renal papillary cell carcinoma (KIRP), brain lower-grade glioma (LGG), skin cutaneous melanoma (SKCM), stomach adenocarcinoma (STAD).

Notably, our findings highlight seven genes (*FAM30A*, *MDM4*, *POU2AF1*, *SYNE2*, *TNFRSF13C*, *TPTEP2*, *VAMP1*) presenting positive correlations with *ESR2* expression patterns in all tumor types with high *ESR2* expression as a positive prognostic factor with regard to OS or DFS (Figure 5a). Conversely, one target gene, *RAC1*, showed a negative correlation with *ESR2* expression patterns in those tumor types.

Furthermore, eight target genes (*COL4A3*, *FAM30A*, *FCRL5*, *MDM4*, *SYNE2*, *TNFRSF13C*, *TPTEP2*, *VAMP1*) were systematically positively correlated with *ESR2* expression patterns in tumor types with low *ESR2* expression as a prognostic factor concerning OS or DFS (Figure 5c). Negative correlations across those tumor types were observed in four target genes (*ARPC2*, *CFL1*, *PLIN3*, *RAP1GDS1*) (Figure 5c). The expression of selected genes in tumors without a significant influence of *ESR2* on clinical outcomes is shown in Figure 5b.

To further examine the relationship between target genes and *ESR2*, we used UCSC Genome Browser. Within our pool of target genes, we looked for predicted transcription factor binding sites for ERβ (ERE canonical binding site 5′-GGTCAnnnTGACC-3′). As in computational modeling approaches, there is no clear difference between functional and non-functional transcription factor binding sites; we searched for EREs that co-occurred with epigenetic marks of active regulatory elements (H3K4me1 and H3K27ac) and promoters (H3K4me3). Furthermore, to strengthen the efficacy of our search, we added a predicted transcription factor binding site for HNF3α (*FOXA1*) (sequence 5′-[AC]A[AT]T[AG]TT[GT][AG][CT]T[CT]-3′) as a component of our research due to its known cooperation with ERβ in regulating gene activation [68]. Among the 24 target genes, 12 presented strong evidence of potential ERβ binding sites within their promoter or regulatory regions and were used as target genes in further analysis. Nine target genes (*ACIN1* (Figure 5d and Figure S4), *FNBP4* (Figure S4), *MDM4* (Figure S4), *NDUFB3* (Figure S4), *OCIAD2* (Figure S4), *PLIN3* (Figure S4), *POU2AF1* (Figure S4), *RAC1* (Figure S4), *SYNE2* (Figure S4)) displayed multiple ERβ and HNF3α binding sites within regions with open chromatin epigenetic marks. Three target genes (*CFL1* (Figure S4), *TMEM141* (Figure S4), *TNFRSF13C* (Figure 5e)) presented ERβ binding sites without HNF3α binding sites present, though ERβ binding sites co-occurred with H3K4Me1, H3K4Me3, and H3K27Ac marks.

The remaining 12 target genes failed to exhibit substantial evidence to qualify as direct binding targets of ERβ. *ARPC2* (Figure S5), *COL4A3* (Figure S5), *FCRL5* (Figure S5), *LENG8 (*Figure S5), *RAP1GDS1* (Figure S5), *VAMP1* (Figure S5), and *ZFYVE26* (Figure S5) displayed multiple ERβ and HNF3α binding sites, notwithstanding epigenetic marks of regulatory elements or promoters. Hence, those target genes were not accounted for as possible hits. *FAM30A* (Figure S5) and *PPP1R3E* (Figure S5) showed multiple ERβ binding sites without simultaneous open chromatin marks present. Subsequently, they were not considered a significant result. *TPTEP2* (Figure S5) and *ZBTB25* (Figure S5) showed multiple HNF3α binding sites without ERβ binding sites present; thus, these genes were not recognized as possibly significant results. *CELF6* (Figure S5) presented very little evidence of being regulated by ERβ due to scarce ERβ or HNF3α binding sites, all of which were not within open chromatin epigenetic marks; consequently, it was not analyzed further.

### 2.6. Selected Genes Present a Combined Effect with ESR2 on OS/DFS in Several Cancer Types

**Figure 6:**
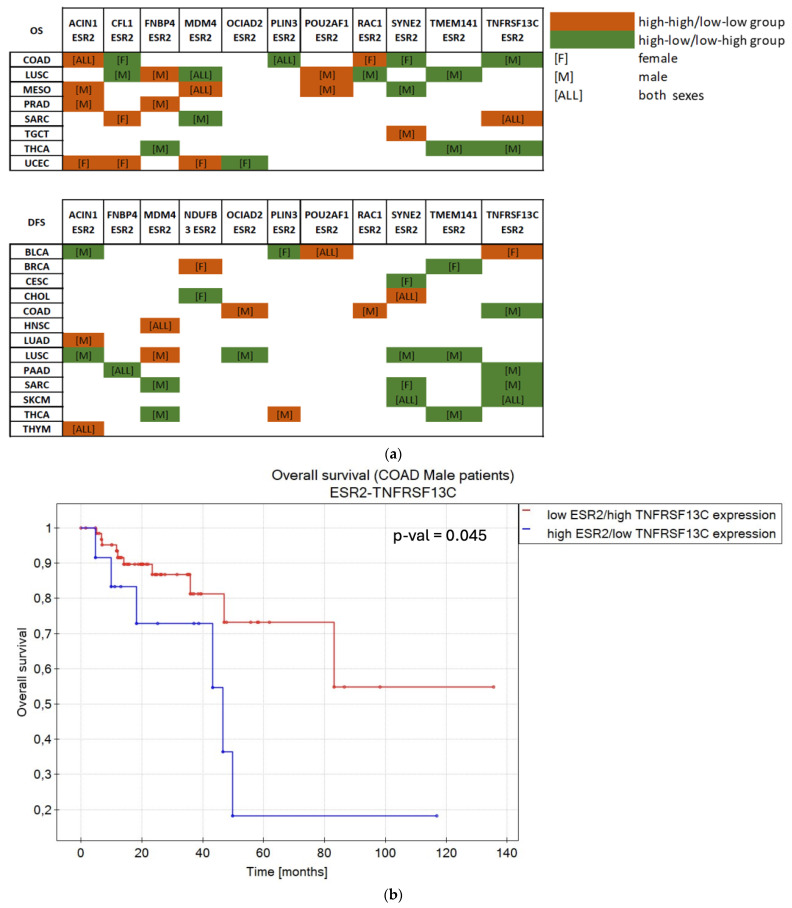

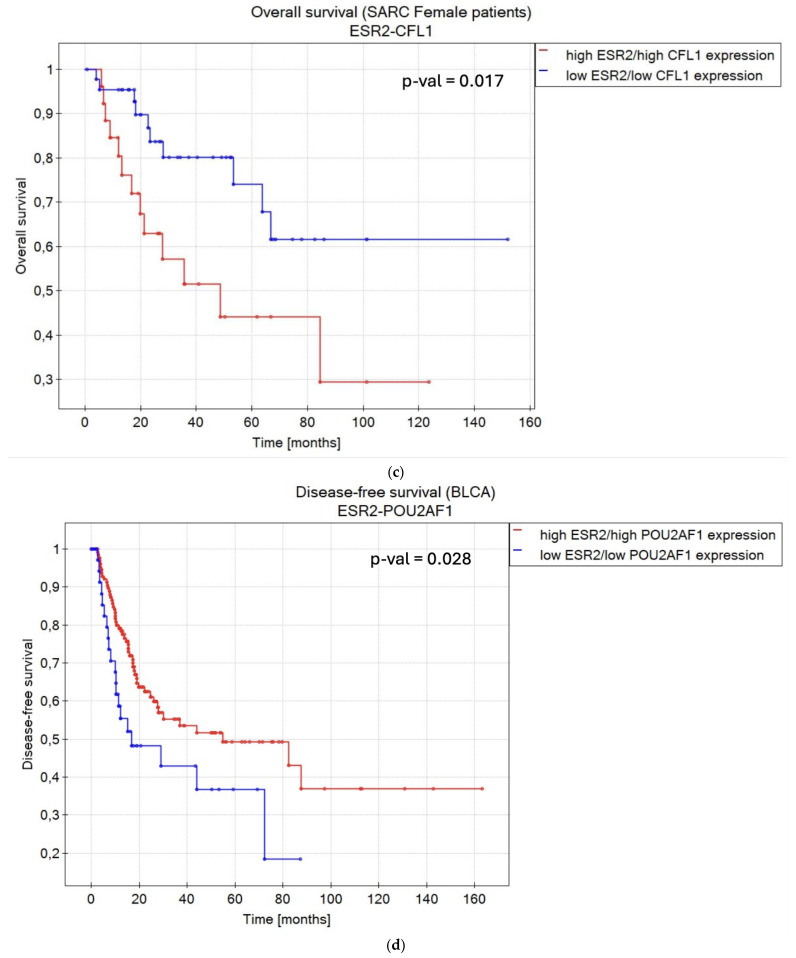
*ESR2* and selected target genes present a combined effect on OS and DFS. (**a**) Summary showing the combined effect of selected target genes and *ESR2* on OS and DFS with regard to sex. (**b**) Kapplan–Meier plot of male COAD patients with co-expression of *ESR2* and *TNFRSF13C* as a factor in terms of OS. (**c**) Kapplan–Meier plot of female SARC patients with co-expression of *ESR2* and *CFL1* as a factor in terms of OS. (**d**) Kapplan–Meier plot of BLCA patients of both sexes with co-expression of *ESR2* and *POU2AF1* as a factor in terms of DFS. *p*-value < 0.05; FDR < 0.05. Abb.: colon adenocarcinoma (COAD), lung squamous cell carcinoma (LUSC), mesothelioma (MESO), prostate adenocarcinoma (PRAD), sarcoma (SARC), testicular germ cell tumor (TGCT), thyroid carcinoma (THCA), uterine corpus endometrial carcinoma (UCEC), bladder urothelial carcinoma (BLCA), breast invasive carcinoma (BRCA), cervical squamous cell carcinoma and endocervical adenocarcinoma (CESC), cholangio carcinoma (CHOL), head and neck squamous cell carcinoma (HNSC), lung adenocarcinoma (LUAD), pancreatic adenocarcinoma (PAAD), skin cutaneous melanoma (SKCM), thymoma (THYM).

As shown in Figure 3f, *ESR2* was not considered a sole prognostic factor in 9 cancer types in terms of OS and 17 cancer types in terms of DFS. Hence, we searched for a combined impact of *ESR2* and 12 selected target genes on OS or DFS in those tumors. Before further analyzing the combined effects with ESR2, we examined the individual impact of target genes on the OS or DFS. Only genes that did not independently affect OS or DFS were considered (12 target genes: *ACIN1*, *CFL1*, *FNBP4*, *MDM4*, *NDUFB3*, *OCIAD2*, *PLIN3*, *POU2AF1*, *RAC1*, *SYNE2*, *TMEM141*, *TNFRSF13C*). Patients were divided into “high” and “low” expression groups (based simultaneously on *ESR2* and target gene expression, with cut-off points calculated separately for each gene), including sex as a factor. Significant correlations (*p*-value < 0.05) were found for 17 cancer types, as shown in Figure 6 and Supplementary Figures S6 and S7. Lung squamous cell carcinoma (LUSC) and COAD demonstrated the greatest number of correlations, yielding nine and seven significant outcomes between *ESR2* and target genes, respectively.

*ACIN1* and *SYNE2* showed a combined effect with *ESR2* on either OS or DFS in 9 out of 17 cancer types, *TNFRSF13C* showed a combined effect with *ESR2* in 8 cancer types, and *MDM4* showed a combined effect with *ESR2* in 7 cancer types. The remaining eight target genes (*CFL1*, *FNBP4*, *NDUFB3*, *OCIAD2*, *PLIN3*, *POU2AF1*, *RAC1*, *TMEM141*) presented a combined influence in five or fewer cancer types (Figure 6a).

BLCA patients of both sexes benefited from simultaneous high expression levels of *POU2AF1* and *ESR2* in terms of DFS (*p*-value = 0.028) (Figure 6d). Additionally, female patients with high levels of expression of *TNFRSF13C* and *ESR2* showed longer DFS (*p*-value = 0.017) (Figure S7). Notably, low levels of expression of *PLIN3* in female patients (*p*-value = 0.045) and high expression of *ACIN1* in male patients (*p*-value = 0.007) correlated with high and low levels of *ESR2* expression, respectively, resulting in extended DFS (Figure S7).

Moreover, BRCA female patients presented correlations regarding *NDUFB3* (*p*-value = 0.039) and *TMEM14* (*p*-value = 0.046) (Figure S7). Simultaneous high expression levels of *NDUFB3* and *ESR2* were associated with longer DFS, while low *TMEM141*–high *ESR2* expression dynamics prolonged DFS in BRCA female patients.

CESC data yielded one significant correlation. Female patients with low *SYNE2*–high *ESR2* expression benefited in terms of DFS (*p*-value = 0.008) (Figure S7).

Patients of both sexes with CHOL presented longer DFS when *SYNE2* and *ESR2* were simultaneously highly expressed (*p*-value = 0.049) (Figure S7). Within female CHOL patients, longer DFS was observed with *NDUFB3* low–*ESR2* high expression (*p*-value = 0.047) (Figure S7).

COAD data presented correlations between *ESR2* and seven selected genes. Both sexes exhibited longer OS in high–high expression dynamics between *ACIN1* and *ESR2* (*p*-value = 0.033) and low *PLIN3* and high *ESR2* expression (*p*-value = 0.001) (Figure S6). Female patients with low *ESR2*–high *CFL1* (*p*-value = 0.017) or *SYNE2* (*p*-value = 0.033) expression or high expression of *RAC1* and *ESR2* (*p*-value = 0.02) were characterized by longer OS (Figure S6), while male patients benefited from simultaneous high expression levels of *RAC1* and *ESR2* in terms of DFS (*p*-value = 0.007) (Figure S7). High *TNFRSF13C* and low *ESR2* expression positively affected OS (*p*-value = 0.045) (Figure 6b) and DFS (*p*-value < 0.000001) (Figure S7) in male patients. Moreover, male patients with COAD benefited from simultaneous low expression levels of *OCIAD2* and *ESR2* (*p*-value < 0.000001) (Figure S7).

HNSC data yielded one significant correlation, namely, high *MDM4* and high *ESR2* expression levels contributed to longer DFS in both female and male patients (*p*-value = 0.033) (Figure S7).

Male LUAD patients showed longer DFS when *ACIN1* and *ESR2* presented high expression levels (*p*-value = 0.013) (Figure S7).

LUSC data yielded the greatest number of correlations between *ESR2* and target genes. The OS of patients of both sexes was prolonged with high *MDM4* and low *ESR2* expression (*p*-value = 0.036) (Figure S6). Simultaneously, high *RAC1* and low *ESR2* expression contributed to longer OS in LUSC patients of both sexes (*p*-value = 0.015) (Figure S6). Male LUSC patients showed longer DFS with high *ESR2* and low *ACIN1* (*p*-value = 0.009), *OCIAD2* (*p*-value = 0.022), and *SYNE2* (*p*-value = 0.007) or low ESR2 and high *TMEM141* expression (*p*-value = 0.032) (Figure S7). DFS of male patients was extended with concurrent high expression levels of both *MDM4* and *ESR2* (*p*-value = 0.005) (Figure S7), while longer OS was also observed for high expression levels of *ESR2* and *FNBP4* (*p*-value = 0.036) or *POU2AF1* (*p*-value = 0.013) (Figure S6). The high–low expression dynamic between *ESR2* and *CFL1* (*p*-value = 0.02) or *TMEM141* (*p*-value = 0.046) and the low–high expression dynamic between *ESR2* and *RAC1* (*p*-value = 0.015) presented longer OS in LUSC male patients as well (Figure S6).

OS of MESO patients was affected by correlations between *ESR2* and *ACIN1*, *MDM4*, *POU2AF1*, or *SYNE2* expression levels. Simultaneous high expression levels of *ESR2* and *MDM4* correlated with longer OS in patients of both sexes (*p*-value = 0.037), while low levels of *ACIN1* (*p*-value = 0.001) or *POU2AF1* (*p*-value = 0.004) expression combined with high and low levels of *ESR2* expression, respectively, correlated with longer OS of male patients (Figure S6). Male MESO patients benefited from longer OS with high *SYNE2* and low *ESR2* expression levels (*p*-value = 0.028) (Figure S6).

PAAD data presented significant prognostic outcomes for two target genes, *FNBP4* and *TNFRSF13C*. Low *FNBP4* and high *ESR2* expression correlated with longer DFS in patients of both sexes (*p*-value = 0.013) (Figure S7). Concurrently, low *TNFRSF13C* and high *ESR2* correlated with longer DFS in male patients with PAAD (*p*-value = 0.016) (Figure S7).

Male patients with PRAD showed longer OS when a low level of *ESR2* expression was correlated with low expression levels of either *ACIN1* (*p*-value = 0.048) or *FNBP4* (*p*-value = 0.03) (Figure S6).

Sarcoma (SARC) patients with simultaneous low expression levels of *TNFRSF13C* and *ESR2* presented longer OS (*p*-value = 0.034) (Figure S6). Similarly, female SARC patients with low expression levels of *CFL1* and *ESR2* showed longer OS (*p*-value = 0.017) (Figure 6c). *TNFRSF13C* and *ESR2* high–low expression dynamics correlated with longer DFS in male *SARC* patients (*p*-value = 0.017) (Figure S7). Sex was also a factor in correlations of *ESR2* with *MDM4* and *SYNE2*. Male patients with SARC showed longer DFS (*p*-value = 0.044) (Figure S7) and OS (*p*-value = 0.015) (Figure S6) with high *MDM4* and low *ESR2* expression levels. Further, female patients with SARC with low *ESR2* and high *SYNE2* expression presented longer DFS (*p*-value = 0.008) (Figure S7).

In SKCM data, two noteworthy correlations were identified. Patients with low *ESR2* and high *SYNE2* (*p*-value = 0.033) or *TNFRSF13C* (*p*-value = 0.046) expression exhibited extended DFS, irrespective of sex (Figure S7).

A significant correlation emerged from the TGCT data as well. Simultaneous high expression levels of *ESR2* and *SYNE2* were associated with extended OS in male patients (*p*-value = 0.013) (Figure S6).

In male patients with THCA, notable correlations were observed between *ESR2* and four target genes. Specifically, *FNBP4* demonstrated an impact on both DFS (*p*-value = 0.028) (Figure S7) and OS (*p*-value = 0.00007) (Figure S6) when the expression levels of *FNBP4* and *ESR2* exhibited a high–low dynamic correlation. A similar relationship between high *TMEM141* and low *ESR2* influenced both DFS (*p*-value = 0.011) (Figure S7) and OS (*p*-value = 0.001) (Figure S6) in male patients with THCA. Moreover, male patients with concurrent low expression levels of *PLIN3* and *ESR2* exhibited extended DFS (*p*-value = 0.039) (Figure S7). Additionally, high *TNFRSF13C* and concomitant low *ESR2* expression correlated with prolonged OS in male patients (*p*-value = 0.00005) (Figure S6).

In the THYM data, one significant result emerged, revealing that a simultaneous high expression level between *ACIN1* and *ESR2* was correlated with extended DFS in both sexes (*p*-value = 0.032) (Figure S7).

Within the UCEC data, correlations between *ESR2* and four target genes were identified. Female UCEC patients who exhibited simultaneous low expression levels of *ESR2* and *ACIN1* (*p*-value = 0.018), *CFL1* (*p*-value = 0.046), or *MDM4* (*p*-value = 0.04) demonstrated prolonged OS (Figure S6). Furthermore, female patients with low *ESR2* and high *OCIAD2* expression also exhibited extended OS (*p*-value = 0.015) (Figure S6).

Analyses of ACC, DLBC, LAML, pheochromocytoma and paraganglioma (PCPG), uterine carcinosarcoma (UCS), and uveal melanoma (UVM) did not yield significant results.

## 3. Discussion

This study presents a comprehensive bioinformatics analysis of the potential role of *ESR2* across various TCGA tumor types. We used tools and databases containing transcriptomic data to analyze whether *ESR2* mRNA expression levels significantly influence overall survival and disease-free survival. Furthermore, we found genes co-expressed with *ESR2* and evaluated their combined impact on patient survival.

Our analysis displayed that *ESR2* mRNA expression was significantly elevated in normal tissue corresponding to eight tumor types, namely, BRCA, COAD, KICH, KIRC, KIRP, and THCA, aligning with the literature [69,70,71,72]. Furthermore, *ESR2* mRNA expression was significantly higher in tumor tissue in CHOL, ESCA, HNSC, LIHC, and LUSC, which is consistent with prior research findings [73,74,75,76,77]. Next, we investigated a correlation between tumor grade or stage and *ESR2* mRNA expression using the TISIDB database. Detected in our analysis, a negative correlation between ESR2 expression and grade in UCEC aligns with the literature [78,79]. Conversely, a negative correlation in PAAD contradicts previous findings, which notably relied on ERβ staining scores instead of transcriptomic data [80]. Moreover, ESR2 expression correlated with tumor stage in our study in KIRC, LUAD, TGCT, and THCA. The positive correlation found in KIRC and negative correlation found in LUAD and TGCT are consistent with the majority of previous research. However, some teams reported contradictory results. We attribute these discrepancies to the fact that analyses were conducted using immunohistochemical methods and various antibodies [81,82,83,84,85]. For instance, previous research by Dong et al. showed no association of ERβ expression with tumor stage in THCA, although they examined the protein expression of only isoform 2 of ERβ [86], not *ESR2* mRNA levels.

We analyzed whether *ESR2* mRNA expression influenced overall survival and disease-free survival across various TCGA tumor types. Our findings highlight high *ESR2* expression as a significant positive prognostic factor in nine tumor types: BLCA, BRCA, CESC, ESCA, HNSC, LUAD, OV, PAAD, and THYM. Previous data are inconsistent regarding our analysis. For BLCA, discrepancies noted by Goto et al. underscore variations in immunohistochemical scoring systems and the use of unspecific antibodies [87]. In BRCA, while previous mRNA-based studies confirm our results [69,88], certain protein-based studies suggested that ERβ expression was correlated with poor prognosis [89,90]. Similarly, conflicting findings occur in CESC, with some reporting ERβ expression solely in invasive cervical tumors [91], while others find no ERβ expression in cervical cancer cells [92]. Despite the lack of a clear link between ERβ and CESC, data indicate its potential role in cervical carcinogenesis. For ESCA, Wang et al. showed that ERβ expression may predict a better outcome for patients [93], confirming our results. HNSC comprises different types of malignancies affecting many sites, including the oral cavity and larynx [94]. Studies indicate that ERβ-positive patients with oropharyngeal cancer tend to have longer 5-year survival than ERβ-negative patients [95]. Still, conflicting reports suggest ERβ’s role in cancer progression through enhanced proliferation and invasion in laryngeal carcinoma [96]. These inconsistencies may arise from the heterogeneity of HNSC itself and variations in methodologies, such as the use of unspecific antibodies, underscoring the need for further investigation in this area [22,23]. While our analysis suggests ERβ as a positive factor in lung cancer, the protein-based meta-analysis of Li et al. showed no significant correlations between receptor expression and clinicopathological features [97]. Nonetheless, ERβ is the main ER expressed in lung tumor specimens [82,98,99,100], suggesting its potential significance in estrogenic response in LUAD [82]. Additionally, the intracellular localization of ERβ emerged as a critical determinant of its activity in OV. Cytoplasmic ERβ was associated with poorer outcomes and chemoresistance in OV, whereas nuclear ERβ did not correlate with either OS or DFS [101]. However, it is worth noting that the mentioned IHC analysis was performed on a relatively small number of patient samples and required validation on a transcriptomic level [101]. In terms of PAAD, studies suggest that ERβ might play a significant role in the estrogen-dependent proliferation of pancreatic cancer cells in vitro [102]. Contrary to our findings, some studies present ERβ as a negative prognostic factor in pancreatic ductal adenocarcinoma (PDAC) [80]. It is important to note that these findings were based on protein data and conducted with antibodies of uncertain specificity, as highlighted by Andersson et al. [22] and Nelson et al. [23]. Moreover, estrogen’s impact on the thymus is well documented, including its role in the inhibition of postnatal thymocyte development [103,104]. While many studies have emphasized ERα-dependent estrogen signaling in the thymus [105,106,107], other studies support ERβ overexpression in THYM [108,109] and its role as a significant positive factor in terms of OS [103,110], which is in line with our analysis.

Moreover, we observed that low *ESR2* expression is associated with longer OS in four tumor types: KIRP, LAML, MESO, and THCA. Notably, some studies indicated that ERβ may act as a tumor suppressor in kidney carcinomas, with others correlating higher expression of this receptor with worse survival outcomes [71,111,112]. Our results coincide with the latter. Further, a meta-analysis of gene signatures in acute myeloid leukemia did not establish a direct link between *ESR2* expression and patient survival. However, it was noted that high ERβ/ERα ratios were necessary for anti-leukemic effects of ERβ signaling and that ERβ could suppress leukemogenesis [113]. Here, we present low *ESR2* expression as a favorable factor. Therefore further research is required to evaluate whether this receptor affects OS in LAML. Regarding MESO, studies have linked high ERβ expression with better prognosis in OS [114,115]. Nevertheless, Pillai et al. [116] demonstrated that nuclear presence was a favorable factor in terms of OS in MESO, whereas the cytoplasmic fraction of this receptor was associated with poor survival [116]. In THCA, many studies emphasized the importance of the ERα/ERβ ratio on mRNA and protein levels and its influence on clinical outcomes. However, there is a lack of analyses regarding the specific effect of individual *ESR2* expression [117].

Furthermore, we analyzed *ESR2* mRNA expression in correlation with disease-free survival, yielding significant findings across six tumor types. High *ESR2* expression emerged as a favorable prognostic indicator for DFS in CHOL, LUSC, and TGCT. To date, ERβ was found to mainly govern estrogen signaling in CHOL [118], manifesting anti-proliferative actions in extrahepatic CHOL. Other CHOL-related studies focused on the ERα/ERβ ratio’s impact on clinical outcomes [119,120]; therefore, the singular influence of ERβ has not been analyzed further so far. Moreover, many studies, summarized in [121], associated ERβ with a favorable prognosis, consistent with our findings for LUSC. However, few reports indicate ERβ’s involvement in chemoresistance and the promotion of invasion in LUSC [122]. To the best of our knowledge, the TGCT-related literature does not directly associate *ESR2* mRNA expression with survival outcomes.

The remaining three DFS-related results from our analysis indicated a correlation between low *ESR2* mRNA expression and extended DFS in GBM, LGG, and PRAD. However, contradictory previous studies have shown that loss of ERβ occurs during glial neoplasm progression [123,124], and low ERβ expression has been associated with poorer survival [125]. With regard to PRAD, Zellweger et al. showed that ERβ expression was associated with poor clinical outcomes in hormone-sensitive prostate cancer [126]. It is important to note that ERβ has also been presented as a protective factor in prostate cancer progression [127]. However, some studies show contradictory results [128], most likely due to the use of different antibodies and receptor isoforms. Lee et al. showed that ERβ’s isoforms also play an essential role in PRAD [24]: ERβ1 was assessed as an anti-proliferative factor, whereas ERβ2 and ERβ5 were continuously expressed in high-grade prostate tumors [129].

Moreover, our analyses revealed that both OS and DFS were influenced by *ESR2* mRNA expression levels in ACC, DLBC, KICH, KIRC, and STAD. Previous studies have shown that ERβ predominates in adrenal glands, with lower expression in ACC [130]. In our analysis, we observed that elevated *ESR2* mRNA expression in females with ACC was associated with longer OS and DFS, suggesting the tumor-suppressive role of ERβ [109,131]. Similarly, we found elevated *ESR2* expression as a positive prognostic factor in DLBC, aligning with the existing literature associating ERβ activation with tumor growth inhibition [132]. In contrast, low ESR2 expression in KICH and KIRC correlated with better survival and prognosis [133], as in our study. Concerning STAD, low *ESR2* expression in male patients was noted as a determinant of better OS and DFS. Xu et al. demonstrated that while *ESR2* mRNA levels did not correlate with any clinicopathological parameters, the absence of ERβ at the protein level was associated with poor OS [134].

As a next step, we conducted GSEA of 30 tumor types to elucidate the role of *ESR2* in molecular pathways. We found significant results in several key pathways, including epithelial–mesenchymal transition (EMT), hypoxia response, and cell cycle, among others. In PRAD, our findings align with previous research demonstrating EMT-repressing activity associated with ERβ1 [135]. Moreover, previous studies suggested that ERβ is implicated in the cellular response to hypoxic conditions, one of the hallmarks of solid tumors [136,137,138,139]. Our analysis corroborated these findings by revealing a significant enrichment of *ESR2* expression in Hallmarks’ “Hypoxia” gene set, suggesting its importance in response to lowered oxygen levels in PRAD. We observed negative NES values in cell cycle-related sets that are potentially dependent on *ESR2* expression, such as results for COAD. This aligns with previous research indicating ERβ as a tumor suppressor by arresting the cell cycle and promoting the apoptosis pathway in colorectal cancer [140].

To further investigate the role of *ESR2* in tumors, we searched for co-expressed genes exhibiting expression patterns like *ESR2* and possessing estrogen-related regulatory elements. We identified 12 potential genes associated with *ESR2* expression in cancer tissue, previously not characterized in terms of *ESR2*. Subsequently, we assessed the combined influence of those genes and *ESR2* on clinical outcomes in patients with TCGA tumor types as they did not present any impact on OS or DFS on their own. While our target genes have been described in the literature, they have primarily been discussed without explicit connections to *ESR2* expression. In MESO and UCEC, concurrent low expression of *ESR2* and *MDM4* showed a combined positive effect on OS. This aligns with the established literature showing that high *MDM4* expression tends to correlate with poorer prognosis in many cancer types as it is known to inhibit tumor suppressor activity of p53 in various cancer types [141,142,143]. In LUSC, *MDM4* is typically highly expressed [144], as shown in our analysis. We show that low *OCIAD2* and high *ESR2* expression correlated with longer DFS in LUSC. *OCIAD2* may indirectly exert tumor-promoting activities, and its downregulation led to the loss of mitochondrial structure and an overall decrease in proliferation and invasion in lung cancer [145,146]. However, there is a lack of studies showcasing a combined effect with ERβ.

Moreover, in BRCA, the simultaneous high expression of *ESR2* and *NDUFB3* correlated with longer DFS [69,88,89,90]. *NUDFB3* expression has been shown to elevate mitochondrial ROS production, leading to apoptosis through the JNK signaling pathway and cycle arrest in HCC [147]. Similarly, in LUSC, it was shown that *CFL1* mRNA expression increased with tumor growth, while the protein level of cofilin translated from *CFL1* diminished, suggesting a posttranslational regulatory mechanism [148].

Conversely, in CHOL, we observed that combined high expression of *ESR2* and low expression of *NDUFB3* was associated with better DFS. In contrast, previous studies suggested that solely elevated *ESR2* expression is a positive indicator of clinical outcomes [118]. The combined impact with *NDUFB3* has not been previously reported. Furthermore, our analysis revealed a significant influence on OS in PRAD of the low-level co-expression of *ESR2* and *ACIN1*. Notably, *ACIN1* expression has been linked to bicalutamide resistance, a first-generation drug used in androgen deprivation therapy for prostate cancer [149]. In previous studies, elevated levels of *CFL1* were correlated with an increased risk of lymph node metastasis and a deeper rate of local invasion in colon cancer [150]. Contrarily, we show that low *ESR2* and high *CFL1* expression were associated with better OS and DFS in COAD and improved OS in LUSC. Regarding DFS, we observed that high *ESR2* and low *FNBP4* expression correlated with positive outcomes in PAAD. While ERβ has been suggested as an adverse prognostic factor in PAAD [80], the role of *FNBP4* remains relatively unexplored. To date, *FNBP4* expression has been correlated with poor OS in hepatocellular carcinoma [151]. Moreover, ERβ’s exact role in LUSC and PRAD has not been unequivocally determined [80,126,127,128,152]. However, *FNBP4* possesses in its promoter region the canonical sequence of ERE, which may suggest its direct regulation by ERβ. Our study also demonstrated another correlation: in UCEC, low ESR2 and high OCIAD2 expression were associated with longer OS. Since *OCIAD2* expression was associated with adverse clinical outcomes in previous studies [153], our results linking it to *ESR2* require further analysis to unravel potential mechanisms of interaction. Studies have shown that the downregulation of *SYNE2* inhibits endothelial cell migration and may play a role in angiogenesis [154], while other reports indicate that *SYNE2* depletion is significantly correlated with increased muscle cell proliferation [155]. Interestingly, in CHOL and TGCT, simultaneous high expression levels of *ESR2* and *SYNE2* improved patients’ clinical outcomes. At the same time, high *ESR2* and low *SYNE2* expression were significantly associated with longer DFS in CESC and LUSC, while an inverse relationship between those factors was noted in COAD, MESO, SARC, and SKCM. Therefore, the *ESR2* regulation of *SYNE2* expression could be tumor-dependent and requires further functional studies.

While previous studies have shown that *ACIN1* may activate proapoptotic signaling pathways in colorectal cancer, we hypothesize that it may work synergistically together with *ESR2*, a known tumor suppressor in CRC [156,157]. Additionally, *MDM4* and *ESR2* have been reported to be upregulated in HNSC [75,158]. The precise role of ERβ in this context remains ambiguous, warranting a cautious interpretation of our results. In COAD, we observed a simultaneous low expression of *ESR2* and *OCIAD2* as a favorable factor in DFS. ERβ, a known tumor suppressor in COAD [140], may regulate *OCIAD2* expression, potentially leading to mitochondria-related apoptosis [145]. Another identified connection relates to BLCA patients exhibiting simultaneous high *ESR2* and low *PLIN3* expression, which presented better clinical outcomes. Previous studies linked high *PLIN3* expression with shorter survival time in other tumor types, such as lung adenocarcinoma [159]. Furthermore, POU2AF1 expression used to be considered lymphocyte-restricted. However, some studies showcase the expression of POU2AF1 in normal human airway epithelium and lung adenocarcinoma tissue [160,161]. In LUSC and BLCA, we presented that concomitant high expressions of *ESR2* and *POU2AF1* were associated with extended DFS. On the other hand, simultaneous low expression of *ESR2* and *POU2AF1* was associated with prolonged OS in MESO.

Furthermore, another *ESR2*-correlated gene was *TMEM141*, which encodes a transmembrane protein, likely involved in protein binding [162,163] and neural development [164]. However, data regarding *TMEM141*’s role in cancer are scarce. Additionally, in our analysis, the co-expression of *TNFRSF13C* and *ESR2* was associated with OS or DFS in BLCA, COAD, PAAD, SARC, and SKCM. We hypothesize a regulatory link between those two factors, with a possible influence on tumor-infiltrating B cells, as *TNFRSF13C* has been demonstrated to prevent apoptosis by inhibiting Bim proteins and enhancing mitochondrial activity, thereby prolonging B-cells’ cellular life span [165,166,167,168]. The last interesting identified co-expressed gene was RAC1.

While previous studies have linked elevated *RAC1* protein levels with high metastasis of lung tumor cells [169], we observed that low *ESR2* and high *RAC1* expression were associated with longer OS in LUSC. Conversely, in COAD, we demonstrated that the simultaneous low expression of *ESR2* and *RAC1* correlates with longer OS and DFS. Previous studies linked dysregulated RAC1 expression with tumor initiation, progression, and metastasis in cases of gastric, testicular, and breast cancers [170], establishing our results in COAD in line with the literature.

In summary, we comprehensively analyzed *ESR2* and selected co-expressed gene expression in TCGA tumor types. Despite the depth of the analysis, a notable limitation of our study was its reliance solely on transcriptomic data. However, the lack of this element was related to inconsistencies with regard to ERβ’s role in carcinogenesis in various tumor types, along with the method of detection of the protein. We intended to compare transcriptomic and proteomic data; therefore, we checked the Human Protein Atlas database. Notably, HPA offers two types of antibodies for Erβ: HPA068406 (Atlas Antibodies, Sigma-Aldrich, Milwaukee, WI, USA) and CAB079300 (R&D Systems). HPA068406 is a polyclonal rabbit antibody validated in the HPA database through immunocytochemistry, Western blot, and protein array, while CAB079300 is a monoclonal mouse antibody validated in HPA by immunohistochemical staining. Moreover, according to HPA, CAB079300 has shown inconsistencies, particularly in Western blot analysis, contradictory to Andersson et al. [22] and Nelson et al. [23]. Furthermore, HPA antibody HPA068406 fails to detect ERβ in healthy breast tissue samples, contradicting several studies [69,171]. Given the disparity between transcriptomic data from TIMER2.0 and immunostaining data from HPA, we could not perform a comparative analysis using protein data; hence, we focused on TCGA datasets. Additionally, posttranscriptional or posttranslational modifications that alter mRNA and protein levels of *ESR2*/ERβ could play a significant role. Therefore, the abundance of *ESR2* mRNA might not be reflected in protein presence or activity. For this reason, we think a proteomic analysis is the next step to understanding ERβ’s activities and responsibilities in cancer, all while taking into consideration ERβ’s isoforms and the specificity of antibodies used in research, likewise mentioned before. Still, this study constitutes a compact and comprehensive analysis of *ESR2* mRNA expression levels across diverse tumor types and an exploration of its potential functional implications.

## 4. Materials and Methods

### 4.1. Transcriptomic and Proteomic Data

All transcriptomic and proteomic data are available online, and the access is neither restricted nor requires patients’ consent. Transcriptomic data were downloaded from cBioPortal (https://www.cbioportal.org/, accessed on 1 February 2024) [172,173,174]. We used the Firehose Legacy datasets (previously known as Provisional datasets) divided by tumor type. The RNA sequencing-based mRNA expression data (RNASeq V2) were normalized using RSEM, resulting in mRNA expression z-scores, as described in [175]. Specific names of datasets and the numbers of patients in each dataset are available in Supplementary Table S1. Patients without full clinical data used in this study were excluded. Additional transcriptomic data were obtained from the Human Protein Atlas [176,177] (https://www.proteinatlas.org/ accessed on 1 February 2024) and TIMER2.0 [178,179,180] (http://timer.comp-genomics.org accessed on 1 February 2024).

Proteomic data were downloaded from the Human Protein Atlas (HPA), a database allowing for a genome-wide analysis of human proteins. Data were obtained through a phrase search (“ESR2”) and with the use of downloadable data from the “Data” tab, which was searched for genes of interest.

### 4.2. Clinical Data

All clinical data were downloaded from cBioPortal [172,173,174]. We used the OncoPrint tab to access clinical data, selecting characteristics from “Tracks” options, including overall survival status, overall survival time, disease-free survival status, disease-free survival time, and sex. Combined transcriptomic data and clinical features were used to plot Kapplan–Meier curves for overall survival and disease-free survival. Additional clinical data were acquired from TISIDB [181] (http://cis.hku.hk/TISIDB/ accessed on 1 February 2024).

### 4.3. Databases and Bioinformatic Tools

#### 4.3.1. cBioPortal

We used the “Co-expression” tab in cBioPortal [172,173,174] to identify similarly co-expressed genes with regard to *ESR2*. Spearman’s correlations (*rho*) were calculated and results with a *p*-value < 0.05 and FDR < 0.05 were considered significant. Gene lists were downloaded and searched for the highest correlation coefficient. Target genes were selected based on the highest repeatability and the highest *rho* scores.

#### 4.3.2. TIMER2.0

TIMER2.0 [178,179,180] was used to access transcriptomic data regarding tumors and corresponding normal tissue samples. Through the search bar (phrase “ESR2”) and “Cancer Exploration” features, we were able to generate plots presenting differential gene expression between tumor and normal tissue samples (“Gene_DE” tab). To validate correlations calculated based on cBioPortal data, we used the “Gene_Corr” tab to generate Spearman’s correlations between *ESR2* and selected genes. Results with a *p*-value < 0.05 and FDR < 0.05 were considered significant.

#### 4.3.3. Human Protein Atlas

The Human Protein Atlas (HPA) [176,177] database was searched with the phrase “ESR2” to acquire proteomic and transcriptomic data across tissue types. Protein and RNA expressions in tissue profiles were analyzed using the “Tissue” feature. The “Pathology” feature was used to search cancer-related data in HPA. Data from the “Downloadable data” tab were acquired and analyzed with regard to *ESR2* and target genes.

Overviews of protein expression patterns in tissue samples in the HPA database; thus, the staining scores in immunohistochemistry assays mentioned in this manuscript, were provided by HPA. The guidelines for the classification of immunohistochemical results are described in the “Assays & Annotation” section of the HPA database (https://www.proteinatlas.org/about/assays+annotation#ihk accessed on 1 February 2024), including staining intensity (negative, weak, moderate, strong), fraction of stained cells (<25%, 25–75%, <75%), and subcellular localization (nucleus, cytoplasm, membrane). All the annotations in the HPA database are provided by a specialist, with verification by a second specialist.

#### 4.3.4. TISIDB

In the TISIDB [181] database, we researched associations between *ESR2* expression and clinical features across TCGA tumor types. Data were accessed through the search bar (phrase “ESR2”) and choosing appropriate tabs, including “Clinical”. We used TISIDB tools to generate survival curves and calculate Spearman’s correlations between *ESR2* expression and stage or grade. Results with a *p*-value < 0.05 and FDR < 0.05 were considered significant.

The TISIDB database integrates multiple heterogeneous data types pertaining to tumor and immune system interactions. Genomics, transcriptomics, and clinical data of 30 non-hematologic cancer types were collected from TCGA to present associations between gene expression and clinical features. All classifications were likewise based on TCGA nomenclature, including the stage and grade classifications used in this study.

#### 4.3.5. GSEA

We established Differentially Expressed Gene (DEG) lists based on cBioPortal [172,173,174] transcriptomic data. Cut-off points for *p*-value and FDR were both <0.05. The created DEG lists were used to run a preranked Gene Set Enrichment Analysis. We used the Gene Set Enrichment Analysis (GSEA) [182,183,184] tool to present whether predefined sets of genes showed statistically significant, concordant differences within provided samples. The predefined gene sets were provided by the Molecular Signatures Database (https://www.gsea-msigdb.org/gsea/index.jsp accessed on 1 February 2024). DEG lists were uploaded to the GSEA tool (GSEA software version 4.3.3.) and analyzed through GSEAPreRankedPage. The analysis was computed with default settings: probe sets were created with HUGO gene symbols, so the “No_collapse” option was chosen; the permutation number was set to 1000 and the permutation type “gene-sets” was selected. We used FDR < 0.05 for significant results due to the gene set permutation type of analysis and for gene set size correction.

#### 4.3.6. UCSC Genome Browser

We used the University of Santa Cruz Genome Browser [185] (https://genome.ucsc.edu/index.html accessed on 1 February 2024) to visualize genomic data (based on GRCh38/hg38 [December 2013] human assembly). We searched for transcription sites for estrogen receptor β (ERβ, *ESR2* gene) and hepatocyte nuclear factor 3-α (HNF3α, *FOXA1* gene) in target genes through JASPAR CORE 2022 with default settings regarding score (minimum score = 400) (JASPAR CORE 2022 was the update available at the time of conducting this analysis). Moreover, we searched for co-occurring open chromatin marks near transcription sites for ERβ and HNF3α, namely, epigenetic marks of regulatory elements (H3K4Me1 and H3K27Ac) and promoters (H3K4Me3). We used ENCODE Regulation Layered H3K4Me1, Layered H3K4Me3, and Layered H3K27Ac tracks with default settings as they showed histone marks across the genome based on ChIP-seq data from seven selected cell lines (GM12878, H1-hESC, HSMM, HUVEC, K562, NHEK, NHLF). Each regulatory factor available in ENCODE Regulation tracks was assayed separately; consequently, complete data were attainable for a limited number of cell lines. Target genes with multiple ERβ and HNF3α binding sites co-occurring with open chromatin epigenetic marks were selected as possibly substantial hits for further analysis.

### 4.4. Statistical Analysis

Statical analyses we carried out using PQStat v.1.8.4 software (https://pqstat.pl/ accessed on 5 May 2024). Descriptive statistics of each dataset are demonstrated in Supplementary Table S1. The normality of the data was calculated with the Shapiro–Wilk test (α < 0.05). As all datasets proved to be not normally distributed, the correlations between two variables were calculated with two-tailed Spearman’s rank correlation coefficient (*rho*) (α < 0.05). In GSEA, statistical calculations of NES values were obtained within GSEA analysis software version 4.3.3. Overall survival (OS) and disease-free survival (DFS) analyses were estimated using the Mantel–Cox test (log-rank test) (α < 0.05). Patients were divided into “high” and “low” groups based on expression levels of *ESR2* or target genes. Cut-off points for classification into “high” or “low” expression groups were based on the Evaluate Cutpoints R application algorithm [32], with the “survival” R package (RStudio software version 2024.01.0) as a selected method for stratification of the patient into two groups. Kapplan–Meier plots were graphed with PQStat v.1.8.4 software. In all statistical analyses, results with a *p*-value < 0.05 and FDR < 0.05 were considered significant.

## 5. Conclusions

- *ESR2* mRNA expression differs between cancerous and normal tissue in various TCGA tumor types, including BRCA, COAD, KICH, CHOL, HNSC, and LUSC;
- *ESR2* expression impacts patient survival in several TCGA tumor types, including BLCA, HNSC, THYM, KIRP, and THCA;
- GSEA analysis reveals *ESR2* enrichment in gene sets related to epithelial–mesenchymal transition, hypoxia response, and cell cycle in cancers like PRAD and COAD;
- Twelve genes (*ACIN1*, *CFL1*, *FNBP4*, *MDM4*, *NDUFB3*, *OCIAD2*, *PLIN3*, *POU2AF1*, *RAC1*, *SYNE2*, *TMEM141*, *TNFRSF13C*) were identified as co-expressed with *ESR2* and showing a combined effect with the receptor on patient survival in selected tumors, including BLCA, MESO, BRCA, COAD, and SKCM.

## Figures and Tables

**Table 1:** Characteristics of genes of interest.

Gene	Description	Source
*ACIN1* [14q11.2]	Apoptotic chromatin condensation inducer 1 regulates chromatin condensation after activation by caspase-3 during apoptosis. Additionally, it may be involved in mRNA regulation after splicing.	[33]
*ARPC2* [2q35]	Actin-related protein 2/3 complex subunit 2 mediates actin polymerization in the nucleus and therefore regulates transcription and homolog recombination in response to DNA damage.	[34,35]
*CELF6* [15q23]	CUGBP elav-like family member 6 regulates pre-mRNA alternative splicing and may be involved in mRNA editing and translation.	[36]
*CFL1* [11q13.1]	Cofilin 1 carries out F-actin depolymerization, thus regulating cell morphology through cytoskeletal organization in epithelial cells.	[37,38]
*COL4A3* [2q36.3]	The collagen α-3(IV) chain is one of the structural components of glomerular basement membranes. Tumstatin within this domain presents anti-tumor activity.	[39,40]
*FAM30A* [14q32.33]	Family with sequence similarity to 30 member A and may be a non-coding RNA.	[41]
*FCRL5* [1q23.1]	Fc receptor-like 5 is most likely involved in B-cell differentiation and may present an immunoregulatory role in marginal-zone B-cells.	[42]
*FNBP4* [11p11.2]	Formin-binding protein 4 may be involved in the regulation of cytoskeletal dynamics during cell division and migration.	[43]
*LENG8* [19q13.42]	Leukocyte receptor cluster member 8 encodes leukocyte-expressed receptors of the immunoglobulin superfamily.	[44]
*MDM4* [1q32.1]	MDM4 regulator of p53 inhibits p53- and p73-mediated cell cycle arrest and apoptosis and inhibits the degradation of MDM2.	[45,46]
*NDUFB3* [2q33.1]	NDAH–ubiquinone oxidoreductase subunit B3 is part of the electron transport chain of mitochondria on the inner membrane of the mitochondrion.	[47]
*OCIAD2* [4p11]	Ovarian carcinoma immunoreactive antigen domain containing 2 in one of the mitochondrial respiratory chain complex assembly factors.	[48]
*PLIN3* [19p13.3]	Perilipin 3 is a structural component of lipid droplets required for lipid storage in cells and is involved in mannose 6-phosphate receptor transport.	[49,50]
*POU2AF1* [11q23.1]	POU class 2 homeobox associating factor 1 is a transcriptional coactivator associated with POU2F1/OCT1 or POU2F2/OCT2 complexes; likewise, it is essential for B-cells’ ability to respond to antigens.	[51,52]
*PPP1R3E* [14q11.2]	Protein phosphatase 1 regulatory subunit 3E is predicted to be involved in the positive regulation of the glycogen biosynthetic process and to be a part of the protein phosphatase type 1 complex.	[53]
*RAC1* [7p22.1]	Rac family small GTPase 1 is a plasma membrane-associated protein binding to effector proteins involved in secretion, phagocytosis, migration, and differentiation.	[54,55,56]
*RAP1GDS1* [4q23]	Rap1 GTPase-GDP dissociation stimulator 1 is a guanine nucleotide exchange factor in the GDP-GTP dissociation–binding sequence.	[57,58]
*SYNE2* [14q23.2]	Spectrin repeat containing nuclear envelope protein 2 (Nesprin-2) is a component of the LInker of Nucleoskeleton and Cytoskeleton (LINC) and regulates the spatial organization of intracellular components.	[59]
*TMEM141* [9q34.3]	Transmembrane protein 141 is predicted to be an integral component of the membrane.	[60]
*TNFRSF13C* [22q13.2]	Tumor necrosis factor receptor superfamily member 13C is a B-cell-specific receptor that promotes the survival of mature B-cells and the B-cell response.	[61,62]
*TPTEP2* [22q13.1]	TPTE pseudogene 2 is a transmembrane phosphoinositide 3-phosphatase and tensin homolog 2 pseudogene.	[63]
*VAMP1* [12p13.31]	Vesicle-associated membrane protein 1 is involved in the targeting and fusion of transport vesicles to their target membrane.	[64,65]
*ZBTB25* [14q23.3]	Zinc finger- and BTB domain-containing 25 is predicted to be involved in the regulation of transcription by RNA polymerase II.	[66]
*ZFYVE26* [14q24.1]	Zinc finger FYVE-type-containing 26 encodes protein targeted to membrane lipids through interaction with phospholipids in the membrane.	[67]

## Data Availability

All transcriptomic and proteomic data are openly available online, and the access is neither restricted nor requires patients’ consent. Transcriptomic data were downloaded from cBioPortal (https://www.cbioportal.org/ accessed on 1 February 2024) (Firehose Legacy datasets divided by tumor type). Additional transcriptomic data were obtained from the Human Protein Atlas (https://www.proteinatlas.org/ accessed on 1 February 2024) and TIMER2.0 (http://timer.comp-genomics.org accessed on 1 February 2024). Additional clinical data were acquired from TISIDB (http://cis.hku.hk/TISIDB/ accessed on 1 February 2024). The predefined gene sets used in GSEA were provided by the Molecular Signatures Database (https://www.gsea-msigdb.org/gsea/index.jsp accessed on 1 February 2024). The University of Santa Cruz Genome Browser (https://genome.ucsc.edu/index.html accessed on 1 February 2024) was used to visualize genomic data.

## Supplementary Materials

The following supporting information can be downloaded at https://www.mdpi.com/article/10.3390/ijms25168707/s1.

## Abbreviations

ACC	Adrenocortical carcinoma
BLCA	Bladder urothelial carcinoma
BRCA	Breast invasive carcinoma
CESC	Cervical squamous cell carcinoma and endocervical adenocarcinoma
CHOL	Cholangio carcinoma
COAD	Colon adenocarcinoma
DLBC	Lymphoid neoplasm diffuse large B-cell lymphoma
ESCA	Esophageal carcinoma
GBM	Glioblastoma multiforme
HNSC	Head and neck squamous cell carcinoma
KICH	Kidney chromophobe
KIRC	Kidney renal clear cell carcinoma
KIRP	Kidney renal papillary cell carcinoma
LAML	Acute myeloid leukemia
LGG	Brain lower-grade glioma
LIHC	Liver hepatocellular carcinoma
LUAD	Lung adenocarcinoma
LUSC	Lung squamous cell carcinoma
MESO	Mesothelioma
OV	Ovarian serous cystadenocarcinoma
PAAD	Pancreatic adenocarcinoma
PCPG	Pheochromocytoma and paraganglioma
PRAD	Prostate adenocarcinoma
READ	Rectum adenocarcinoma
SARC	Sarcoma
SKCM	Skin cutaneous melanoma
STAD	Stomach adenocarcinoma
TGCT	Testicular germ cell tumor
THCA	Thyroid carcinoma
THYM	Thymoma
UCEC	Uterine corpus endometrial carcinoma
UCS	Uterine carcinosarcoma
UVM	Uveal melanoma
