# Supplementary material for: Genes Co-Expressed with ESR2 Influence Clinical Outcomes in Cancer Patients: TCGA Data Analysis
Source: Int J Mol Sci. 2024 Aug 9;25(16):8707. doi: 10.3390/ijms25168707 (PMC11354723; doi:10.3390/ijms25168707)
Supplement: Supplementary file 1 [file ijms-25-08707-s001.zip › ijms-3100484-supplementary.pdf]

Table S1 Descriptive statistics of TCGA Firehose datasets.

Dataset name	Abbreviation	N	ESR2 expression median	ESR2 expression SD	Number of female patients	Number of male patients	Numer of living patients	Number of deceased patients	Number of "high" ESR2 expression patients	Number of "low" ESR2 expression patients
TCGA Adrenocortical Carcinoma	ACC	79	58,7879	91,728312	48	31	51	28	60	19
TCGA Bladder Urothelial Carcinoma	BLCA	408	16,042	27,798441	107	301	228	180	230	178
TCGA Breast Invasive Carcinoma	BRCA	1093	6,82	7,060509	1081	12	941	152	591	502
TCGA Cervical Squamous Cell Carcinoma and Endocervical Adenocarcinoma	CESC	304	15,3559	19,51145	304	0	232	72	195	109
TCGA Cholangiocarcinoma	CHOL	36	10,6767	30,787483	20	16	18	18	16	20
TCGA Colorectal Adenocarcinoma	COAD	376	7,8347	6,779418	169	207	289	87	95	281
TCGA Lymphoid Neoplasm Diffuse Large B-cell Lymphoma	DLBC	48	93,3085	151,390575	26	22	39	9	31	17
TCGA Esophageal Carcinoma	ESCA	184	24,0476	26,865081	26	158	107	77	158	26
TCGA Glioblastoma Multiforme	GBM	158	11,02095	131,366255	55	103	30	128	39	120
TCGA Head and Neck Squamous Cell Carcinoma	HNSC	520	11,9081	21,420069	136	384	299	221	294	226
TCGA Kidney Chromophobe	KICH	66	6,9306	9,019335	27	39	56	10	11	55
TCGA Kidney Renal Clear Cell Carcinoma	KIRC	533	9,9469	40,319841	188	345	358	175	121	412
TCGA Kidney Renal Papillary Cell Carcinoma	KIRP	290	12,00655	20,072185	76	214	246	44	82	208
TCGA Acute Myeloid Leukemia	LAML	173	24,3261	81,303782	80	93	59	114	70	103
TCGA Brain Lower Grade Glioma	LGG	514	10,42165	26,084918	230	284	389	125	71	443
TCGA Liver Hepatocellular Carcinoma	LIHC	371	2,8285	11,122746	121	250	241	130	250	121
TCGA Lung Adenocarcinoma	LUAD	515	15,5076	15,400088	277	238	328	187	93	422
TCGA Lung Squamous Cell Carcinoma	LUSC	501	18,8202	29,117621	130	371	284	217	147	354
TCGA Mesothelioma	MESO	87	9,6294	26,823981	16	71	13	74	-	-
TCGA Ovarian Serous Cystadenocarcinoma	OV	302	17,0894	31,609624	302	0	120	182	16	286
TCGA Pancreatic Adenocarcinoma	PAAD	178	13,37735	12,308312	80	98	85	93	65	113
TCGA Pheochromocytoma and Paraganglioma	PCPG	179	8,3472	19,413774	101	78	173	6	-	-
TCGA Prostate Adenocarcinoma	PRAD	497	11,0947	14,373509	0	497	487	10	247	250
TCGA Sarcoma	SARC	259	8,1508	39,436564	141	118	259	98	199	60
TCGA Skin Cutaneous Melanoma	SKCM	469	15,134	85,696208	180	289	247	222	89	380
TCGA Stomach Adenocarcinoma	STAD	415	24,1784	29,51259	147	268	252	163	73	342
TCGA Testicular Germ Cell Cancer	TGCT	134	37,80615	26,010471	0	134	130	4	54	80
TCGA Thyroid Carcinoma	THCA	501	8,6429	6,864029	366	135	485	16	141	360
TCGA Thymoma	THYM	120	43,06725	30,447608	57	63	111	9	96	34
TCGA Uterine Corpus Endometrial Carcinoma	UCEC	177	14,8721	21,211166	177	0	144	33	76	101
TCGA Uterine Carcinosarcoma	UCS	57	10,1133	9,419299	57	0	22	35	-	-
TCGA Uveal Melanoma	UVM	80	10,00305	25,051912	35	45	57	23	16	64

FigS1

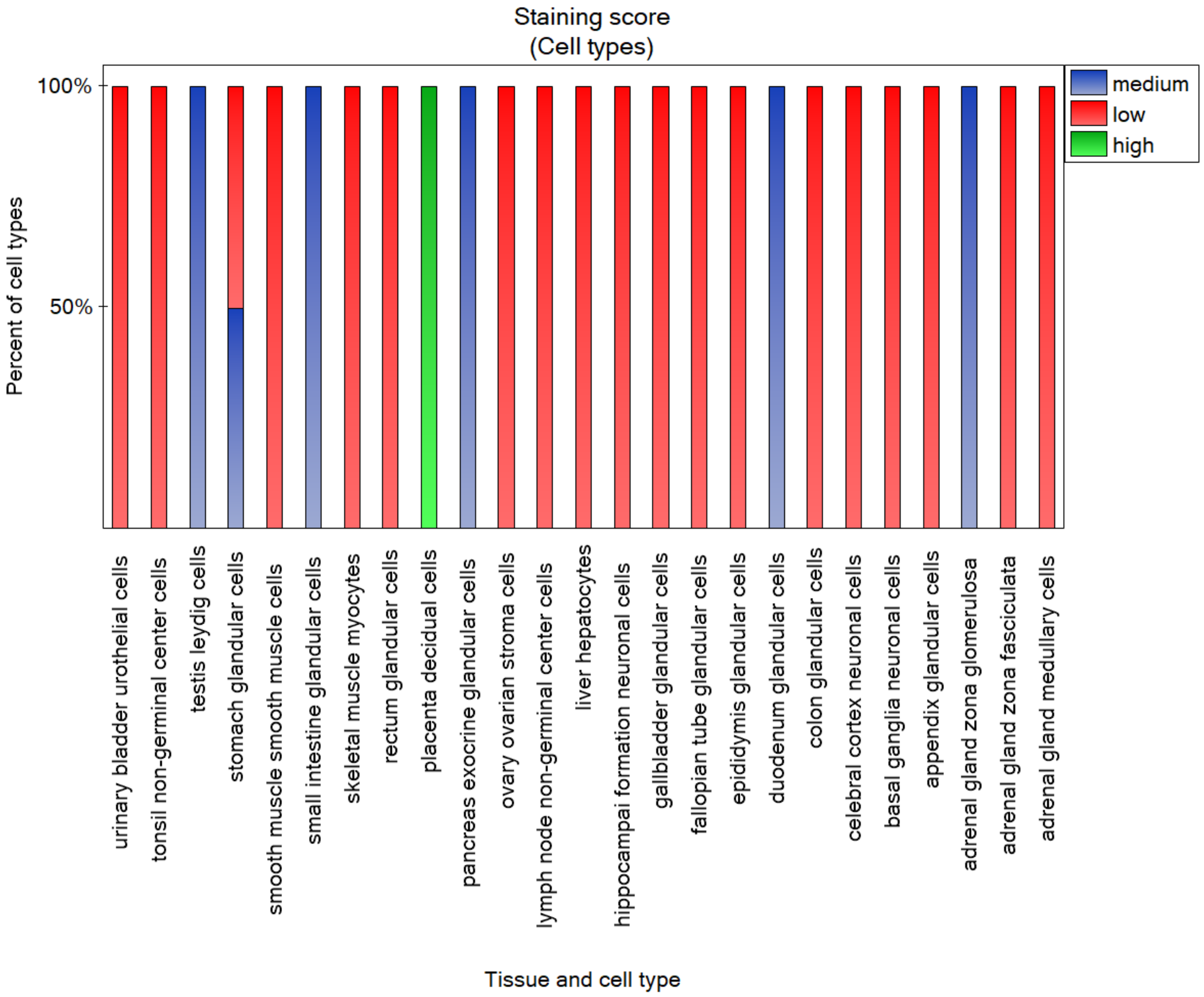

Figure S1 Proteomic analysis of *ESR2* expression across tissues and cell types (based on HPA data, CAB079300 antibody)

FigS2a

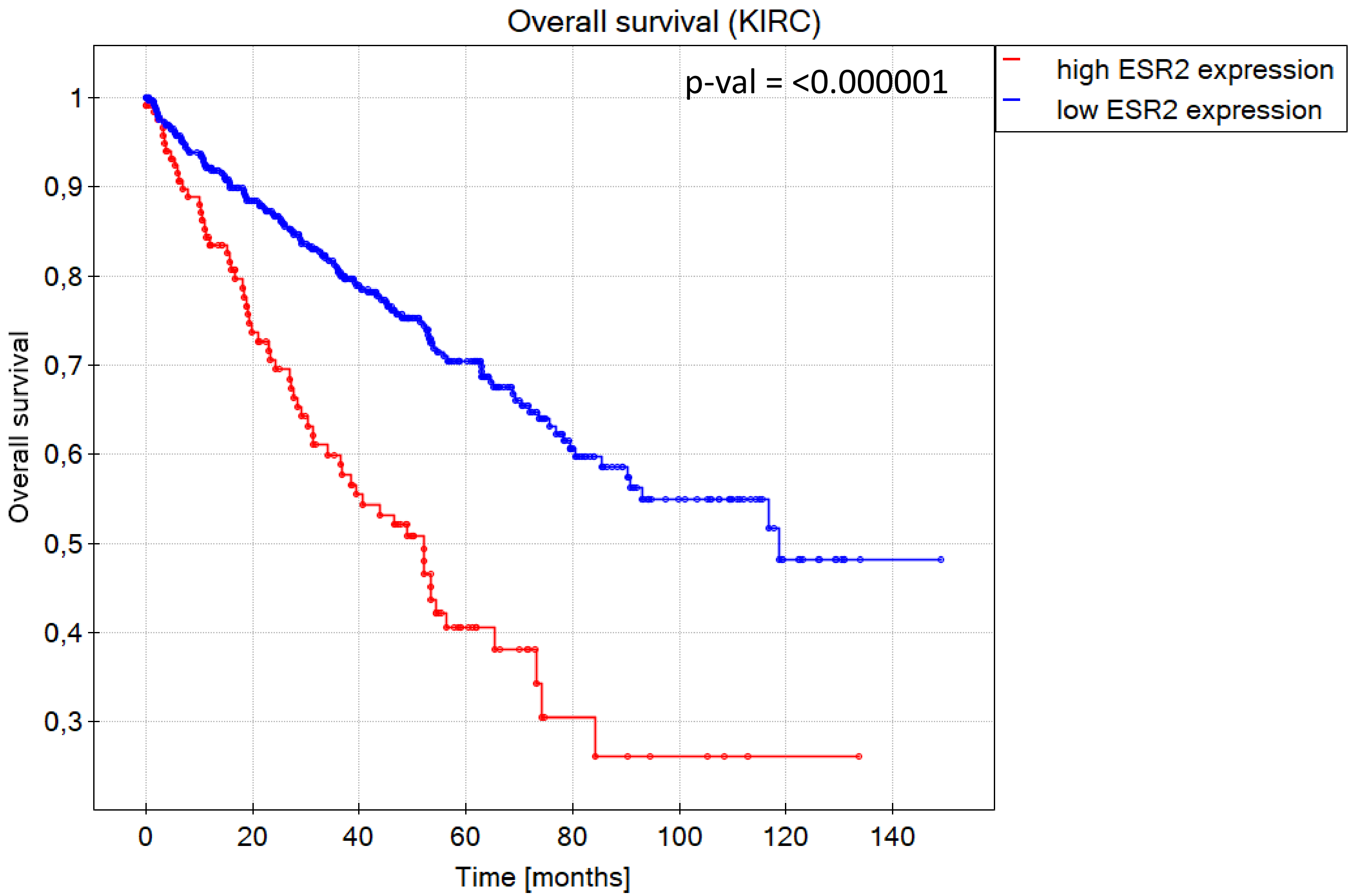

Figure S2 Kapplan-Meier plots of overall survival for TCGA cancer types with *ESR2* expression level as a factor. (a) KIRC, (b) LGG, (c) DLBC, (d) PAAD, (e) BLCA, (f) ACC, (g) ESCA, (h) HNSC, (i) LUAD, (j) KIRP, (k) STAD, (l) THCA, (m) LAML, (n) OV, (o) CESC. p-value <0.05, FDR <0.05

FigS2b

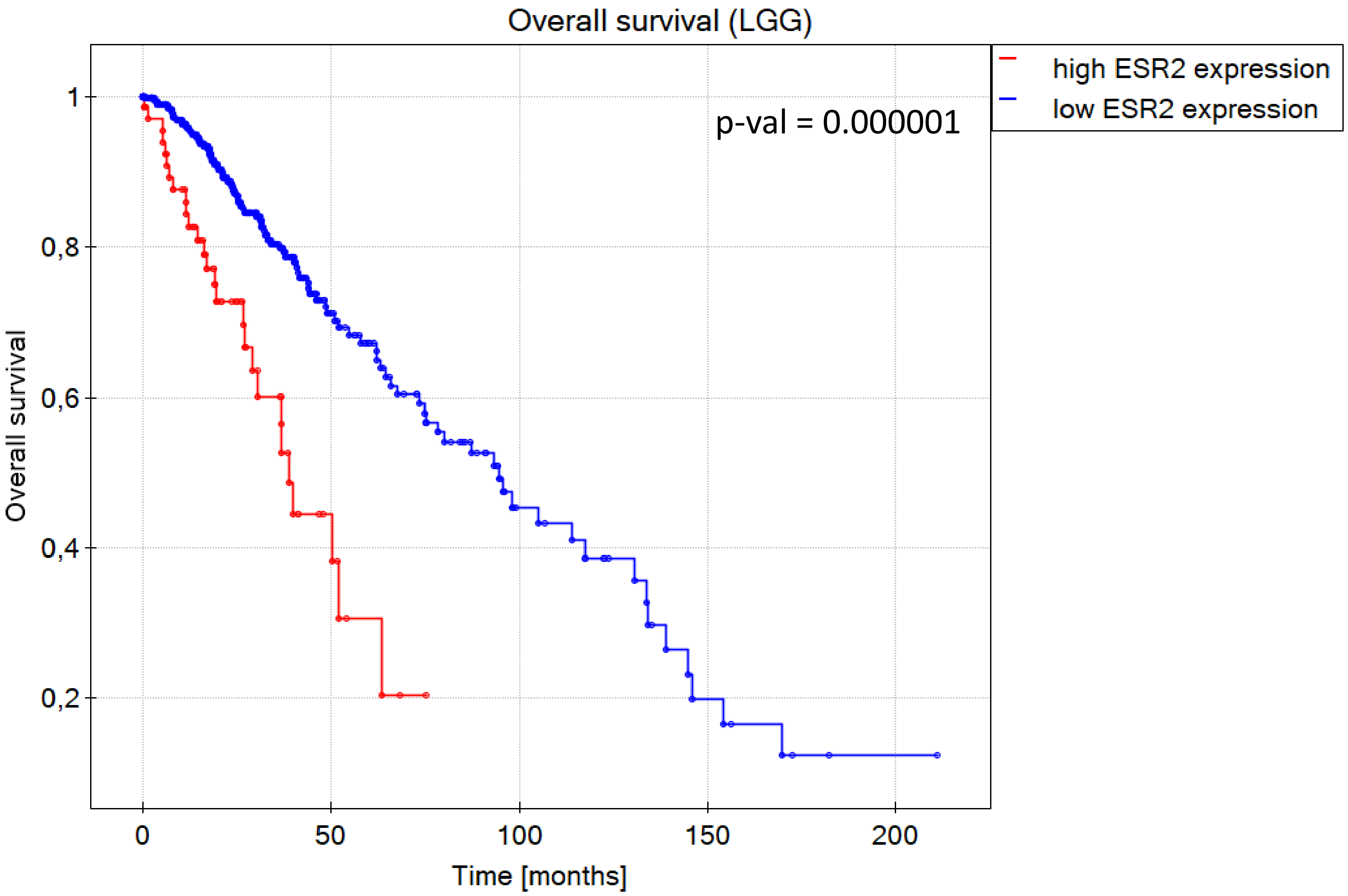

Figure S2 Kapplan-Meier plots of overall survival for TCGA cancer types with *ESR2* expression level as a factor. (a) KIRC, (b) LGG, (c) DLBC, (d) PAAD, (e) BLCA, (f) ACC, (g) ESCA, (h) HNSC, (i) LUAD, (j) KIRP, (k) STAD, (l) THCA, (m) LAML, (n) OV, (o) CESC. p-value <0.05, FDR <0.05

FigS2c

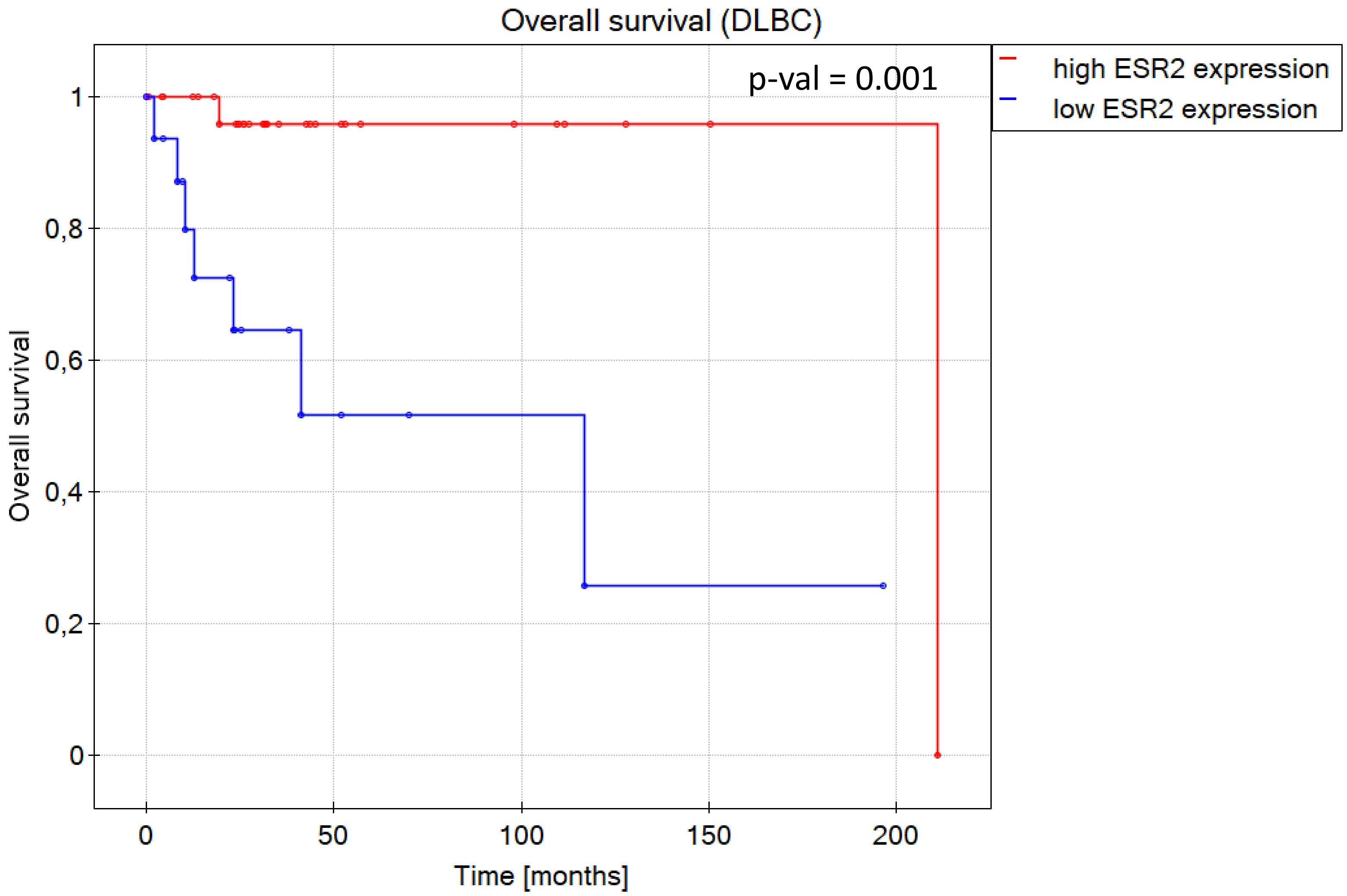

Figure S2 Kapplan-Meier plots of overall survival for TCGA cancer types with *ESR2* expression level as a factor. (a) KIRC, (b) LGG, (c) DLBC, (d) PAAD, (e) BLCA, (f) ACC, (g) ESCA, (h) HNSC, (i) LUAD, (j) KIRP, (k) STAD, (l) THCA, (m) LAML, (n) OV, (o) CESC. p-value <0.05, FDR <0.05

FigS2d

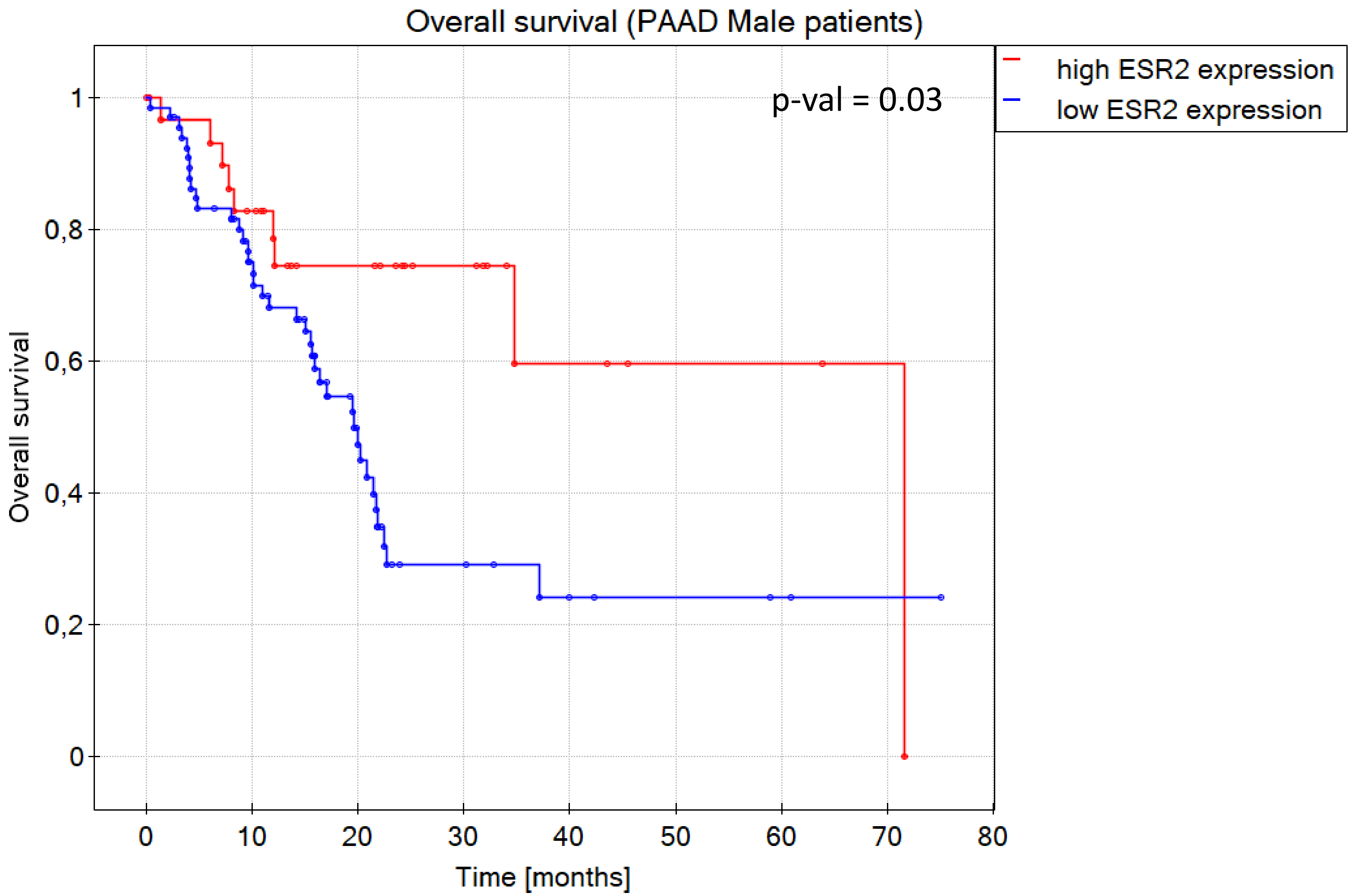

Figure S2 Kapplan-Meier plots of overall survival for TCGA cancer types with *ESR2* expression level as a factor. (a) KIRC, (b) LGG, (c) DLBC, (d) PAAD, (e) BLCA, (f) ACC, (g) ESCA, (h) HNSC, (i) LUAD, (j) KIRP, (k) STAD, (l) THCA, (m) LAML, (n) OV, (o) CESC. p-value <0.05, FDR <0.05

FigS2e

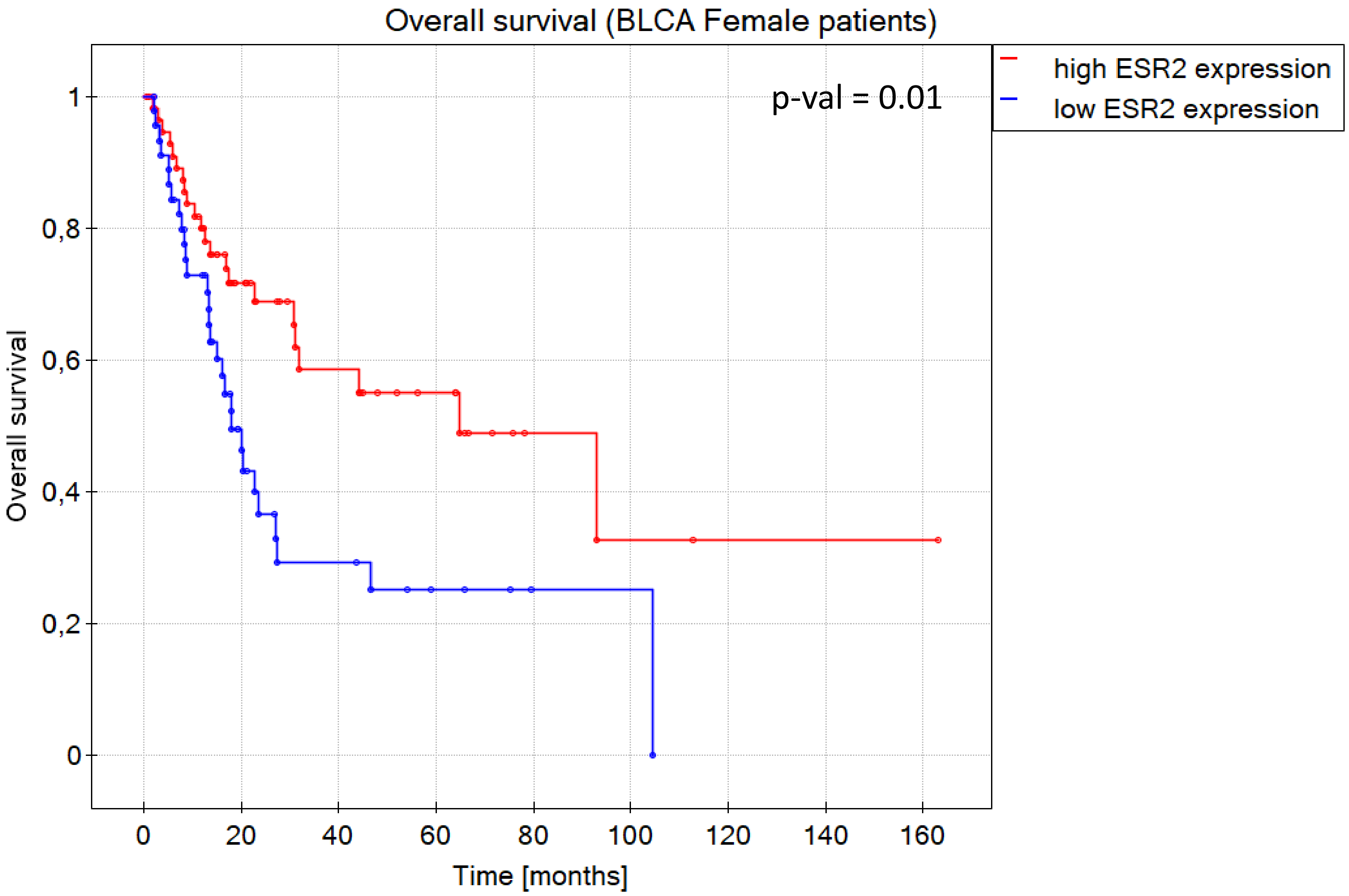

Figure S2 Kapplan-Meier plots of overall survival for TCGA cancer types with *ESR2* expression level as a factor. (a) KIRC, (b) LGG, (c) DLBC, (d) PAAD, (e) BLCA, (f) ACC, (g) ESCA, (h) HNSC, (i) LUAD, (j) KIRP, (k) STAD, (l) THCA, (m) LAML, (n) OV, (o) CESC. p-value <0.05, FDR <0.05

FigS2f

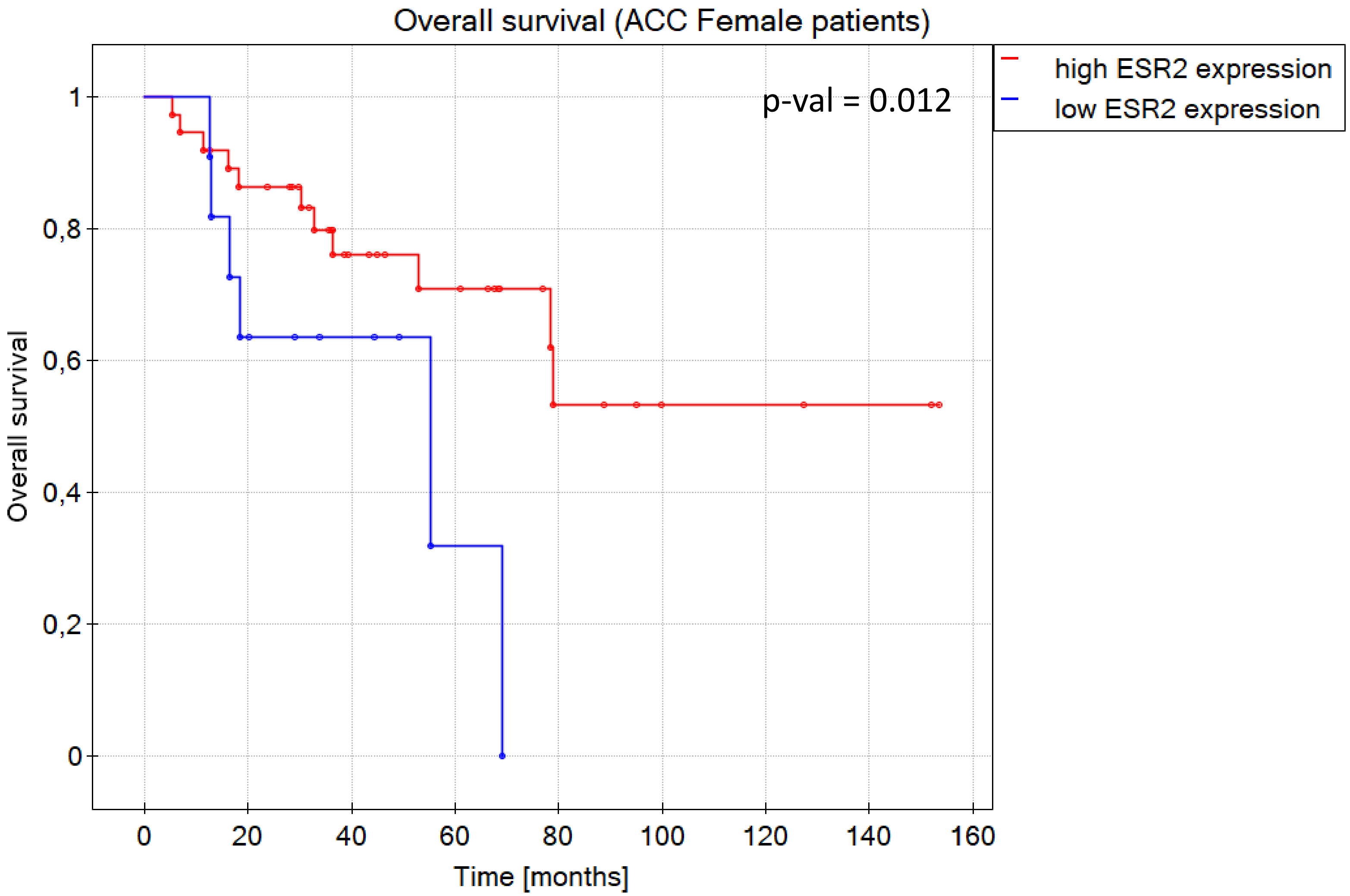

Figure S2 Kapplan-Meier plots of overall survival for TCGA cancer types with *ESR2* expression level as a factor. (a) KIRC, (b) LGG, (c) DLBC, (d) PAAD, (e) BLCA, (f) ACC, (g) ESCA, (h) HNSC, (i) LUAD, (j) KIRP, (k) STAD, (l) THCA, (m) LAML, (n) OV, (o) CESC. p-value <0.05, FDR <0.05

FigS2g

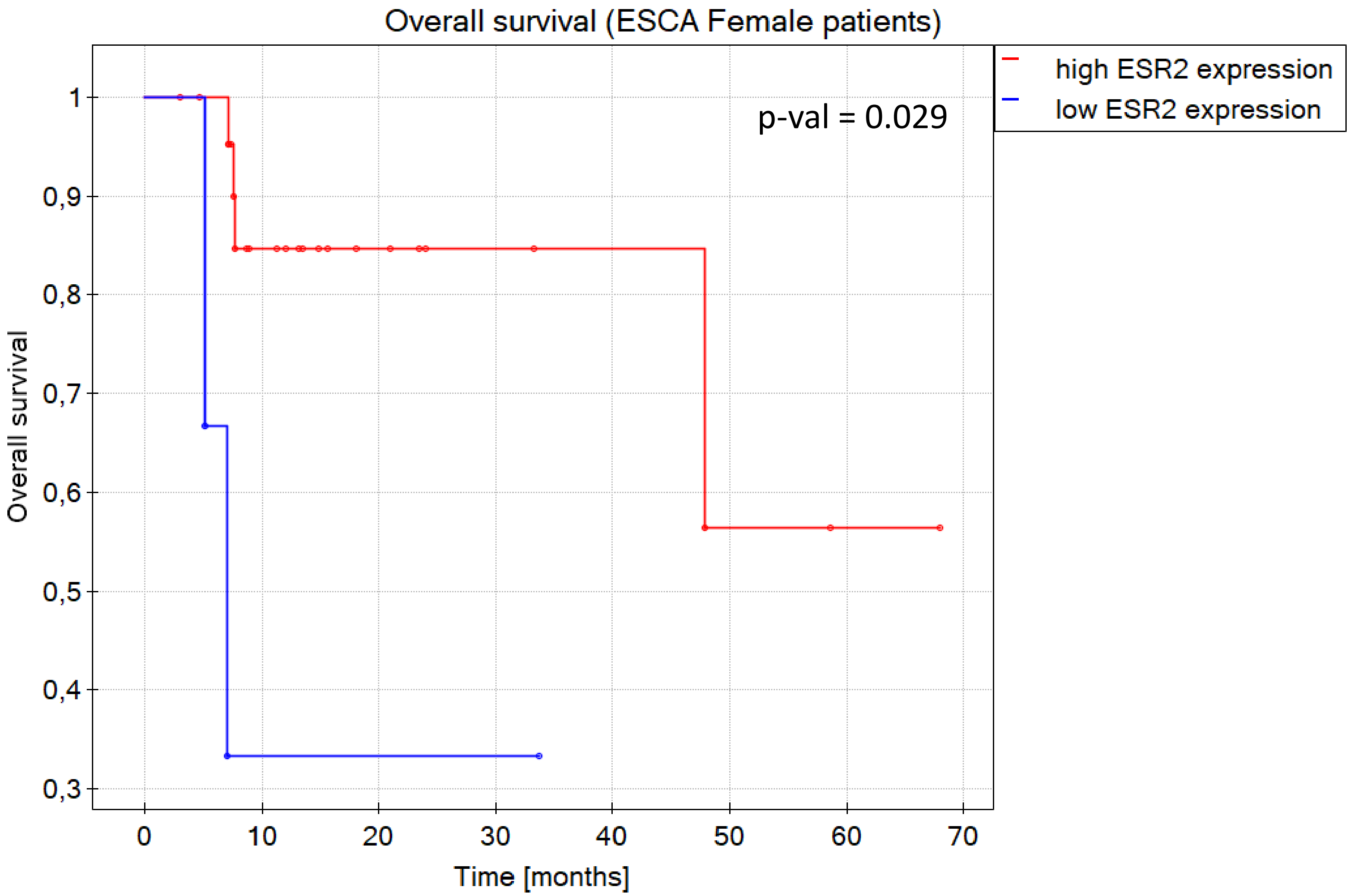

Figure S2 Kapplan-Meier plots of overall survival for TCGA cancer types with *ESR2* expression level as a factor. (a) KIRC, (b) LGG, (c) DLBC, (d) PAAD, (e) BLCA, (f) ACC, (g) ESCA, (h) HNSC, (i) LUAD, (j) KIRP, (k) STAD, (l) THCA, (m) LAML, (n) OV, (o) CESC. p-value <0.05, FDR <0.05

FigS2h

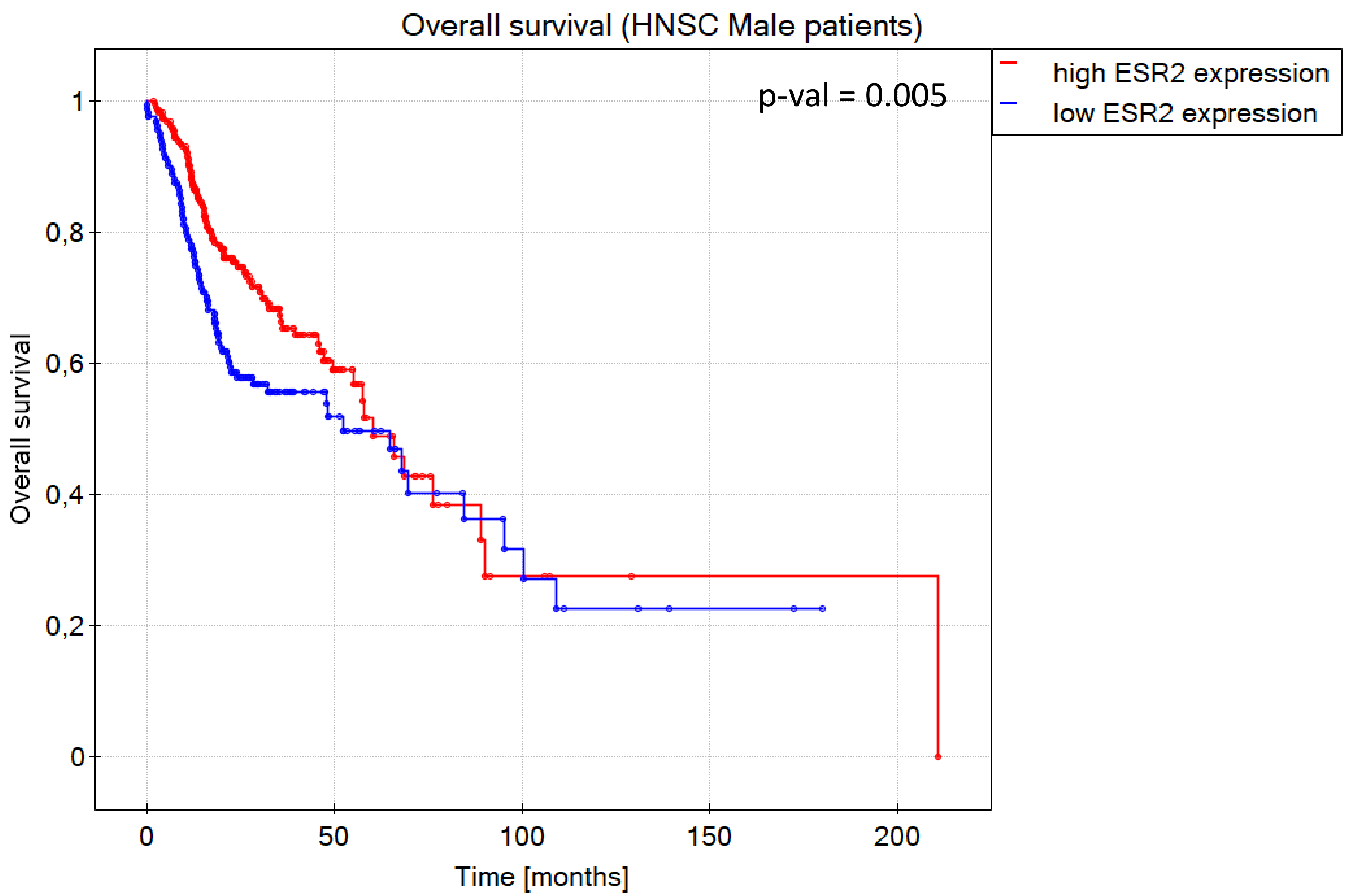

Figure S2 Kapplan-Meier plots of overall survival for TCGA cancer types with *ESR2* expression level as a factor. (a) KIRC, (b) LGG, (c) DLBC, (d) PAAD, (e) BLCA, (f) ACC, (g) ESCA, (h) HNSC, (i) LUAD, (j) KIRP, (k) STAD, (l) THCA, (m) LAML, (n) OV, (o) CESC. p-value <0.05, FDR <0.05

FigS2i

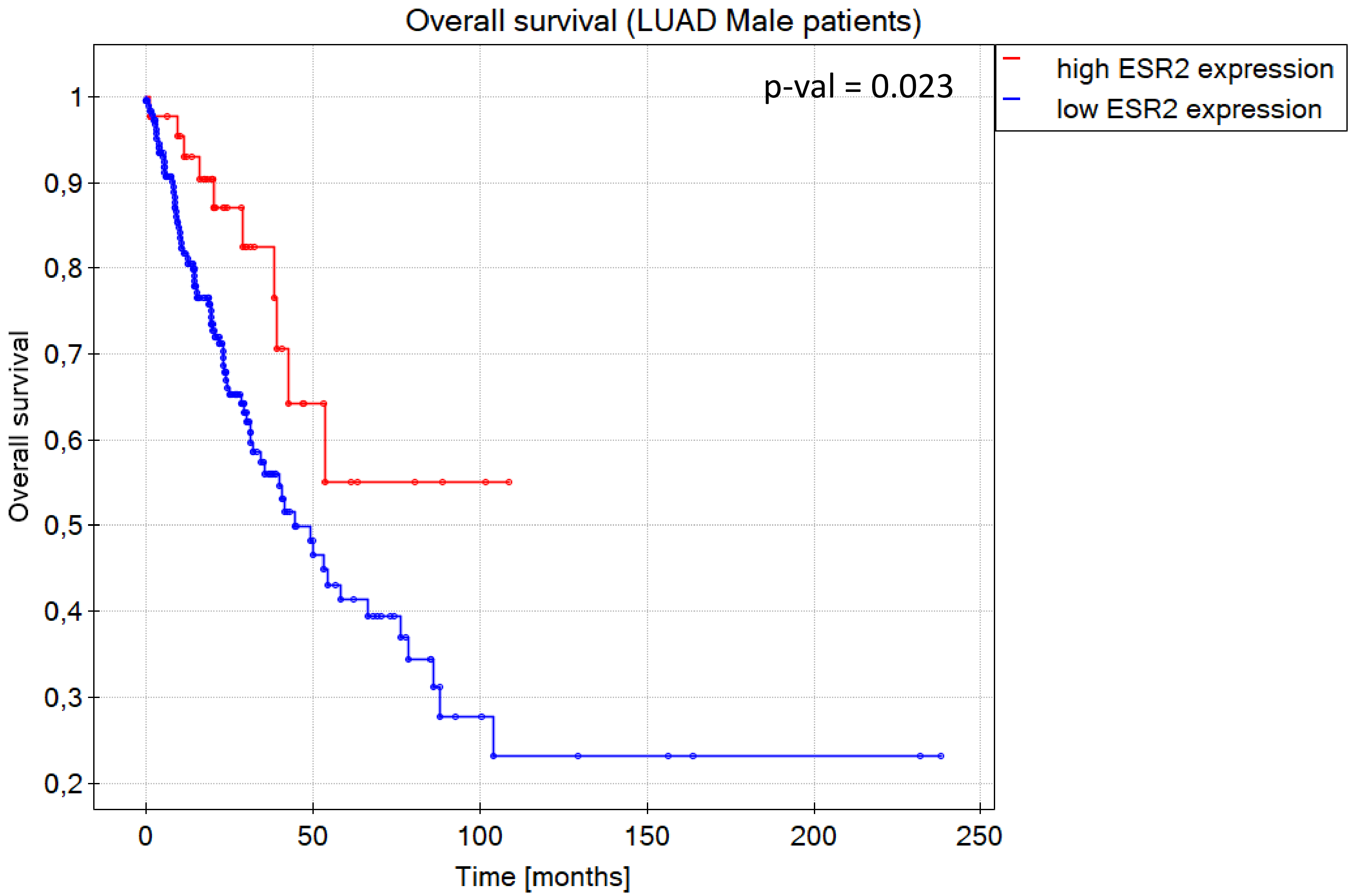

Figure S2 Kapplan-Meier plots of overall survival for TCGA cancer types with *ESR2* expression level as a factor. (a) KIRC, (b) LGG, (c) DLBC, (d) PAAD, (e) BLCA, (f) ACC, (g) ESCA, (h) HNSC, (i) LUAD, (j) KIRP, (k) STAD, (l) THCA, (m) LAML, (n) OV, (o) CESC. p-value <0.05, FDR <0.05

FigS2j

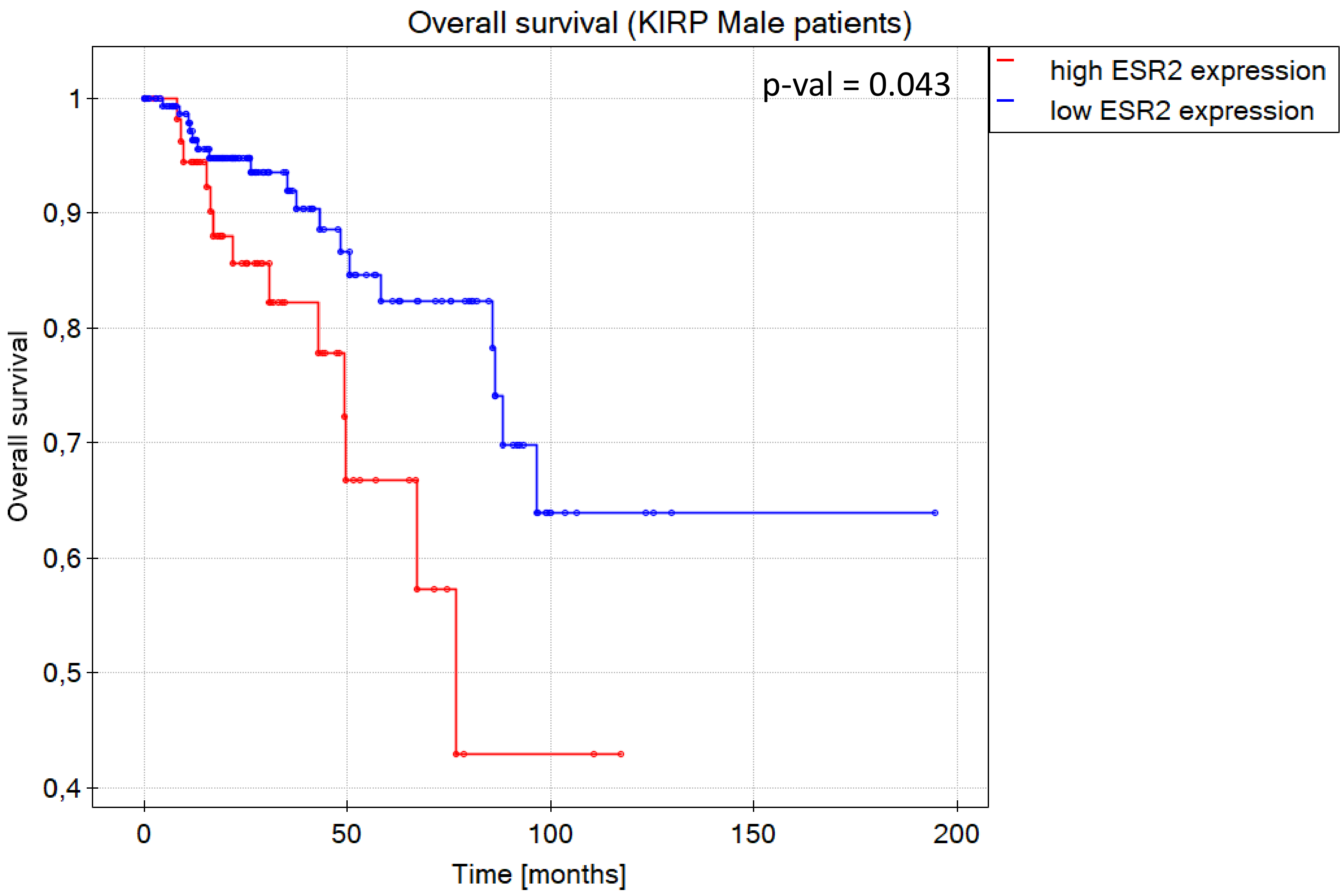

Figure S2 Kapplan-Meier plots of overall survival for TCGA cancer types with *ESR2* expression level as a factor. (a) KIRC, (b) LGG, (c) DLBC, (d) PAAD, (e) BLCA, (f) ACC, (g) ESCA, (h) HNSC, (i) LUAD, (j) KIRP, (k) STAD, (l) THCA, (m) LAML, (n) OV, (o) CESC. p-value <0.05, FDR <0.05

FigS2k

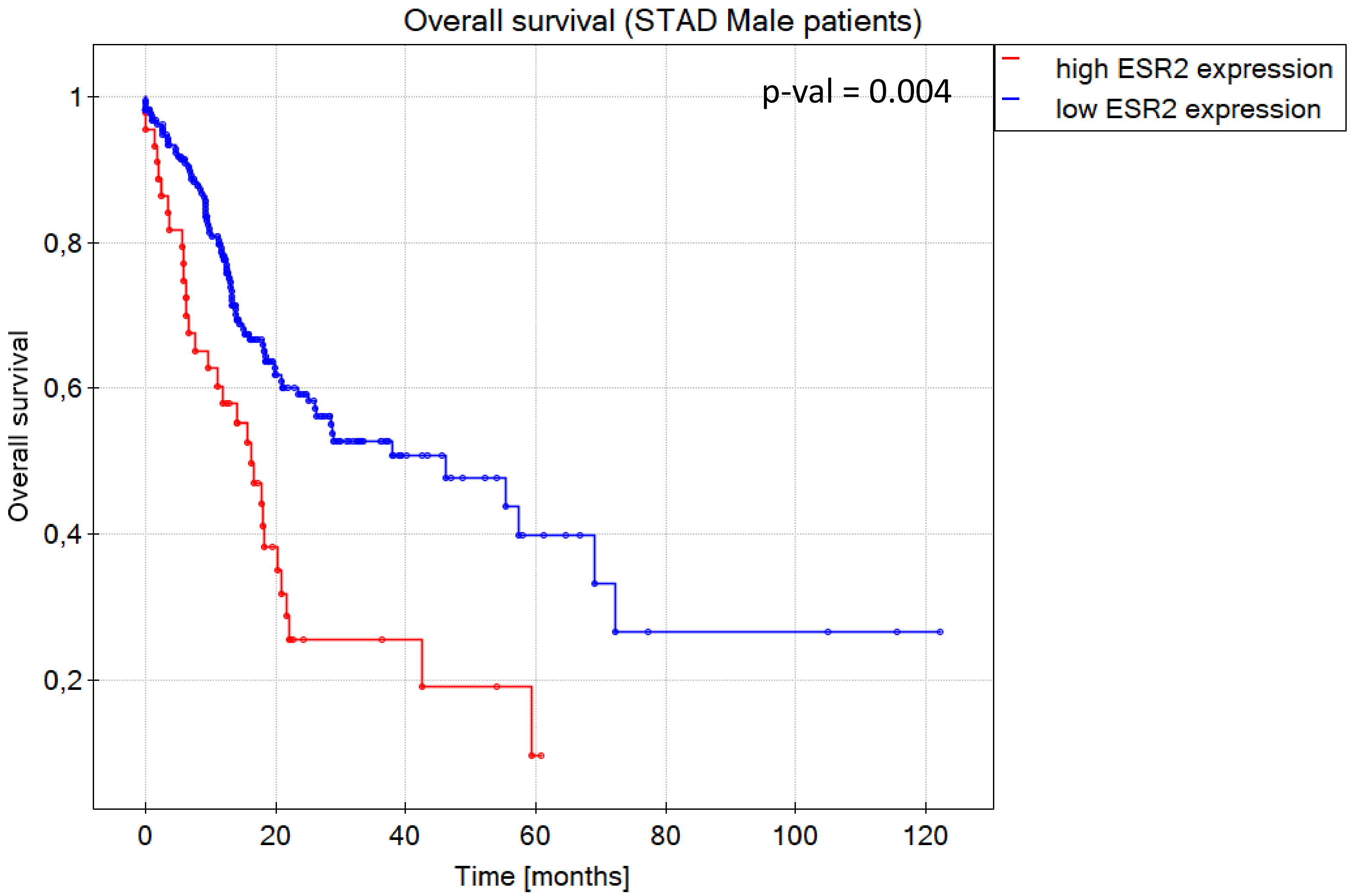

Figure S2 Kapplan-Meier plots of overall survival for TCGA cancer types with *ESR2* expression level as a factor. (a) KIRC, (b) LGG, (c) DLBC, (d) PAAD, (e) BLCA, (f) ACC, (g) ESCA, (h) HNSC, (i) LUAD, (j) KIRP, (k) STAD, (l) THCA, (m) LAML, (n) OV, (o) CESC. p-value <0.05, FDR <0.05

FigS2l

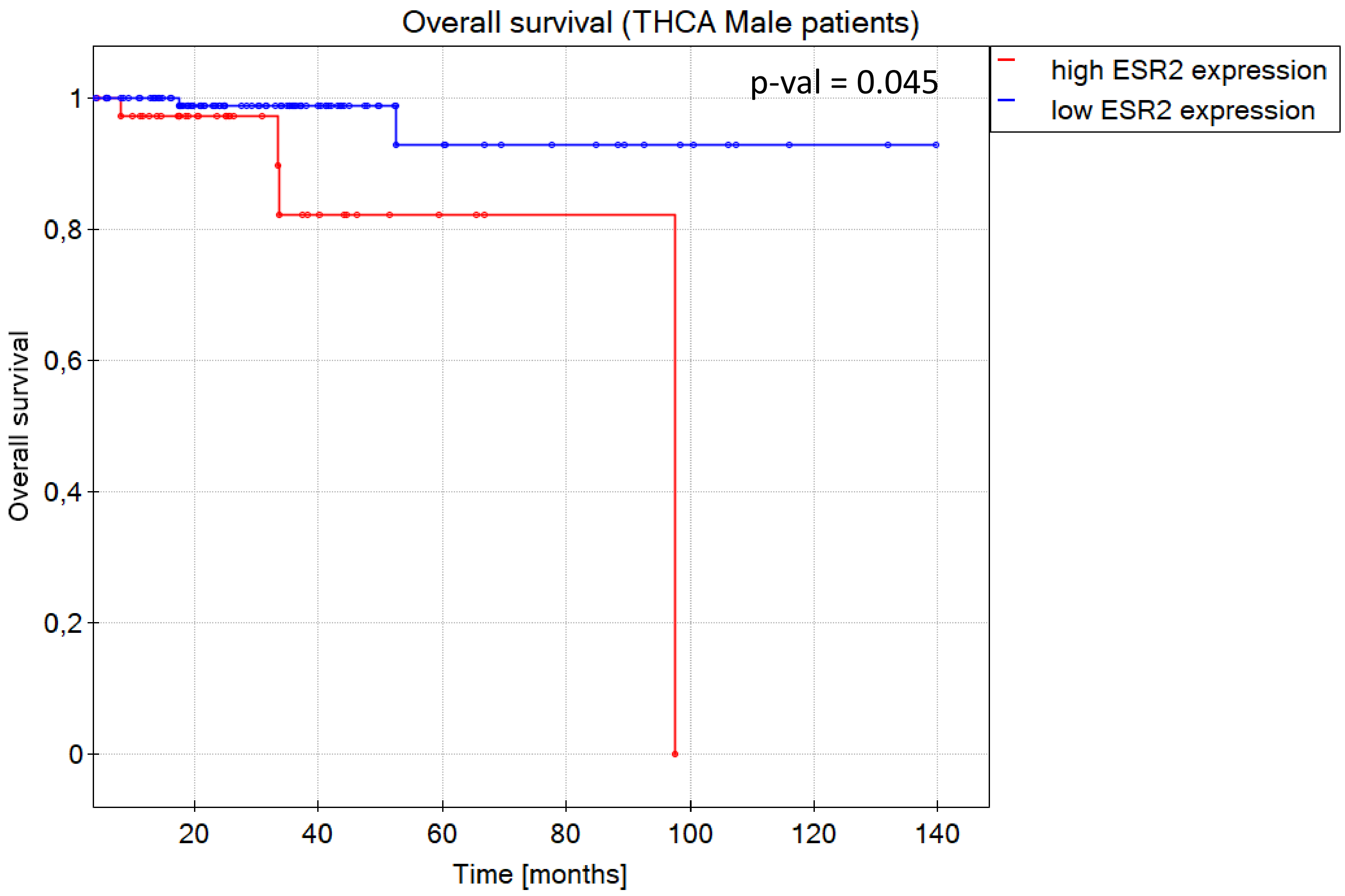

Figure S2 Kapplan-Meier plots of overall survival for TCGA cancer types with *ESR2* expression level as a factor. (a) KIRC, (b) LGG, (c) DLBC, (d) PAAD, (e) BLCA, (f) ACC, (g) ESCA, (h) HNSC, (i) LUAD, (j) KIRP, (k) STAD, (l) THCA, (m) LAML, (n) OV, (o) CESC. p-value <0.05, FDR <0.05

FigS2m

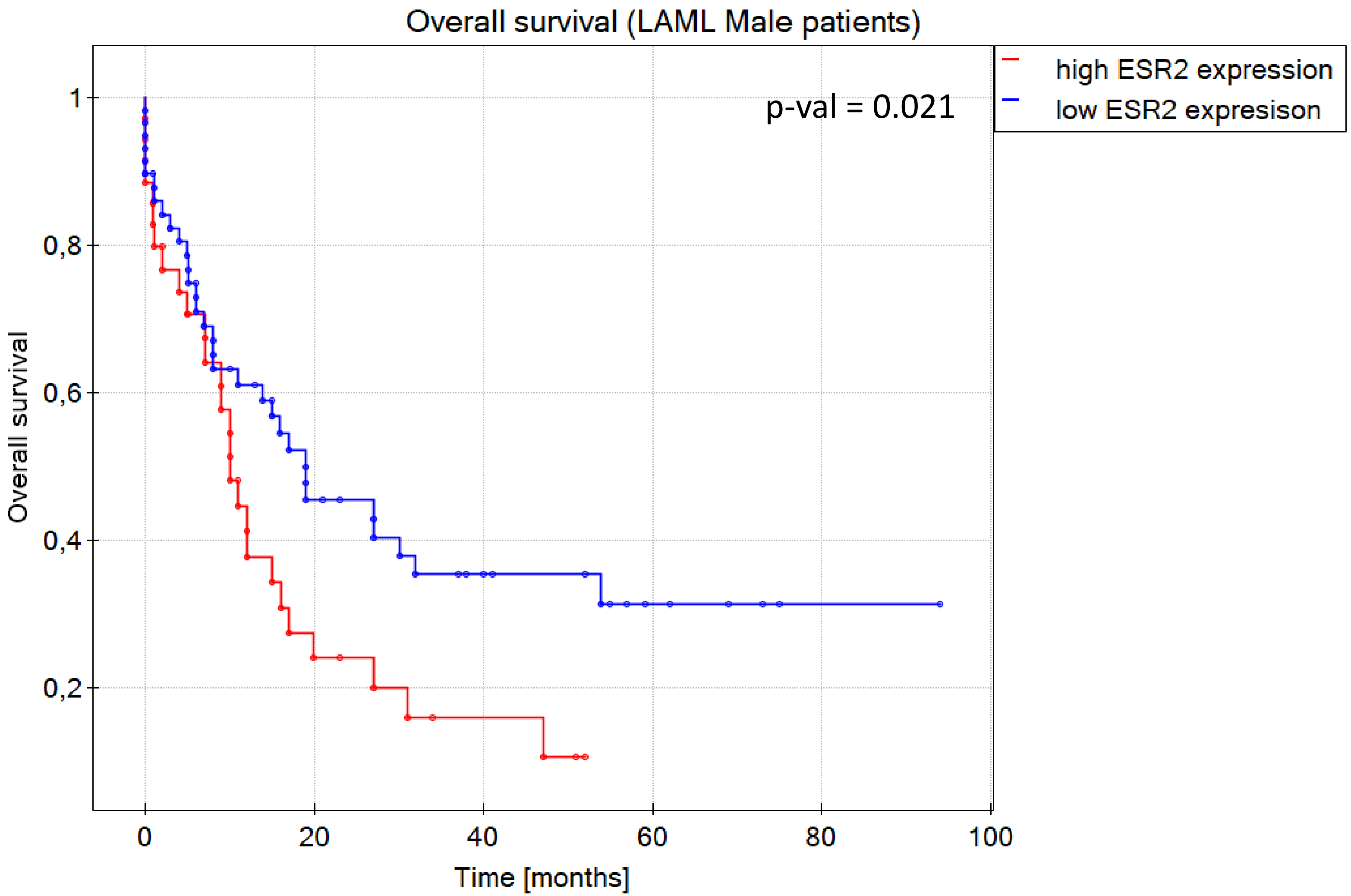

Figure S2 Kapplan-Meier plots of overall survival for TCGA cancer types with *ESR2* expression level as a factor. (a) KIRC, (b) LGG, (c) DLBC, (d) PAAD, (e) BLCA, (f) ACC, (g) ESCA, (h) HNSC, (i) LUAD, (j) KIRP, (k) STAD, (l) THCA, (m) LAML, (n) OV, (o) CESC. p-value <0.05, FDR <0.05

FigS2n

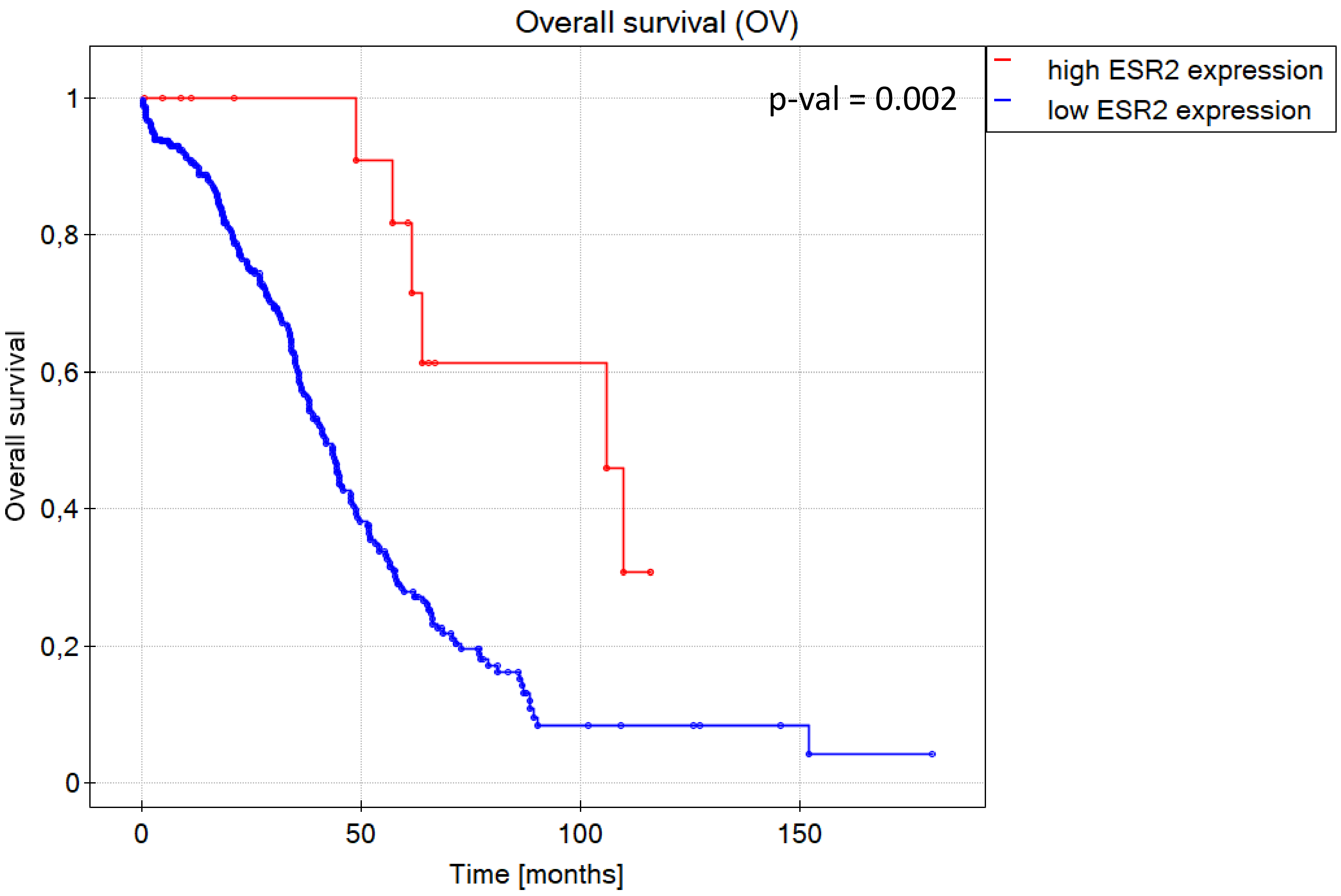

Figure S2 Kapplan-Meier plots of overall survival for TCGA cancer types with *ESR2* expression level as a factor. (a) KIRC, (b) LGG, (c) DLBC, (d) PAAD, (e) BLCA, (f) ACC, (g) ESCA, (h) HNSC, (i) LUAD, (j) KIRP, (k) STAD, (l) THCA, (m) LAML, (n) OV, (o) CESC. p-value <0.05, FDR <0.05

FigS2o

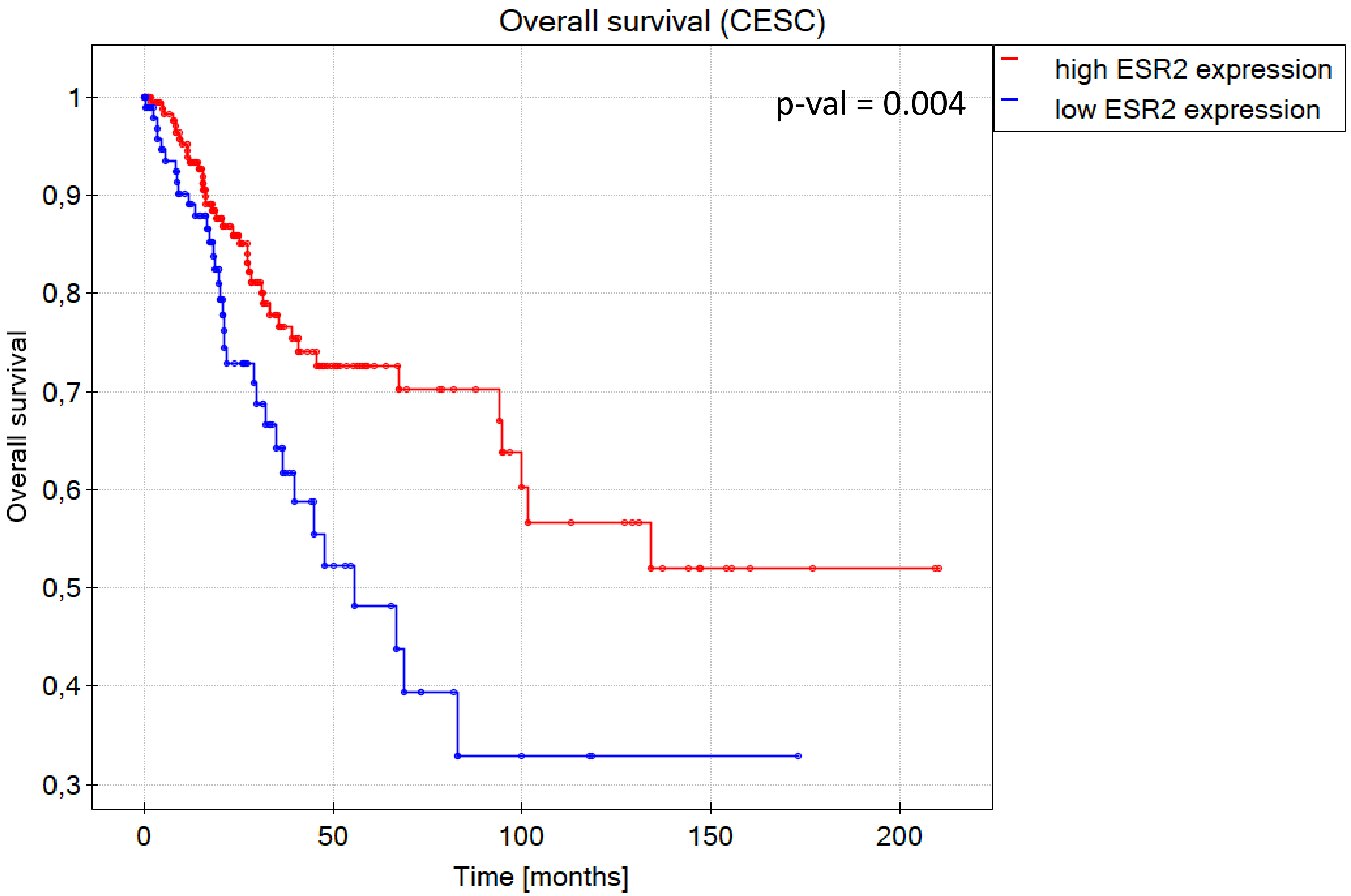

Figure S2 Kapplan-Meier plots of overall survival for TCGA cancer types with *ESR2* expression level as a factor. (a) KIRC, (b) LGG, (c) DLBC, (d) PAAD, (e) BLCA, (f) ACC, (g) ESCA, (h) HNSC, (i) LUAD, (j) KIRP, (k) STAD, (l) THCA, (m) LAML, (n) OV, (o) CESC. p-value <0.05, FDR <0.05

FigS3a

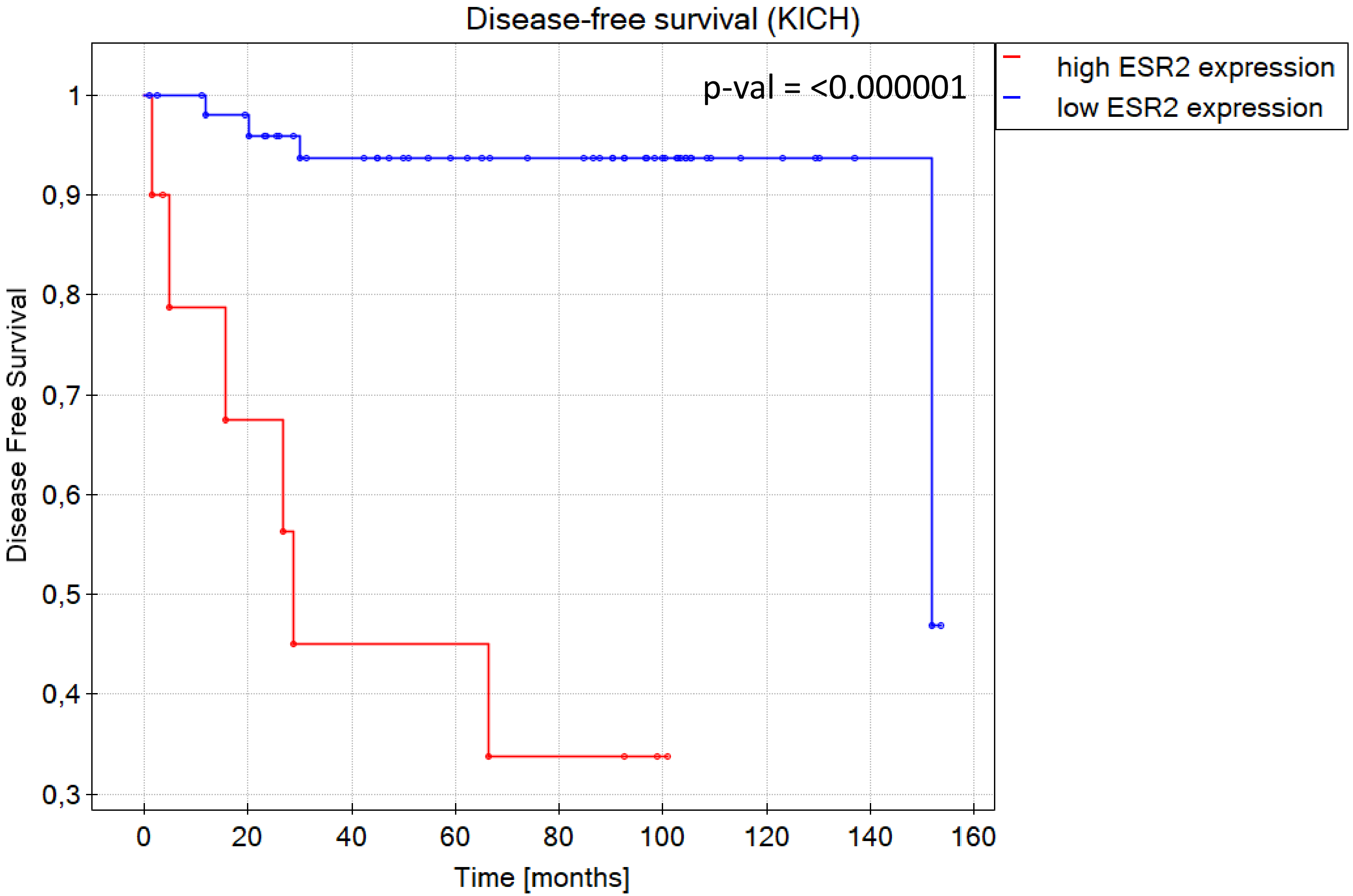

Figure S3 Kapplan-Meier plots of disease-free survival for TCGA cancer types with *ESR2* expression level as a factor. (a) KICH, (b) GBM, (c) TGCT, (d) STAD, (e) PRAD, (f) DLBC, (g) ACC. p-value <0.05, FDR <0.05

FigS3b

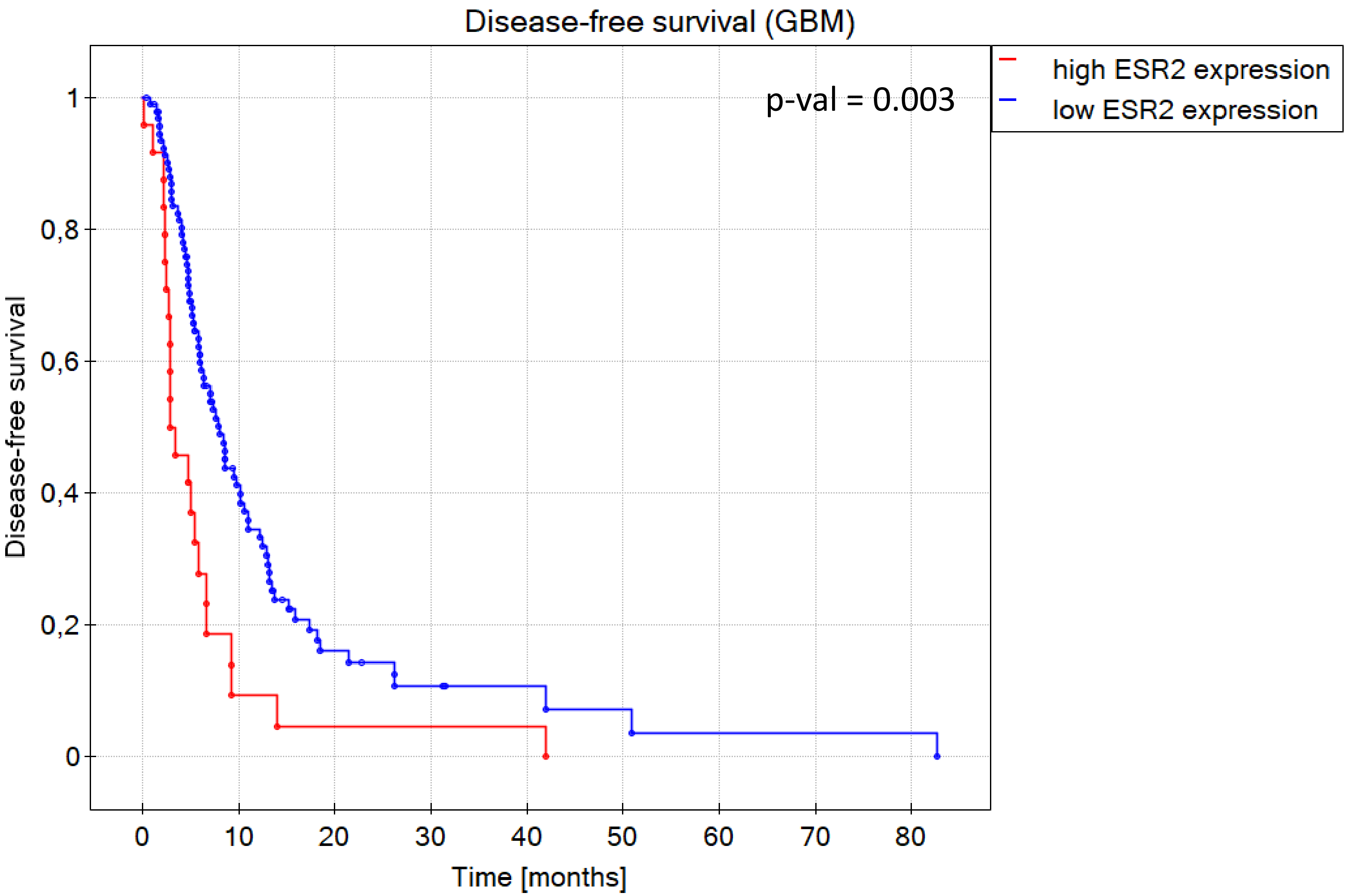

Figure S3 Kapplan-Meier plots of disease-free survival for TCGA cancer types with *ESR2* expression level as a factor. (a) KICH, (b) GBM, (c) TGCT, (d) STAD, (e) PRAD, (f) DLBC, (g) ACC. p-value <0.05, FDR <0.05

FigS3c

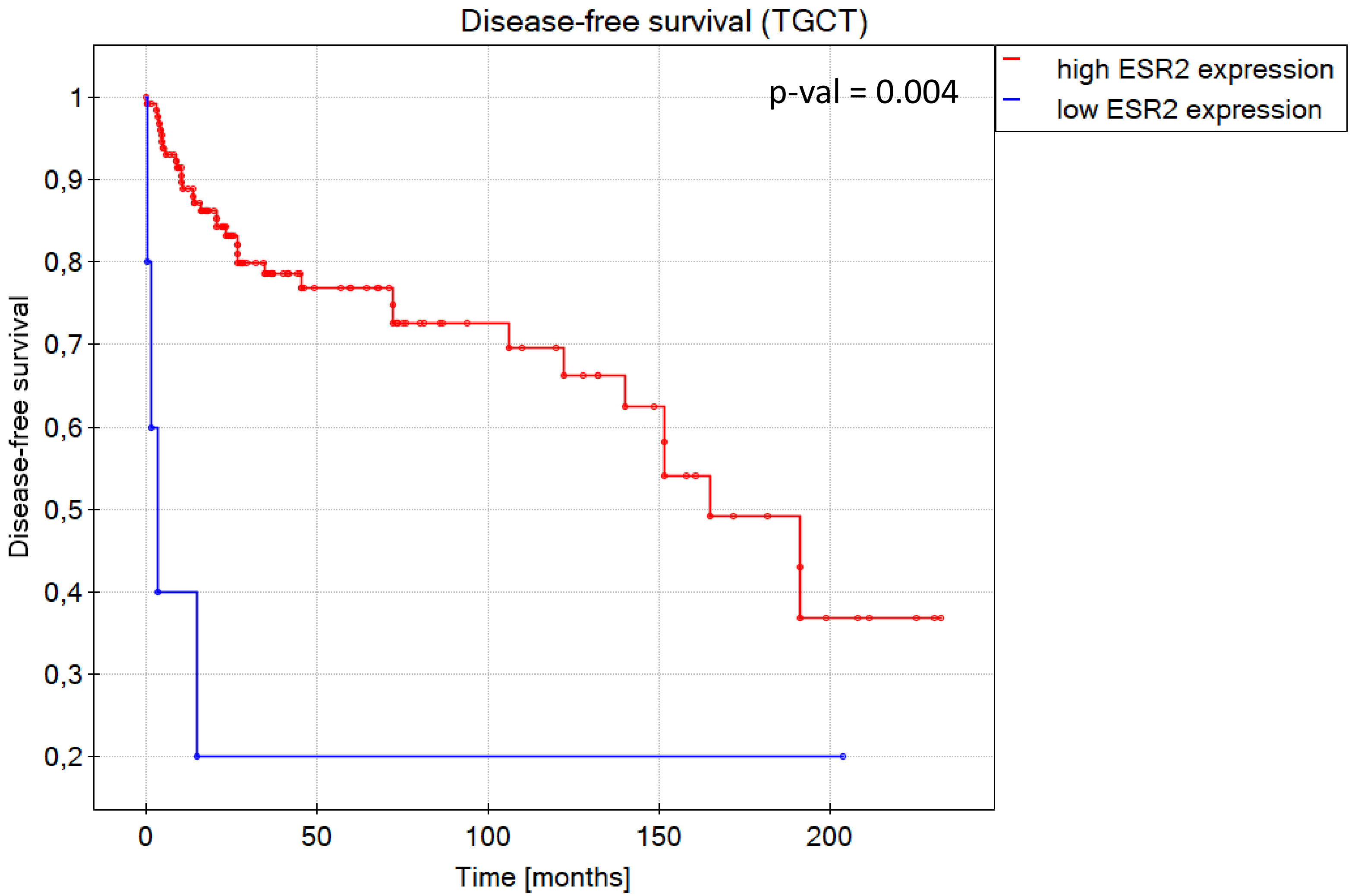

Figure S3 Kapplan-Meier plots of disease-free survival for TCGA cancer types with *ESR2* expression level as a factor. (a) KICH, (b) GBM, (c) TGCT, (d) STAD, (e) PRAD, (f) DLBC, (g) ACC. p-value <0.05, FDR <0.05

FigS3d

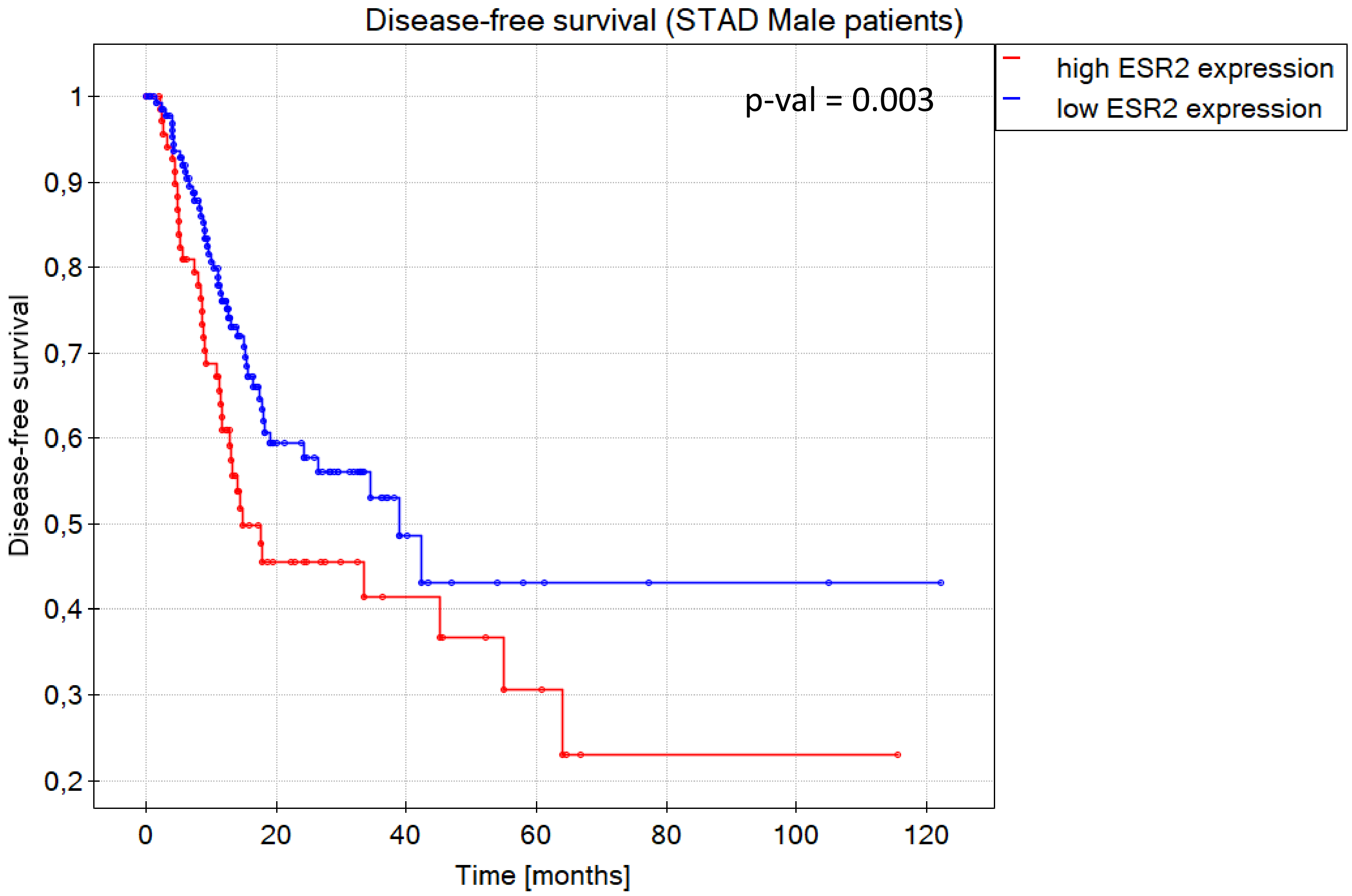

Figure S3 Kapplan-Meier plots of disease-free survival for TCGA cancer types with *ESR2* expression level as a factor. (a) KICH, (b) GBM, (c) TGCT, (d) STAD, (e) PRAD, (f) DLBC, (g) ACC. p-value <0.05, FDR <0.05

FigS3e

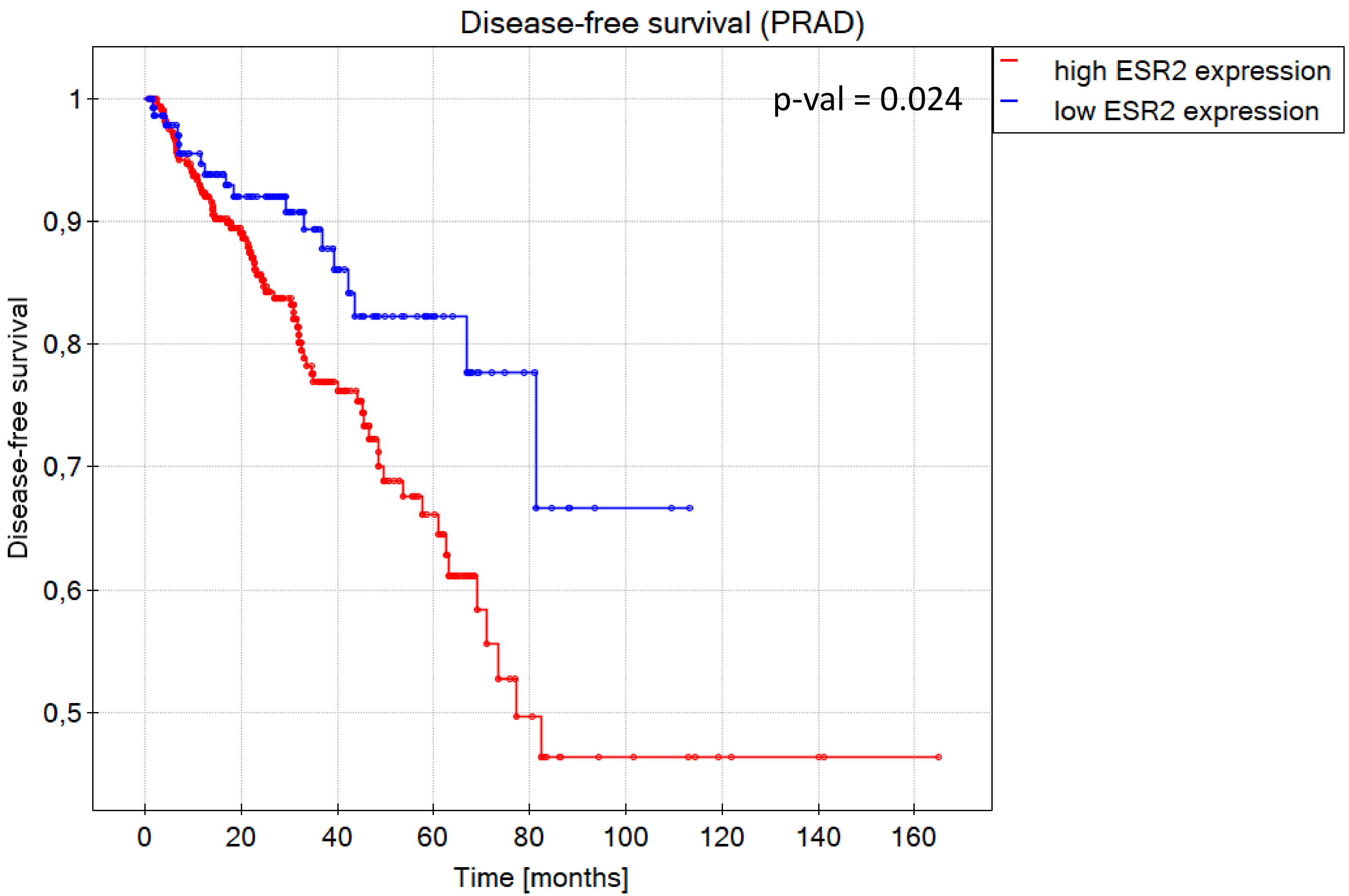

Figure S3 Kapplan-Meier plots of disease-free survival for TCGA cancer types with *ESR2* expression level as a factor. (a) KICH, (b) GBM, (c) TGCT, (d) STAD, (e) PRAD, (f) DLBC, (g) ACC. p-value <0.05, FDR <0.05

FigS3f

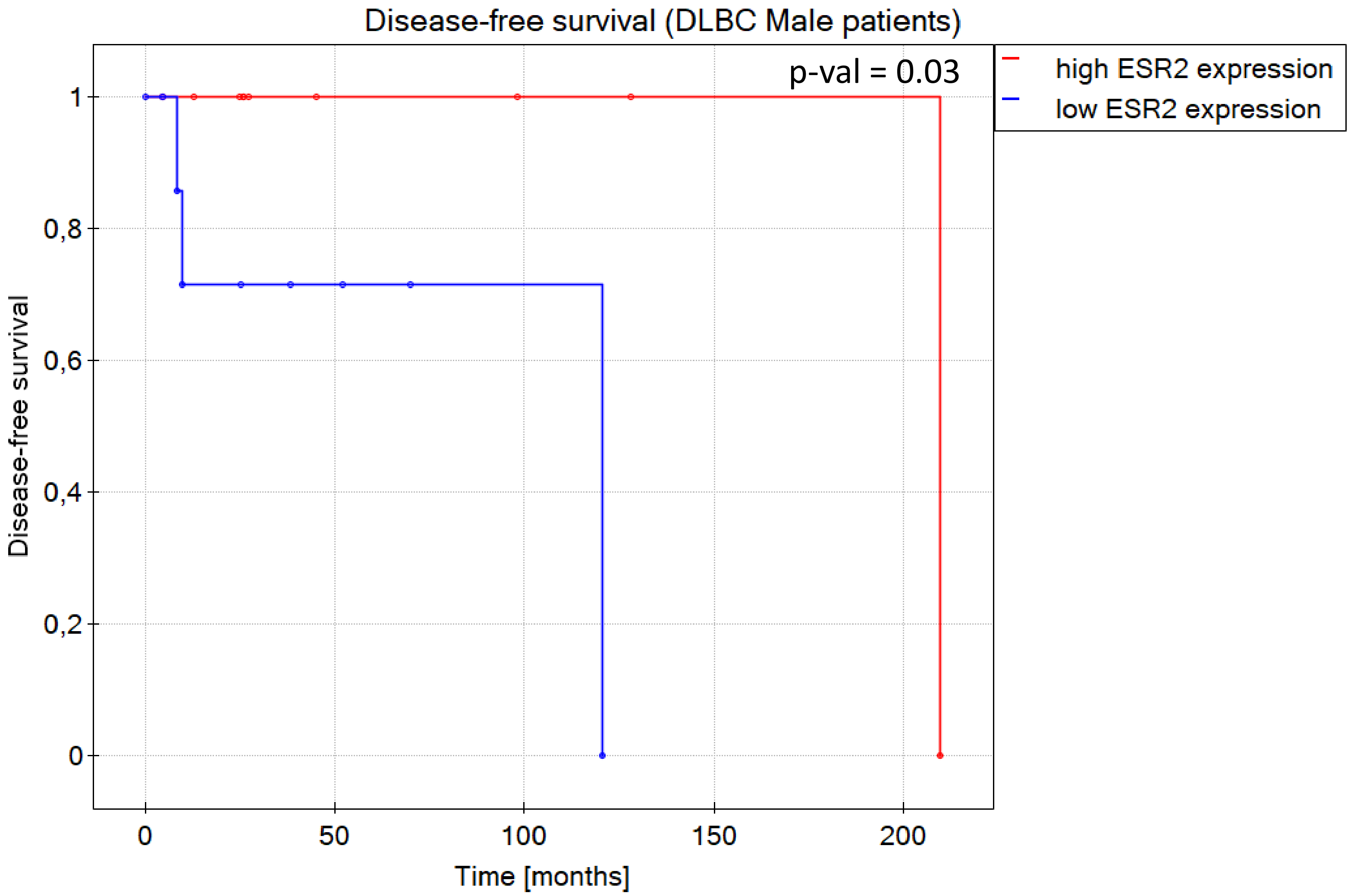

Figure S3 Kapplan-Meier plots of disease-free survival for TCGA cancer types with *ESR2* expression level as a factor. (a) KICH, (b) GBM, (c) TGCT, (d) STAD, (e) PRAD, (f) DLBC, (g) ACC. p-value <0.05, FDR <0.05

FigS3g

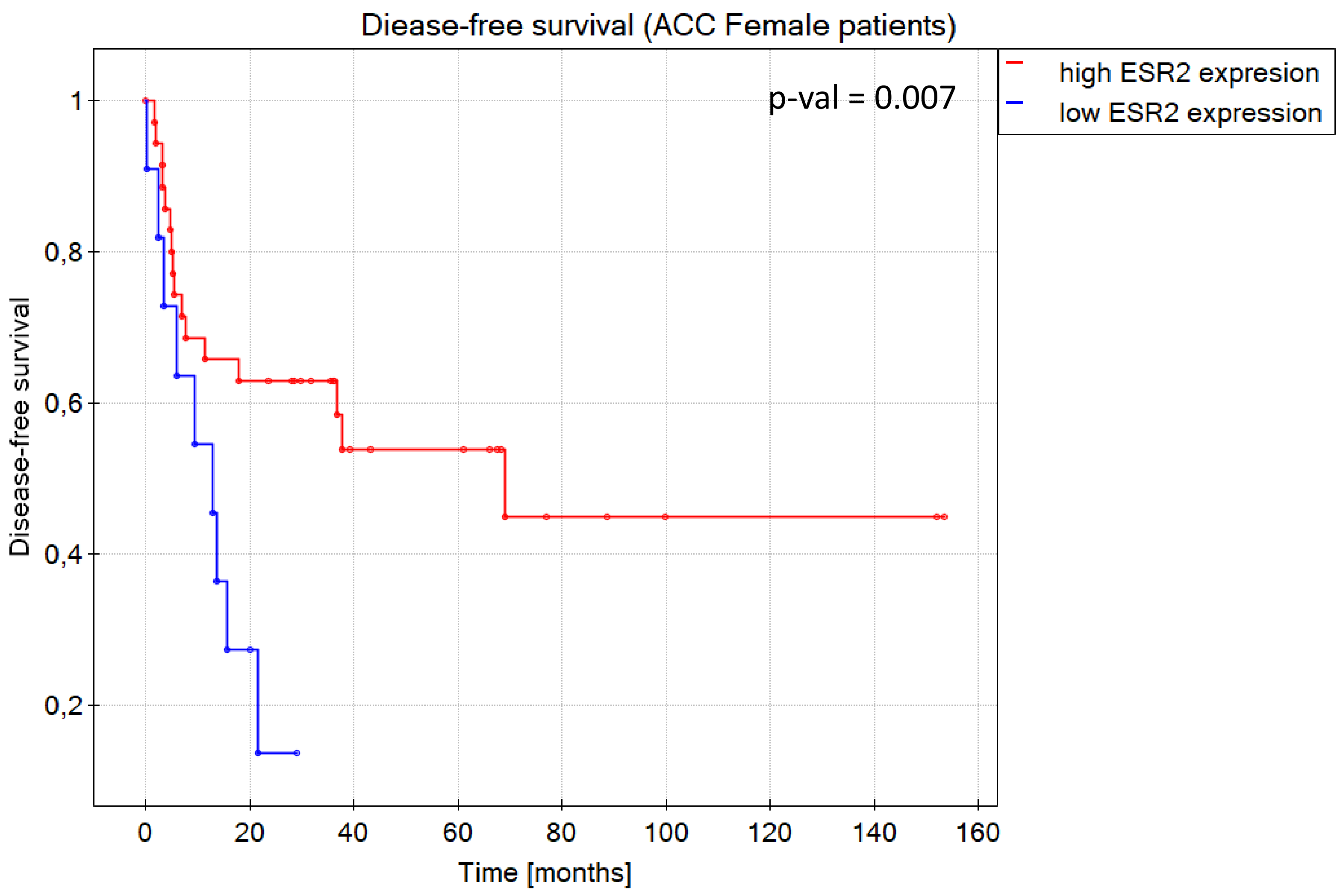

Figure S3 Kapplan-Meier plots of disease-free survival for TCGA cancer types with *ESR2* expression level as a factor. (a) KICH, (b) GBM, (c) TGCT, (d) STAD, (e) PRAD, (f) DLBC, (g) ACC. p-value <0.05, FDR <0.05

FigS4a

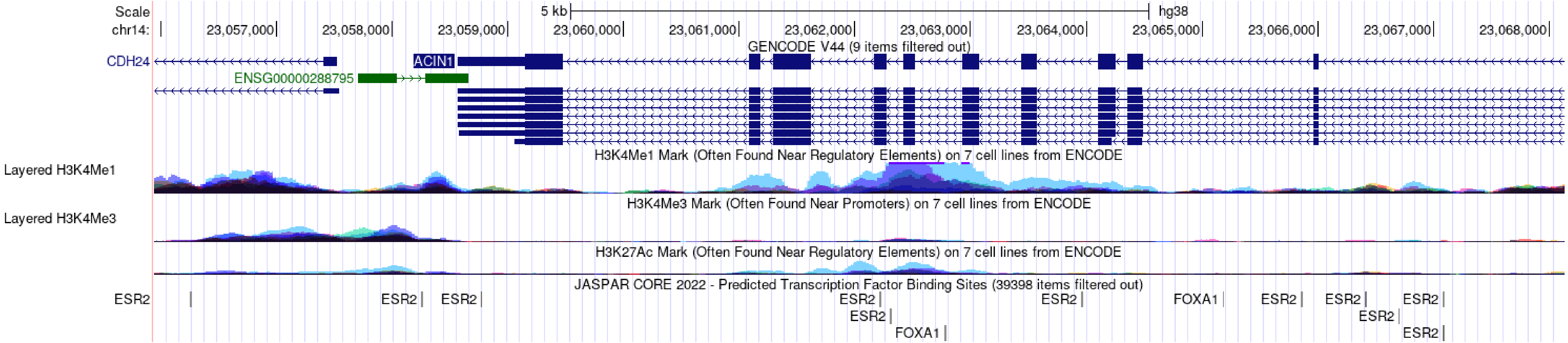

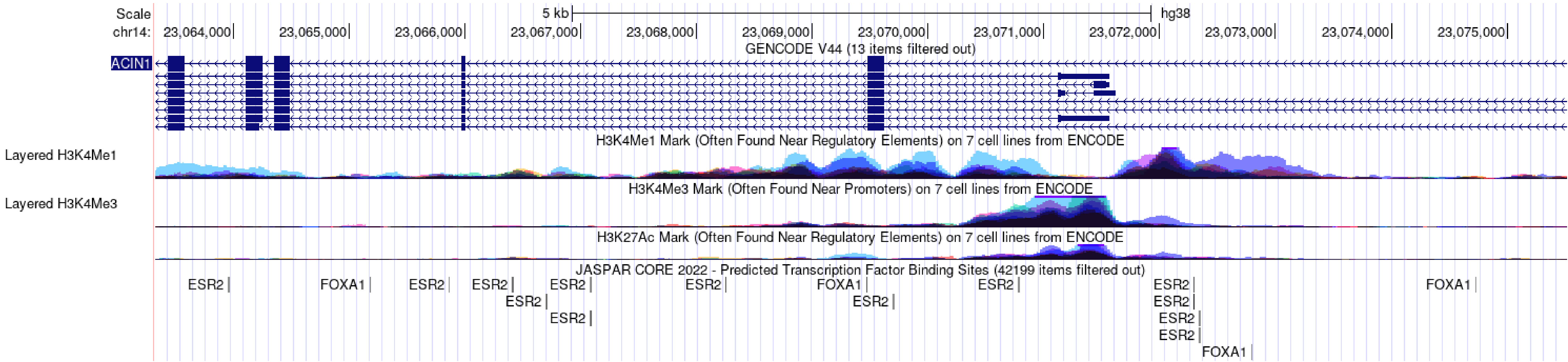

Figure S4 UCSC Genome Browser screengrab showing epigenetic marks of open chromatin and ERβ (*ESR2*) and HNF3α (*FOXA1*) binding sites in genes of interest. (a) *ACIN1*, (b) *FNBP4*, (c) *MDM4*, (d) *NDUFB3*, (e) *OCIAD2*, (f) *PLIN3*, (g) *POU2AF1*, (h) *RAC1*, (i) *SYNE2*, (j) *CFL1*, (k) *TMEM141*.

FigS4b

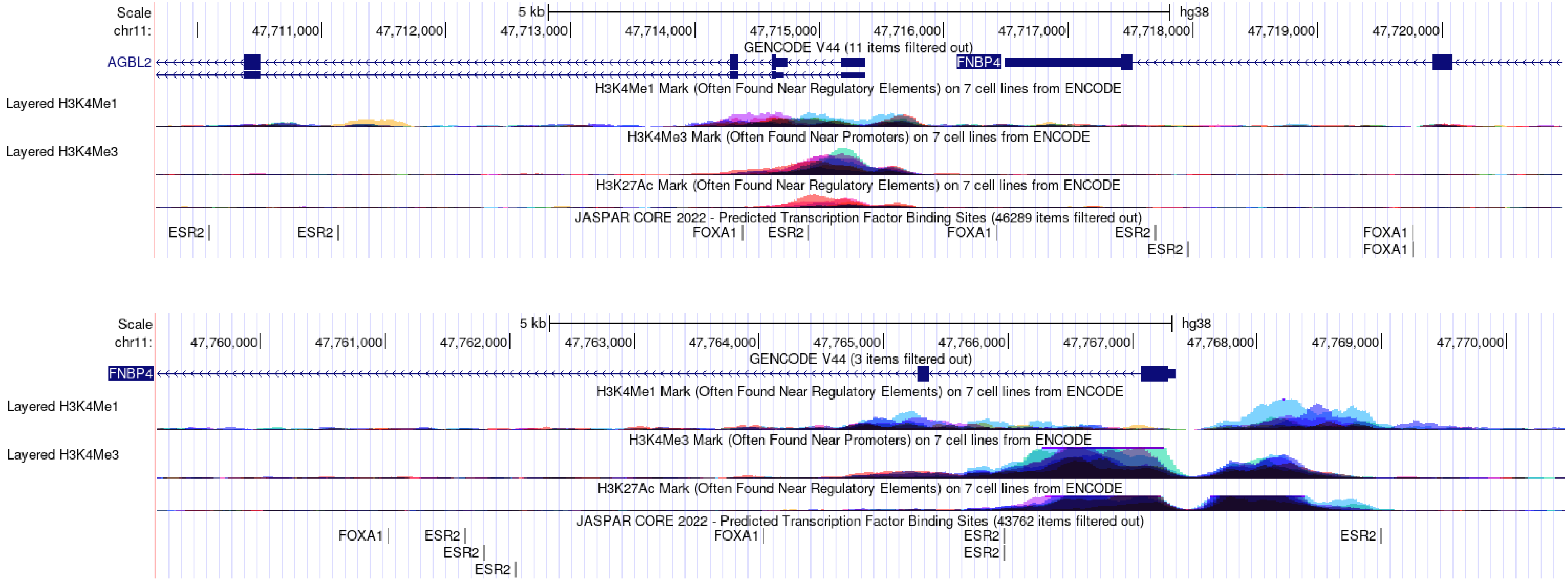

Figure S4 UCSC Genome Browser screengrab showing epigenetic marks of open chromatin and ERβ (*ESR2*) and HNF3α (*FOXA1*) binding sites in genes of interest. (a) *ACIN1*, (b) *FNBP4*, (c) *MDM4*, (d) *NDUFB3*, (e) *OCIAD2*, (f) *PLIN3*, (g) *POU2AF1*, (h) *RAC1*, (i) *SYNE2*, (j) *CFL1*, (k) *TMEM141*.

FigS4c

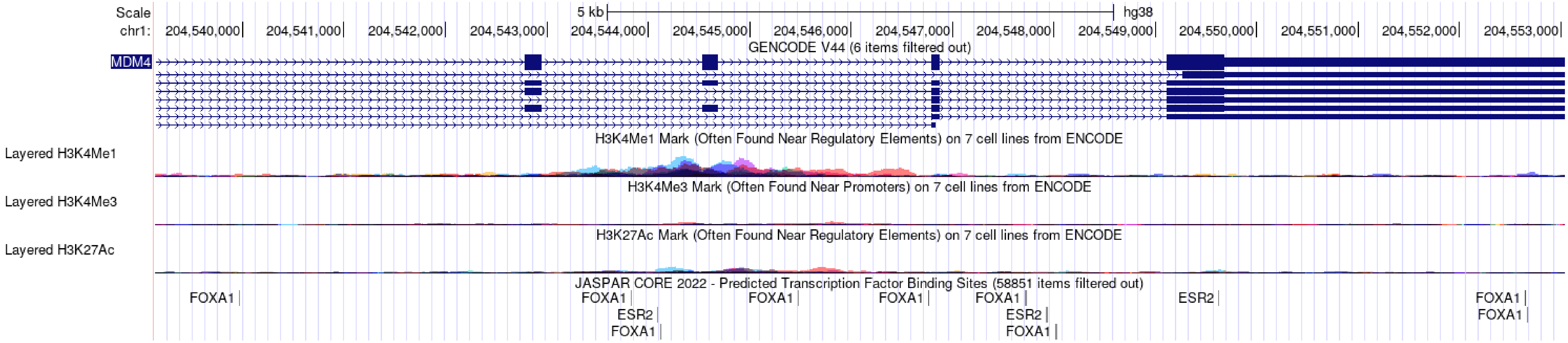

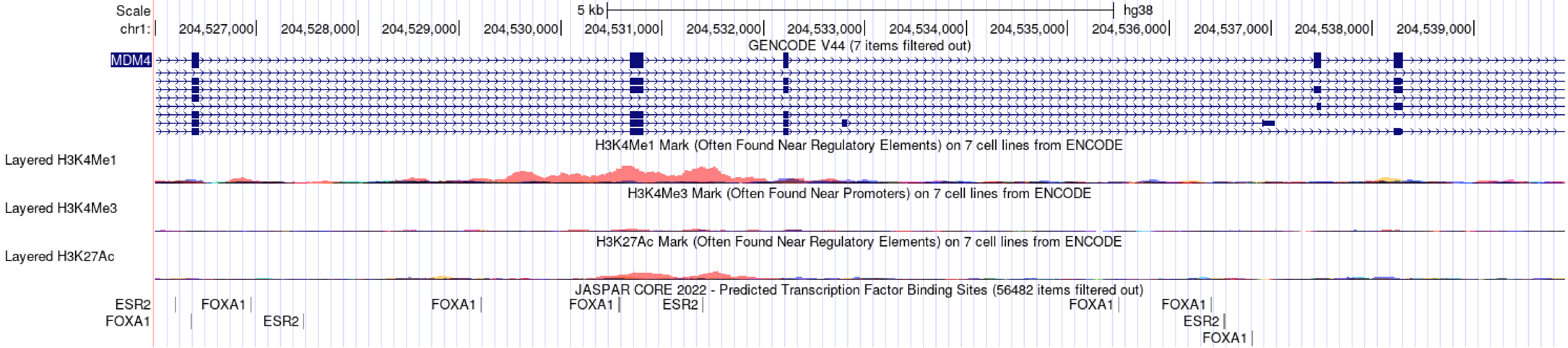

Figure S4 UCSC Genome Browser screengrab showing epigenetic marks of open chromatin and ERβ (*ESR2*) and HNF3α (*FOXA1*) binding sites in genes of interest. (a) *ACIN1*, (b) *FNBP4*, (c) *MDM4*, (d) *NDUFB3*, (e) *OCIAD2*, (f) *PLIN3*, (g) *POU2AF1*, (h) *RAC1*, (i) *SYNE2*, (j) *CFL1*, (k) *TMEM141*.

FigS4d

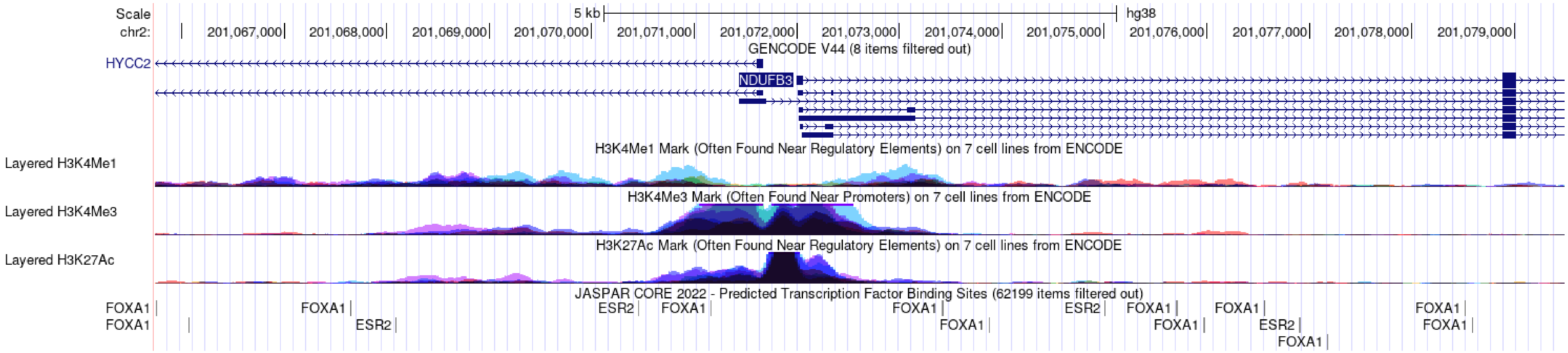

Figure S4 UCSC Genome Browser screengrab showing epigenetic marks of open chromatin and ERβ (*ESR2*) and HNF3α (*FOXA1*) binding sites in genes of interest. (a) *ACIN1*, (b) *FNBP4*, (c) *MDM4*, (d) *NDUFB3*, (e) *OCIAD2*, (f) *PLIN3*, (g) *POU2AF1*, (h) *RAC1*, (i) *SYNE2*, (j) *CFL1*, (k) *TMEM141*.

FigS4e

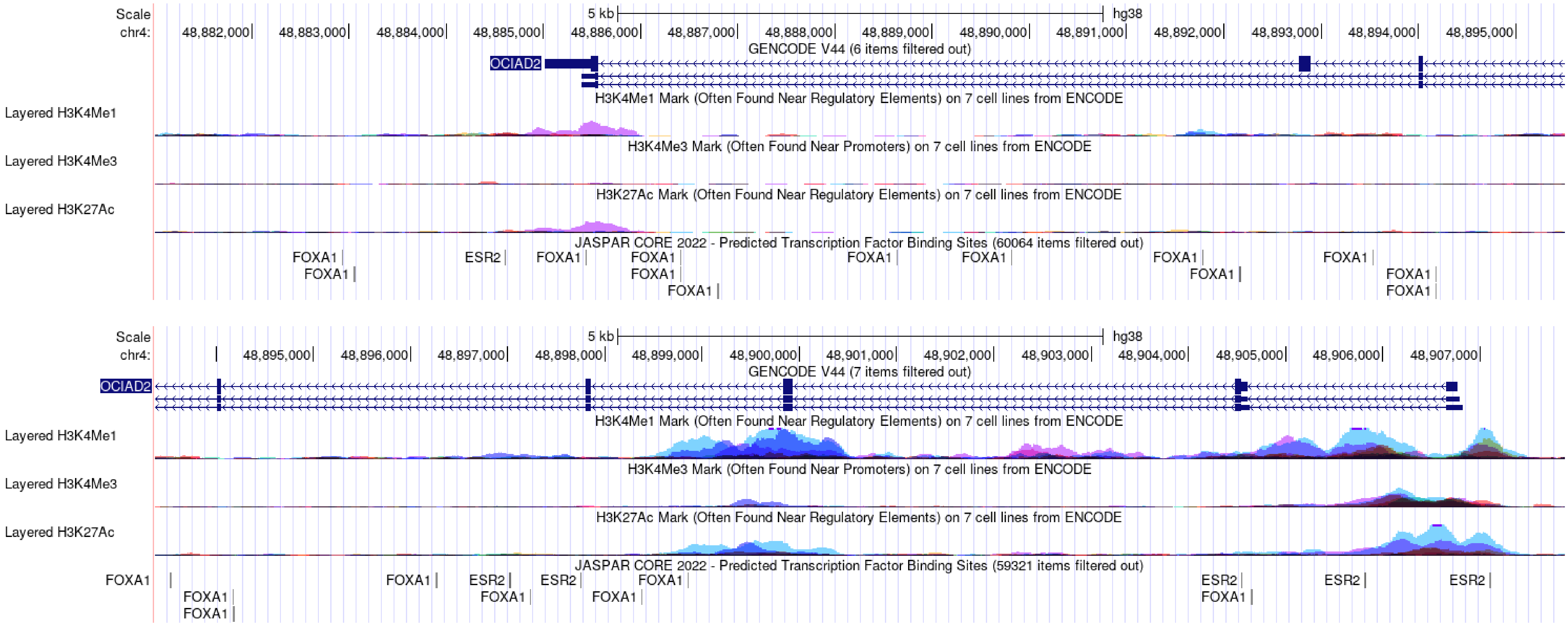

Figure S4 UCSC Genome Browser screengrab showing epigenetic marks of open chromatin and ERβ (*ESR2*) and HNF3α (*FOXA1*) binding sites in genes of interest. (a) *ACIN1*, (b) *FNBP4*, (c) *MDM4*, (d) *NDUFB3*, (e) *OCIAD2*, (f) *PLIN3*, (g) *POU2AF1*, (h) *RAC1*, (i) *SYNE2*, (j) *CFL1*, (k) *TMEM141*.

FigS4f

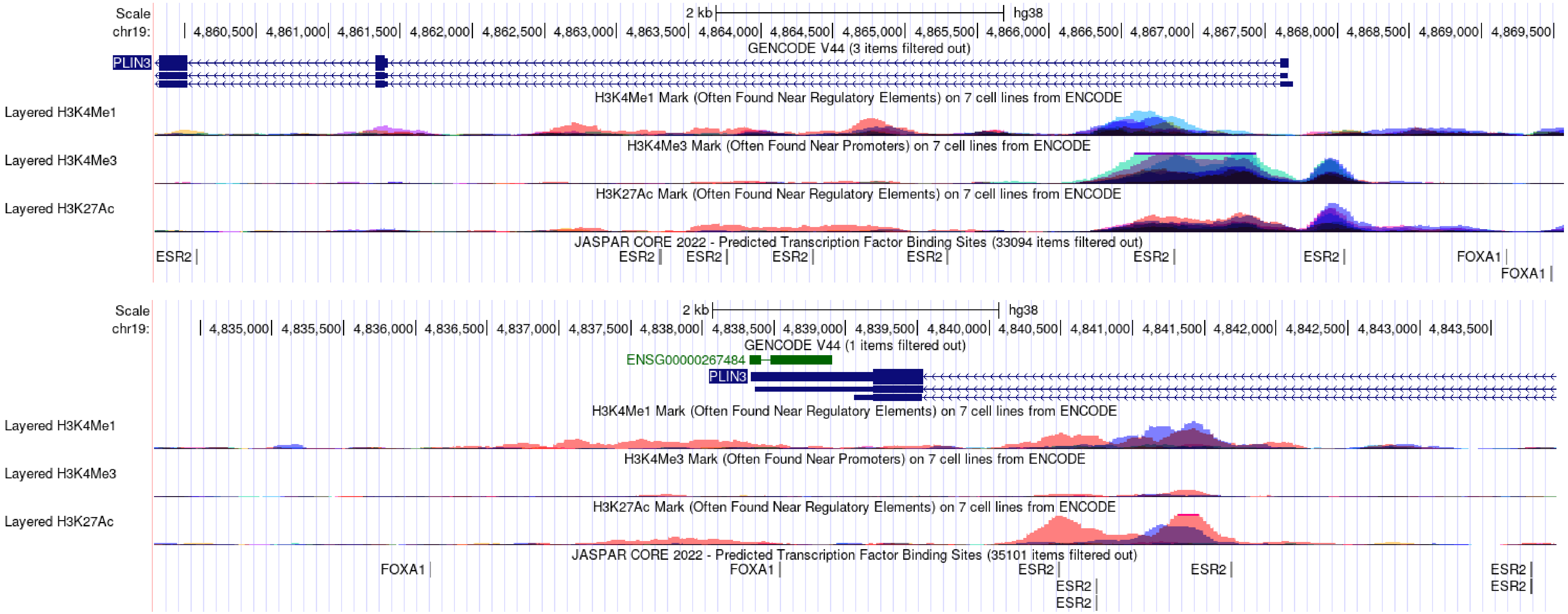

Figure S4 UCSC Genome Browser screengrab showing epigenetic marks of open chromatin and ERβ (*ESR2*) and HNF3α (*FOXA1*) binding sites in genes of interest. (a) *ACIN1*, (b) *FNBP4*, (c) *MDM4*, (d) *NDUFB3*, (e) *OCIAD2*, (f) *PLIN3*, (g) *POU2AF1*, (h) *RAC1*, (i) *SYNE2*, (j) *CFL1*, (k) *TMEM141*.

FigS4g

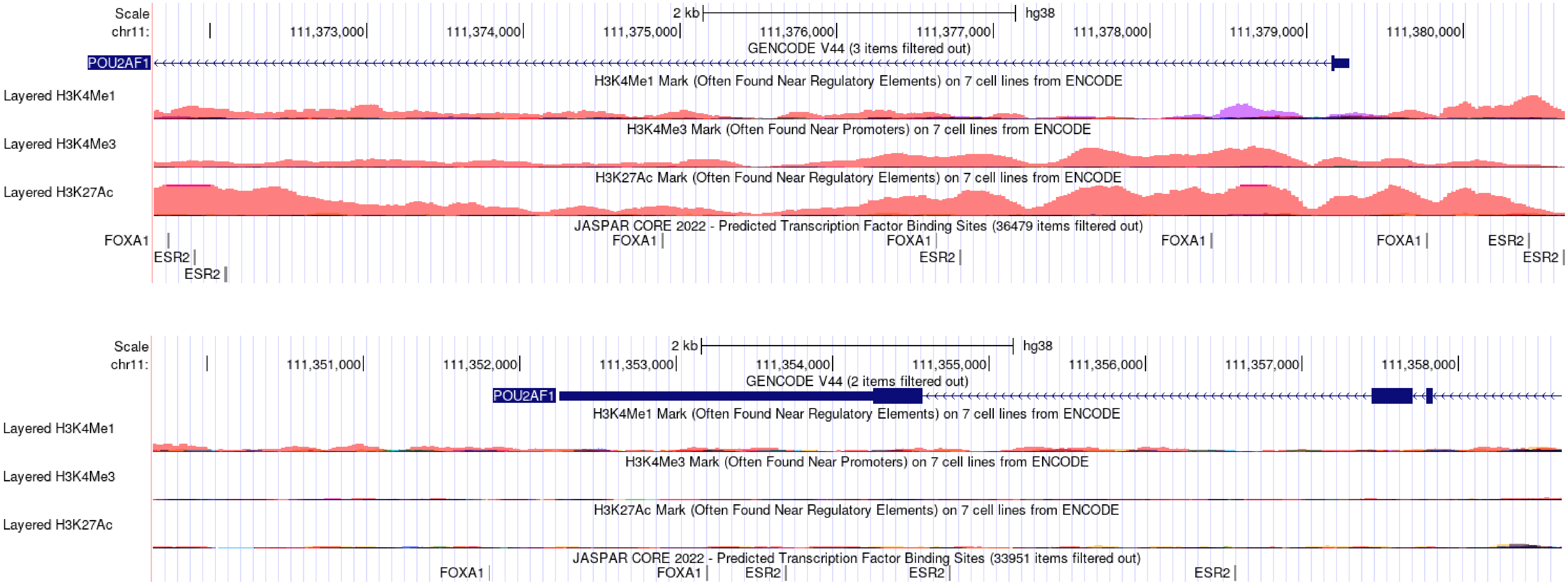

Figure S4 UCSC Genome Browser screengrab showing epigenetic marks of open chromatin and ERβ (*ESR2*) and HNF3α (*FOXA1*) binding sites in genes of interest. (a) *ACIN1*, (b) *FNBP4*, (c) *MDM4*, (d) *NDUFB3*, (e) *OCIAD2*, (f) *PLIN3*, (g) *POU2AF1*, (h) *RAC1*, (i) *SYNE2*, (j) *CFL1*, (k) *TMEM141*.

FigS4h

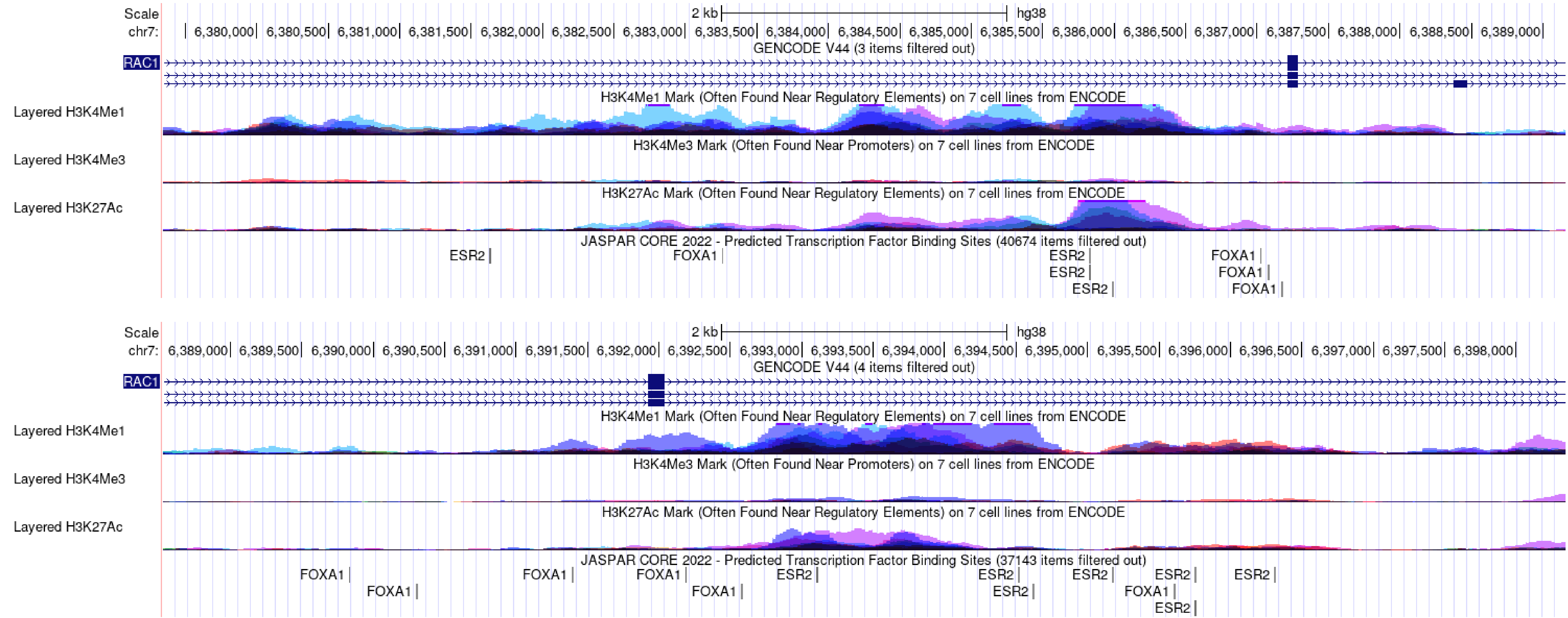

Figure S4 UCSC Genome Browser screengrab showing epigenetic marks of open chromatin and ERβ (*ESR2*) and HNF3α (*FOXA1*) binding sites in genes of interest. (a) *ACIN1*, (b) *FNBP4*, (c) *MDM4*, (d) *NDUFB3*, (e) *OCIAD2*, (f) *PLIN3*, (g) *POU2AF1*, (h) *RAC1*, (i) *SYNE2*, (j) *CFL1*, (k) *TMEM141*.

FigS4i

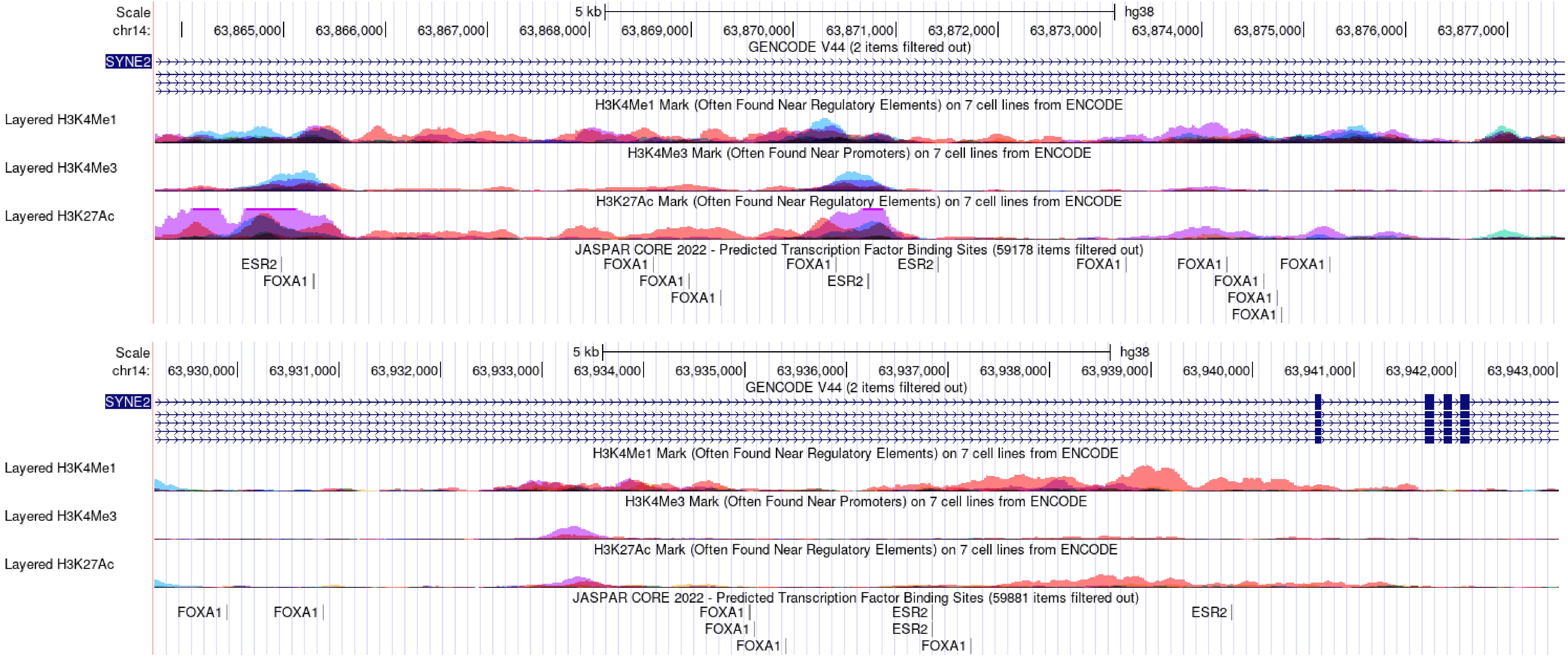

Figure S4 UCSC Genome Browser screengrab showing epigenetic marks of open chromatin and ERβ (*ESR2*) and HNF3α (*FOXA1*) binding sites in genes of interest. (a) *ACIN1*, (b) *FNBP4*, (c) *MDM4*, (d) *NDUFB3*, (e) *OCIAD2*, (f) *PLIN3*, (g) *POU2AF1*, (h) *RAC1*, (i) *SYNE2*, (j) *CFL1*, (k) *TMEM141*.

FigS4j

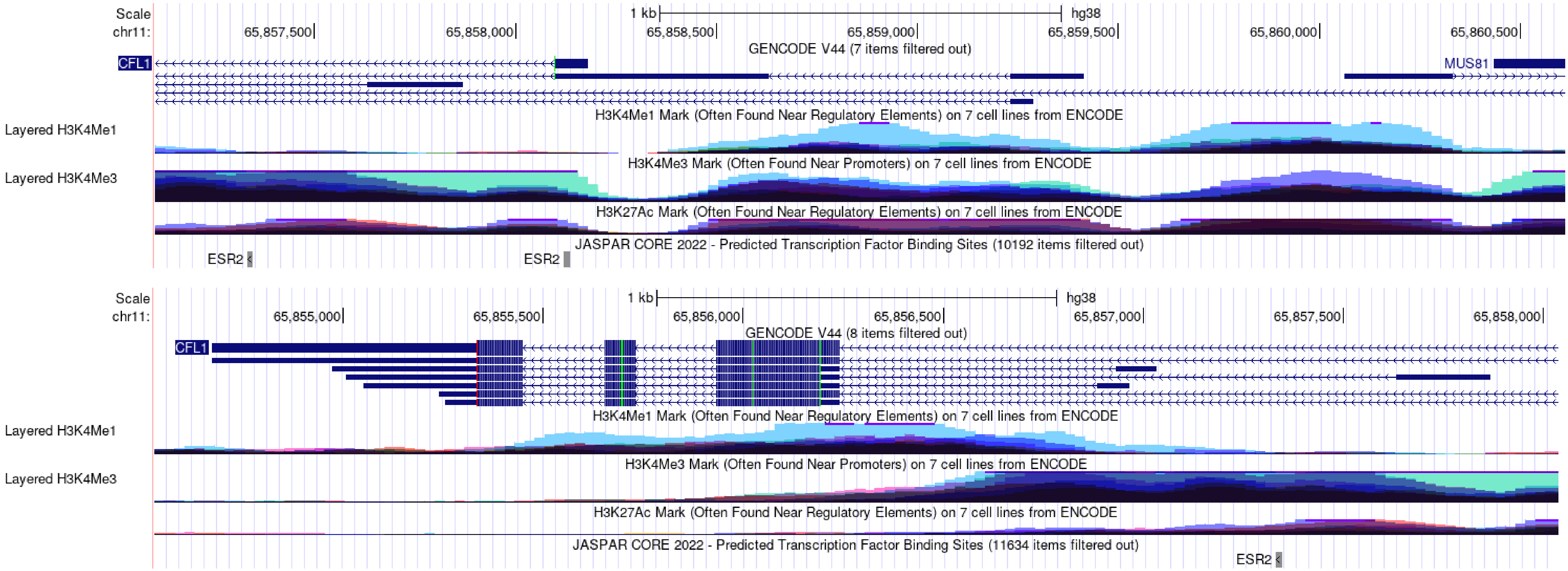

Figure S4 UCSC Genome Browser screengrab showing epigenetic marks of open chromatin and ERβ (*ESR2*) and HNF3α (*FOXA1*) binding sites in genes of interest. (a) *ACIN1*, (b) *FNBP4*, (c) *MDM4*, (d) *NDUFB3*, (e) *OCIAD2*, (f) *PLIN3*, (g) *POU2AF1*, (h) *RAC1*, (i) *SYNE2*, (j) *CFL1*, (k) *TMEM141*.

FigS4k

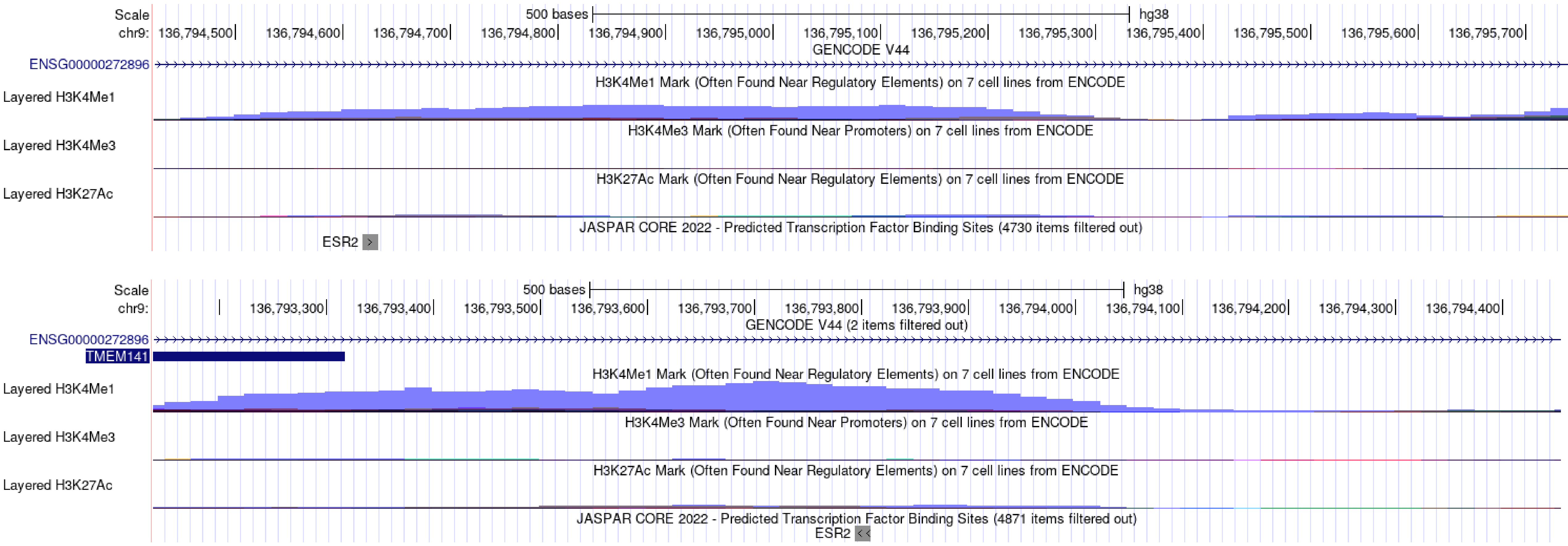

Figure S4 UCSC Genome Browser screengrab showing epigenetic marks of open chromatin and ERβ (*ESR2*) and HNF3α (*FOXA1*) binding sites in genes of interest. (a) *ACIN1*, (b) *FNBP4*, (c) *MDM4*, (d) *NDUFB3*, (e) *OCIAD2*, (f) *PLIN3*, (g) *POU2AF1*, (h) *RAC1*, (i) *SYNE2*, (j) *CFL1*, (k) *TMEM141*.

FigS5a

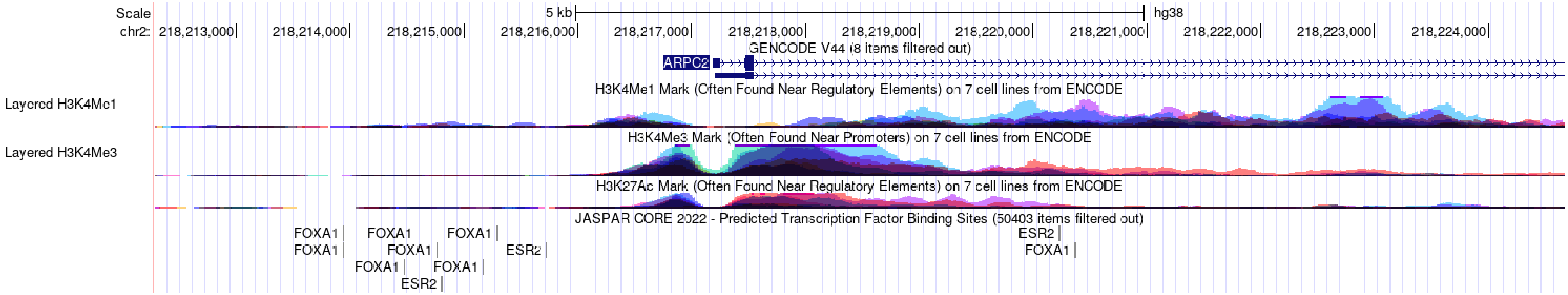

Figure S5 UCSC Genome Browser screengrab showing lack of either epigenetic marks of open chromatin with ERβ (*ESR2*) and HNF3α (*FOXA1*) binding sites in genes of interest. (a) *ARPC2*, (b) *COL4A3*, (c) *FCRL5*, (d) *LENG8*, (e) *RAP1GDS1*, (f) *VAMP1*, (g) *ZFYVE26*, (h) *FAM30A*, (i) *PPP1R3E*, (j) *TPTEP2*, (k) *ZBTB25*, (l) *CELF6*.

FigS5b

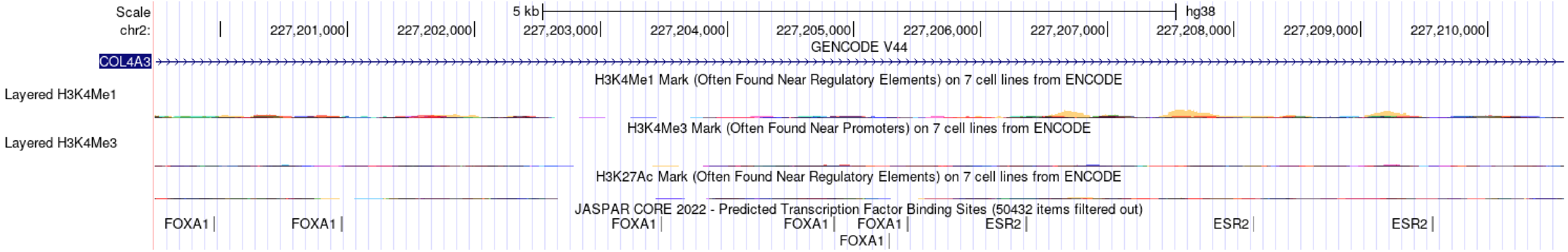

Figure S5 UCSC Genome Browser screengrab showing lack of either epigenetic marks of open chromatin with ERβ (*ESR2*) and HNF3α (*FOXA1*) binding sites in genes of interest. (a) *ARPC2*, (b) *COL4A3*, (c) *FCRL5*, (d) *LENG8*, (e) *RAP1GDS1*, (f) *VAMP1*, (g) *ZFYVE26*, (h) *FAM30A*, (i) *PPP1R3E*, (j) *TPTEP2*, (k) *ZBTB25*, (l) *CELF6*.

FigS5c

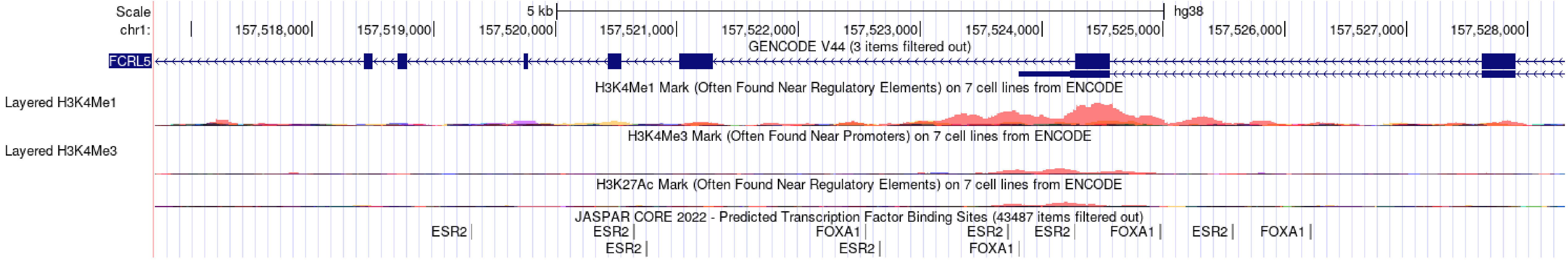

Figure S5 UCSC Genome Browser screengrab showing lack of either epigenetic marks of open chromatin with ERβ (*ESR2*) and HNF3α (*FOXA1*) binding sites in genes of interest. (a) *ARPC2*, (b) *COL4A3*, (c) *FCRL5*, (d) *LENG8*, (e) *RAP1GDS1*, (f) *VAMP1*, (g) *ZFYVE26*, (h) *FAM30A*, (i) *PPP1R3E*, (j) *TPTEP2*, (k) *ZBTB25*, (l) *CELF6*.

FigS5d

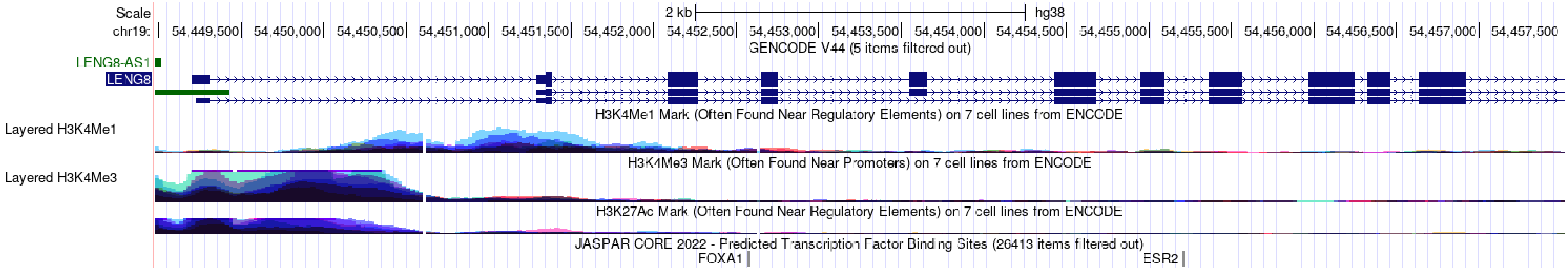

Figure S5 UCSC Genome Browser screengrab showing lack of either epigenetic marks of open chromatin with ERβ (*ESR2*) and HNF3α (*FOXA1*) binding sites in genes of interest. (a) *ARPC2*, (b) *COL4A3*, (c) *FCRL5*, (d) *LENG8*, (e) *RAP1GDS1*, (f) *VAMP1*, (g) *ZFYVE26*, (h) *FAM30A*, (i) *PPP1R3E*, (j) *TPTEP2*, (k) *ZBTB25*, (l) *CELF6*.

FigS5e

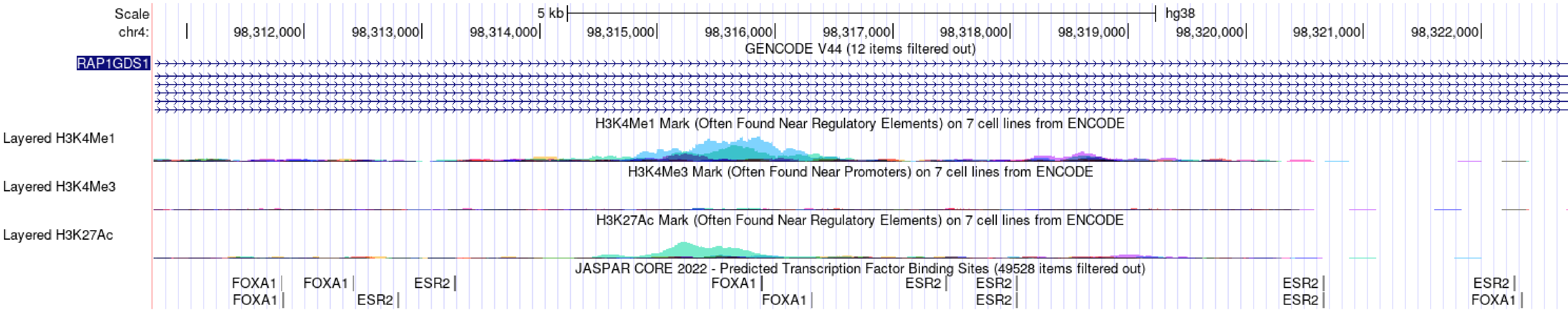

Figure S5 UCSC Genome Browser screengrab showing lack of either epigenetic marks of open chromatin with ERβ (*ESR2*) and HNF3α (*FOXA1*) binding sites in genes of interest. (a) *ARPC2*, (b) *COL4A3*, (c) *FCRL5*, (d) *LENG8*, (e) *RAP1GDS1*, (f) *VAMP1*, (g) *ZFYVE26*, (h) *FAM30A*, (i) *PPP1R3E*, (j) *TPTEP2*, (k) *ZBTB25*, (l) *CELF6*.

FigS5f

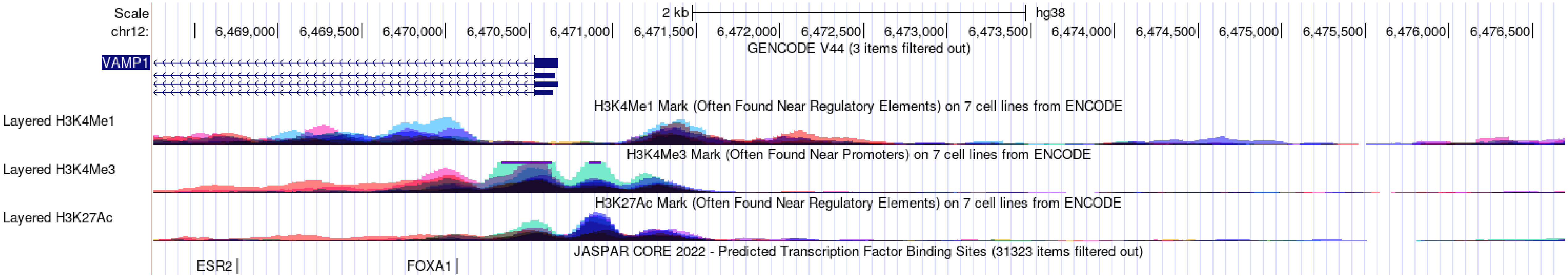

Figure S5 UCSC Genome Browser screengrab showing lack of either epigenetic marks of open chromatin with ERβ (*ESR2*) and HNF3α (*FOXA1*) binding sites in genes of interest. (a) *ARPC2*, (b) *COL4A3*, (c) *FCRL5*, (d) *LENG8*, (e) *RAP1GDS1*, (f) *VAMP1*, (g) *ZFYVE26*, (h) *FAM30A*, (i) *PPP1R3E*, (j) *TPTEP2*, (k) *ZBTB25*, (l) *CELF6*.

FigS5g

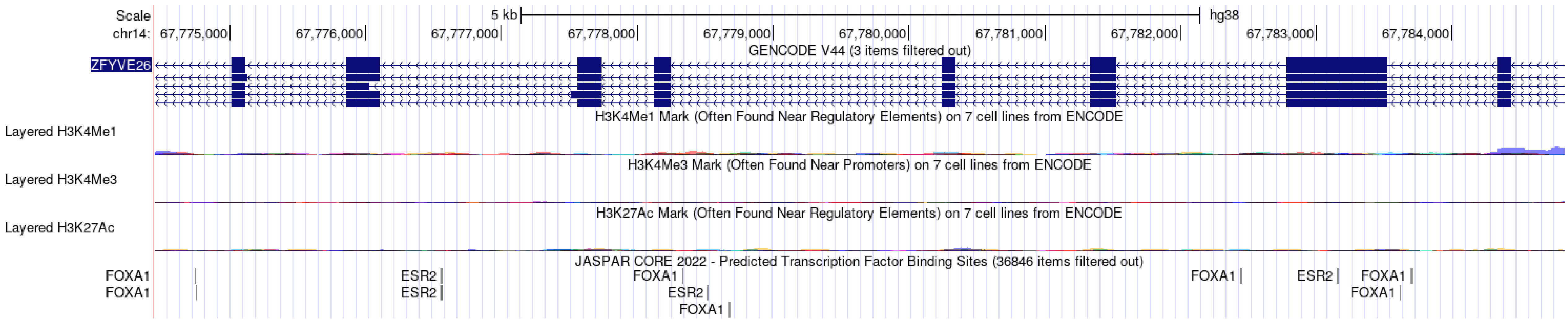

Figure S5 UCSC Genome Browser screengrab showing lack of either epigenetic marks of open chromatin with ERβ (*ESR2*) and HNF3α (*FOXA1*) binding sites in genes of interest. (a) *ARPC2*, (b) *COL4A3*, (c) *FCRL5*, (d) *LENG8*, (e) *RAP1GDS1*, (f) *VAMP1*, (g) *ZFYVE26*, (h) *FAM30A*, (i) *PPP1R3E*, (j) *TPTEP2*, (k) *ZBTB25*, (l) *CELF6*.

FigS5h

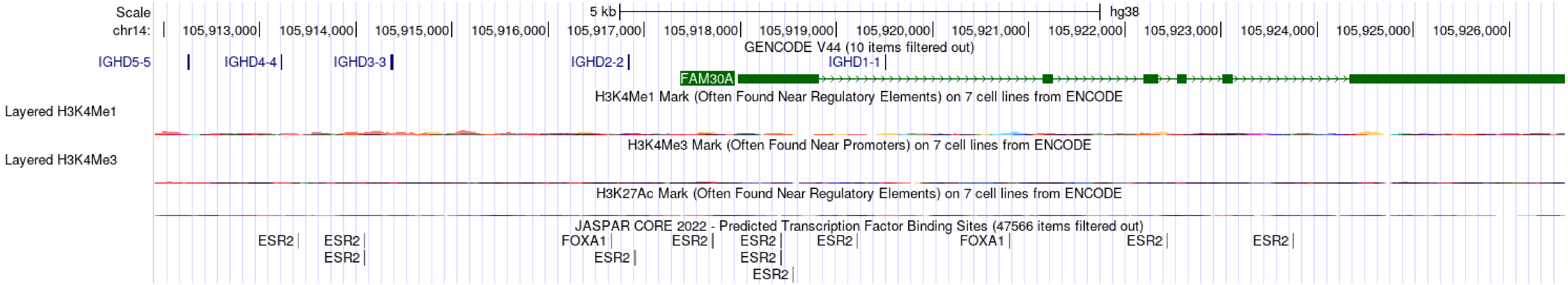

Figure S5 UCSC Genome Browser screengrab showing lack of either epigenetic marks of open chromatin with ERβ (*ESR2*) and HNF3α (*FOXA1*) binding sites in genes of interest. (a) *ARPC2*, (b) *COL4A3*, (c) *FCRL5*, (d) *LENG8*, (e) *RAP1GDS1*, (f) *VAMP1*, (g) *ZFYVE26*, (h) *FAM30A*, (i) *PPP1R3E*, (j) *TPTEP2*, (k) *ZBTB25*, (l) *CELF6*.

FigS5i

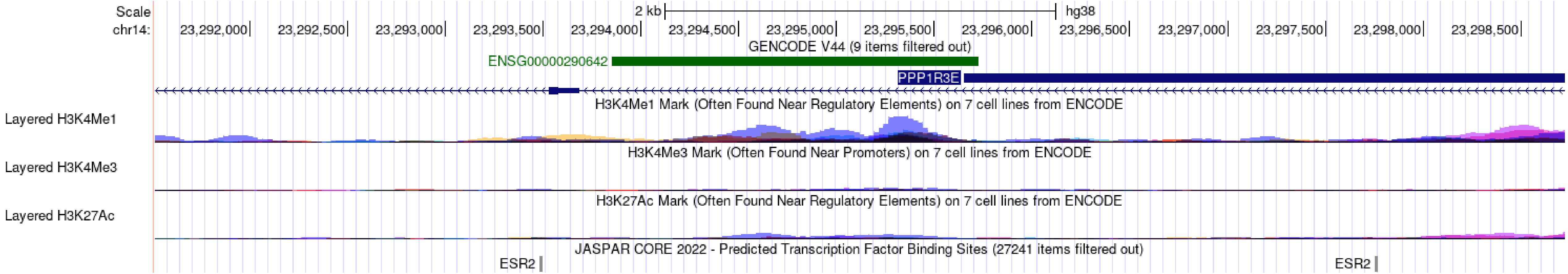

Figure S5 UCSC Genome Browser screengrab showing lack of either epigenetic marks of open chromatin with ERβ (*ESR2*) and HNF3α (*FOXA1*) binding sites in genes of interest. (a) *ARPC2*, (b) *COL4A3*, (c) *FCRL5*, (d) *LENG8*, (e) *RAP1GDS1*, (f) *VAMP1*, (g) *ZFYVE26*, (h) *FAM30A*, (i) *PPP1R3E*, (j) *TPTEP2*, (k) *ZBTB25*, (l) *CELF6*.

FigS5j

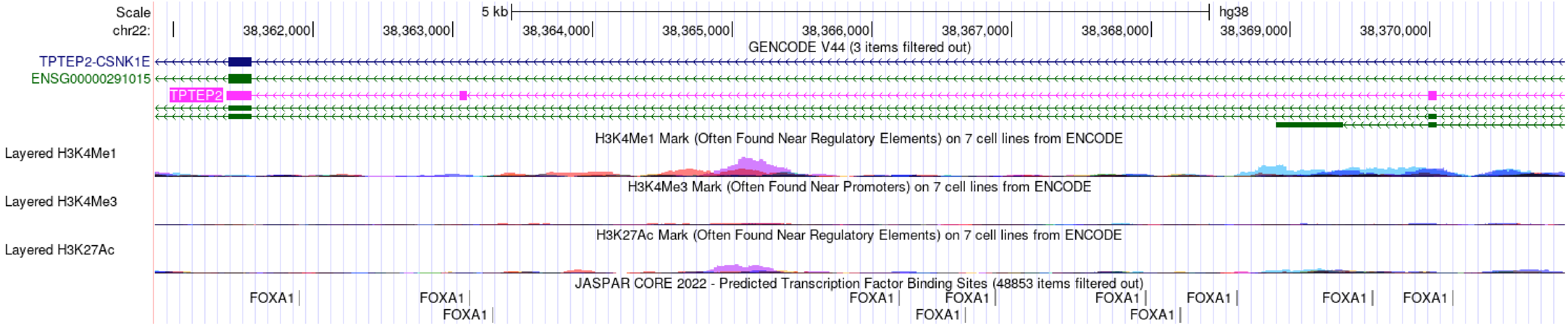

Figure S5 UCSC Genome Browser screengrab showing lack of either epigenetic marks of open chromatin with ERβ (*ESR2*) and HNF3α (*FOXA1*) binding sites in genes of interest. (a) *ARPC2*, (b) *COL4A3*, (c) *FCRL5*, (d) *LENG8*, (e) *RAP1GDS1*, (f) *VAMP1*, (g) *ZFYVE26*, (h) *FAM30A*, (i) *PPP1R3E*, (j) *TPTEP2*, (k) *ZBTB25*, (l) *CELF6*.

FigS5k

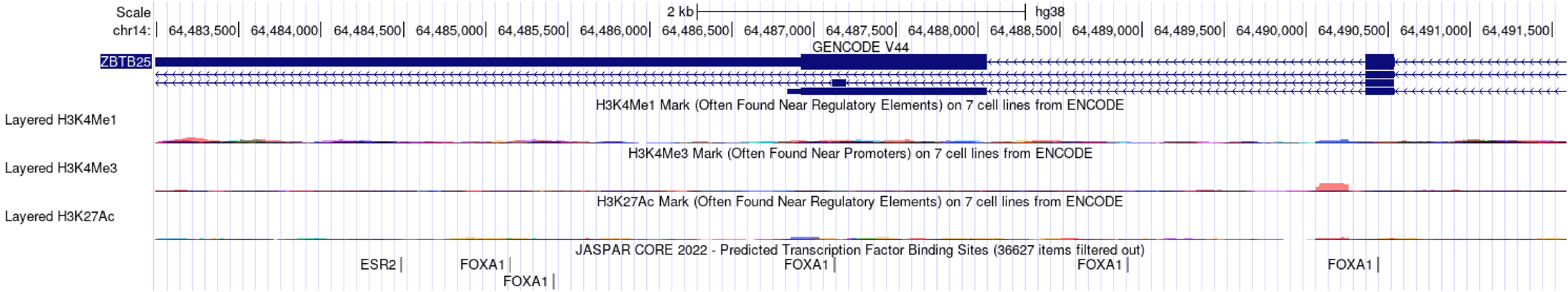

Figure S5 UCSC Genome Browser screengrab showing lack of either epigenetic marks of open chromatin with ERβ (*ESR2*) and HNF3α (*FOXA1*) binding sites in genes of interest. (a) *ARPC2*, (b) *COL4A3*, (c) *FCRL5*, (d) *LENG8*, (e) *RAP1GDS1*, (f) *VAMP1*, (g) *ZFYVE26*, (h) *FAM30A*, (i) *PPP1R3E*, (j) *TPTEP2*, (k) *ZBTB25*, (l) *CELF6*.

FigS5l

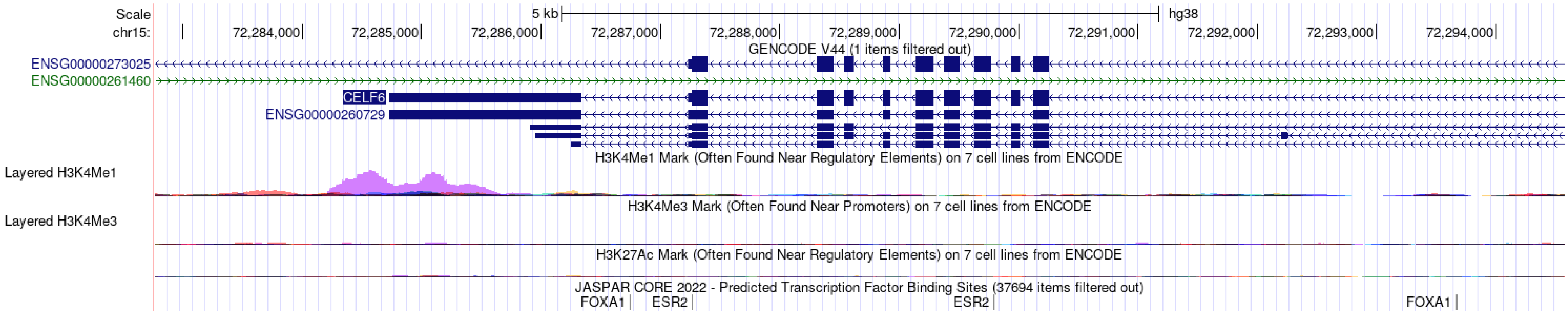

Figure S5 UCSC Genome Browser screengrab showing lack of either epigenetic marks of open chromatin with ERβ (*ESR2*) and HNF3α (*FOXA1*) binding sites in genes of interest. (a) *ARPC2*, (b) *COL4A3*, (c) *FCRL5*, (d) *LENG8*, (e) *RAP1GDS1*, (f) *VAMP1*, (g) *ZFYVE26*, (h) *FAM30A*, (i) *PPP1R3E*, (j) *TPTEP2*, (k) *ZBTB25*, (l) *CELF6*.

FigS6a

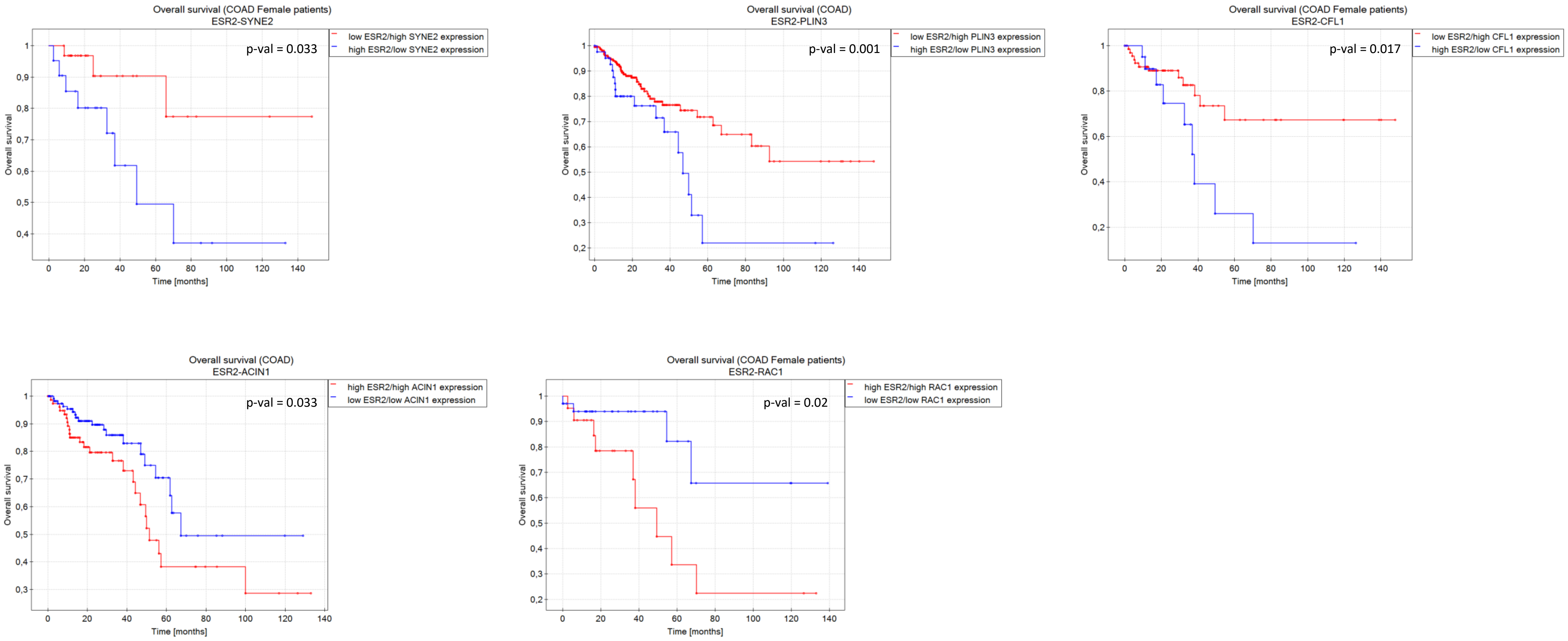

Figure S6 Kapplan-Meier plots of TCGA tumor types patients (with or without sex as a factor) with co-expression of *ESR2* and selected target genes as a factor in terms of OS. (a) COAD, (b) PRAD, (c) LUSC, (d) MESO, (e) SARC, (f) TGCT, (g) THCA, (h) UCEC. p-value <0.05, FDR <0.05

FigS6b

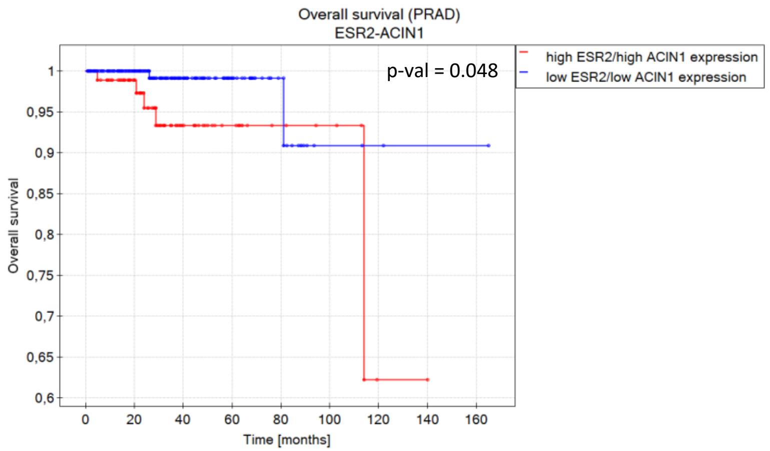

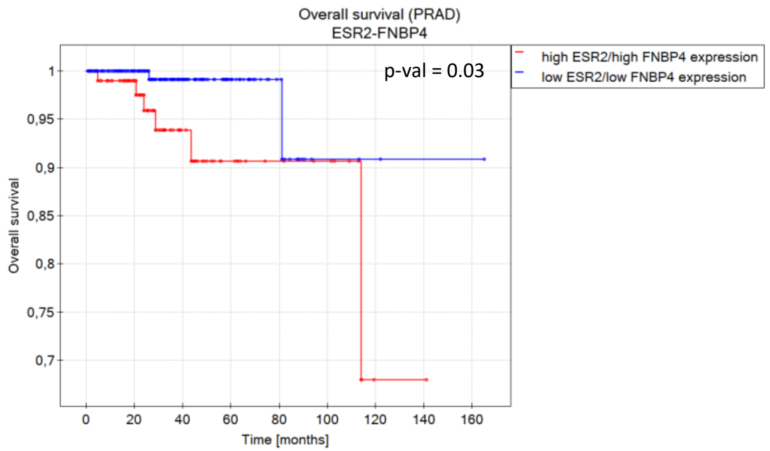

Figure S6 Kapplan-Meier plots of TCGA tumor types patients (with or without sex as a factor) with co-expression of *ESR2* and selected target genes as a factor in terms of OS. (a) COAD, (b) PRAD, (c) LUSC, (d) MESO, (e) SARC, (f) TGCT, (g) THCA, (h) UCEC. p-value <0.05, FDR <0.05

FigS6c

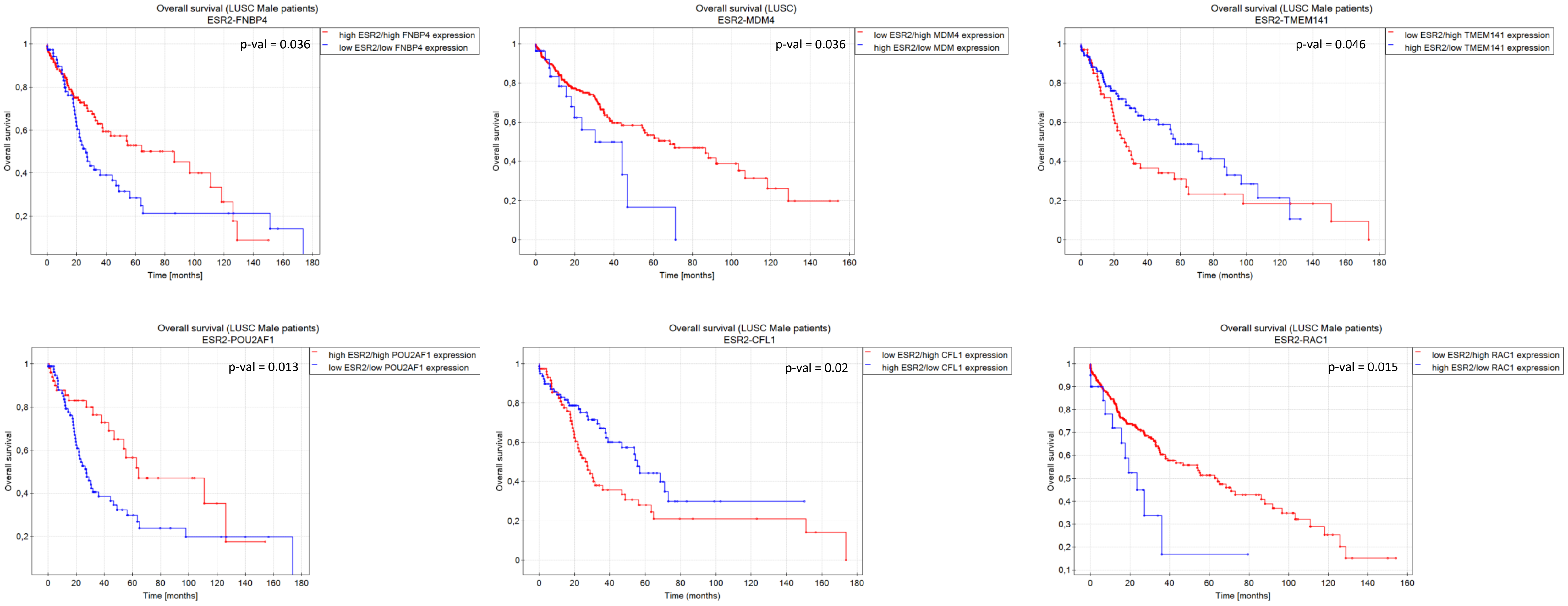

Figure S6 Kapplan-Meier plots of TCGA tumor types patients (with or without sex as a factor) with co-expression of *ESR2* and selected target genes as a factor in terms of OS. (a) COAD, (b) PRAD, (c) LUSC, (d) MESO, (e) SARC, (f) TGCT, (g) THCA, (h) UCEC. p-value <0.05, FDR <0.05

FigS6d

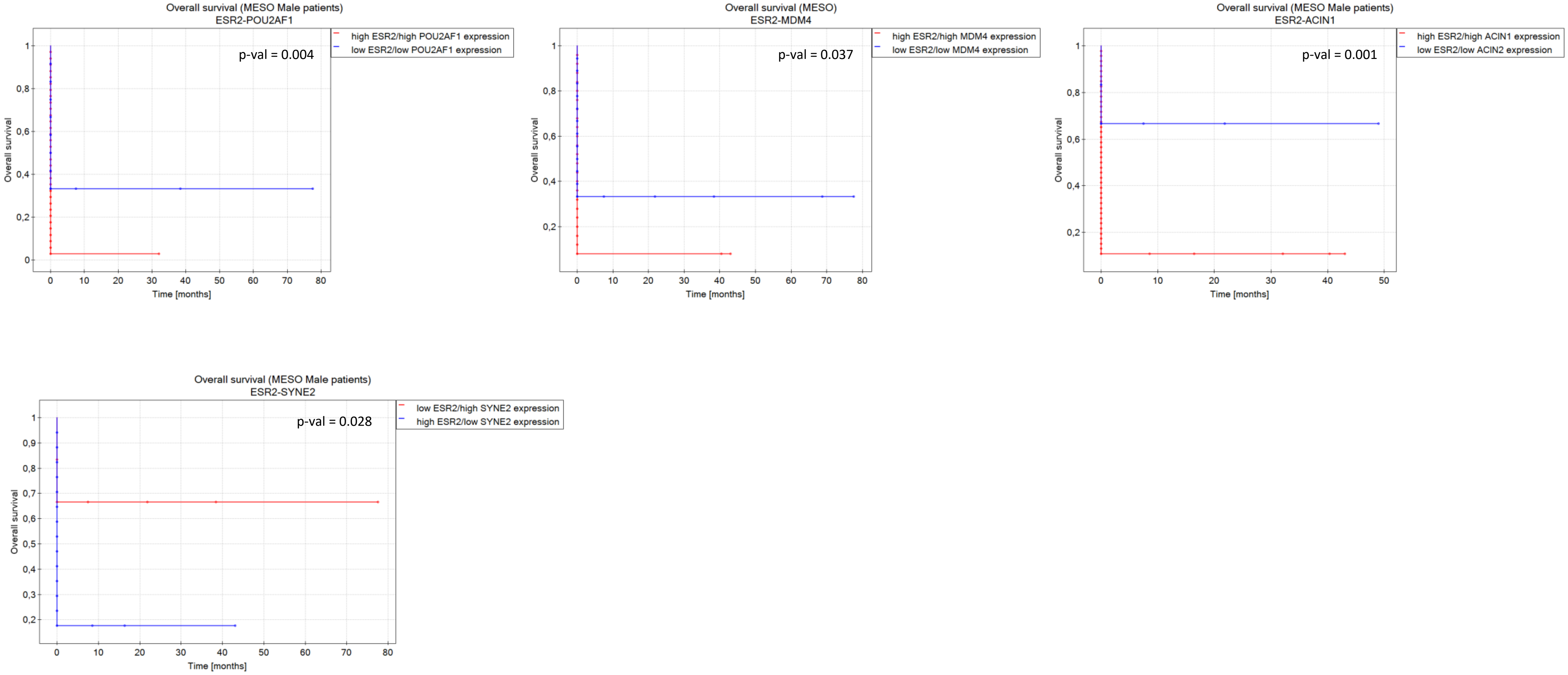

Figure S6 Kapplan-Meier plots of TCGA tumor types patients (with or without sex as a factor) with co-expression of *ESR2* and selected target genes as a factor in terms of OS. (a) COAD, (b) PRAD, (c) LUSC, (d) MESO, (e) SARC, (f) TGCT, (g) THCA, (h) UCEC. p-value <0.05, FDR <0.05

FigS6e

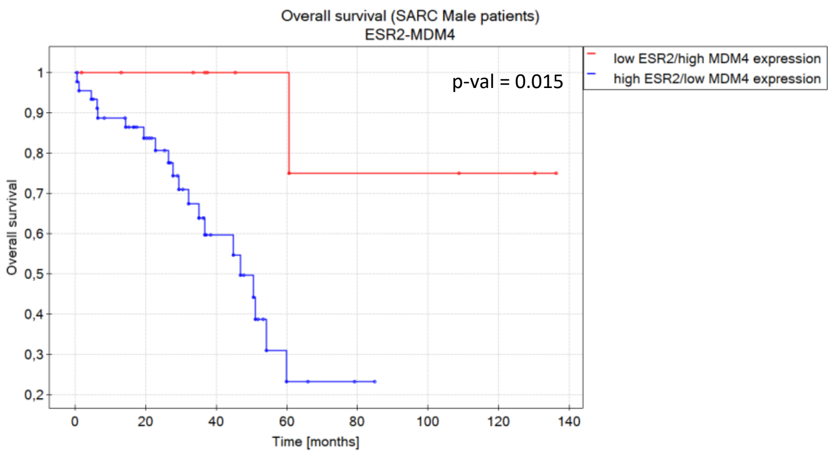

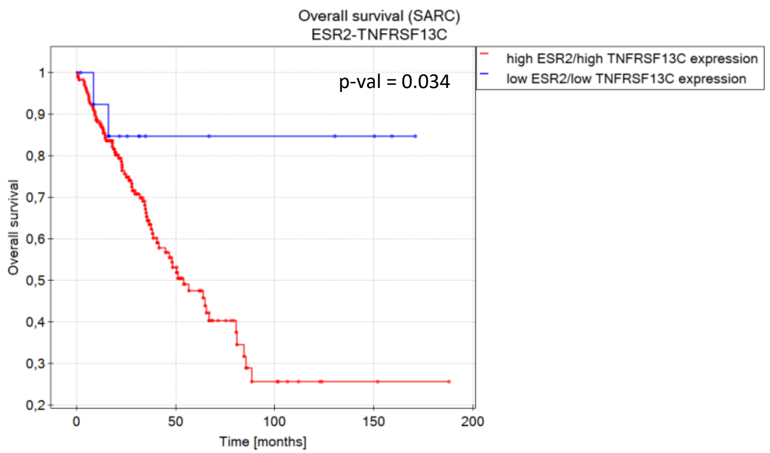

Figure S6 Kapplan-Meier plots of TCGA tumor types patients (with or without sex as a factor) with co-expression of *ESR2* and selected target genes as a factor in terms of OS. (a) COAD, (b) PRAD, (c) LUSC, (d) MESO, (e) SARC, (f) TGCT, (g) THCA, (h) UCEC. p-value <0.05, FDR <0.05

FigS6f

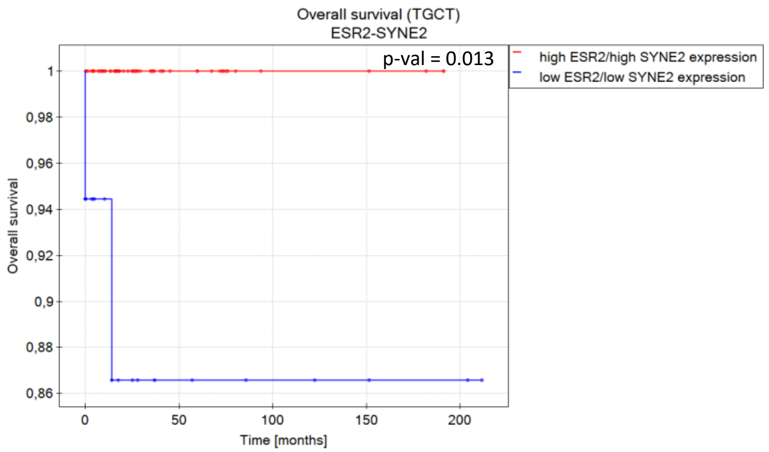

Figure S6 Kapplan-Meier plots of TCGA tumor types patients (with or without sex as a factor) with co-expression of *ESR2* and selected target genes as a factor in terms of OS. (a) COAD, (b) PRAD, (c) LUSC, (d) MESO, (e) SARC, (f) TGCT, (g) THCA, (h) UCEC. p-value <0.05, FDR <0.05

FigS6g

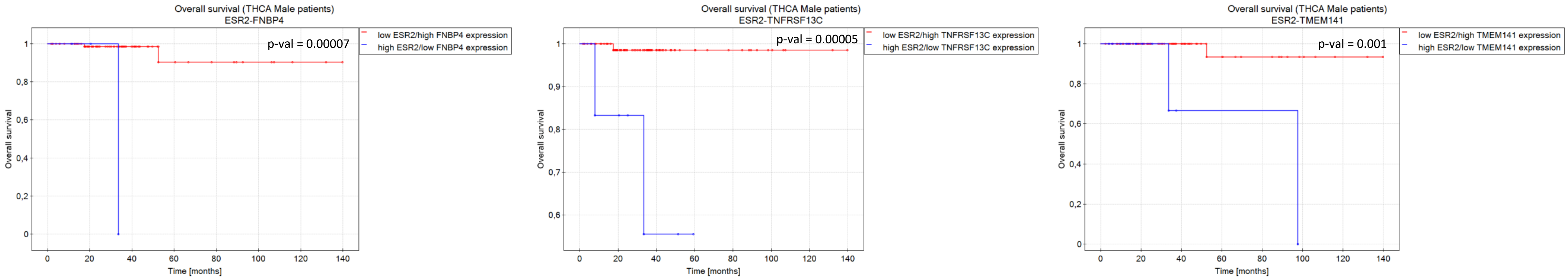

Figure S6 Kapplan-Meier plots of TCGA tumor types patients (with or without sex as a factor) with co-expression of *ESR2* and selected target genes as a factor in terms of OS. (a) COAD, (b) PRAD, (c) LUSC, (d) MESO, (e) SARC, (f) TGCT, (g) THCA, (h) UCEC. p-value <0.05, FDR <0.05

FigS6h

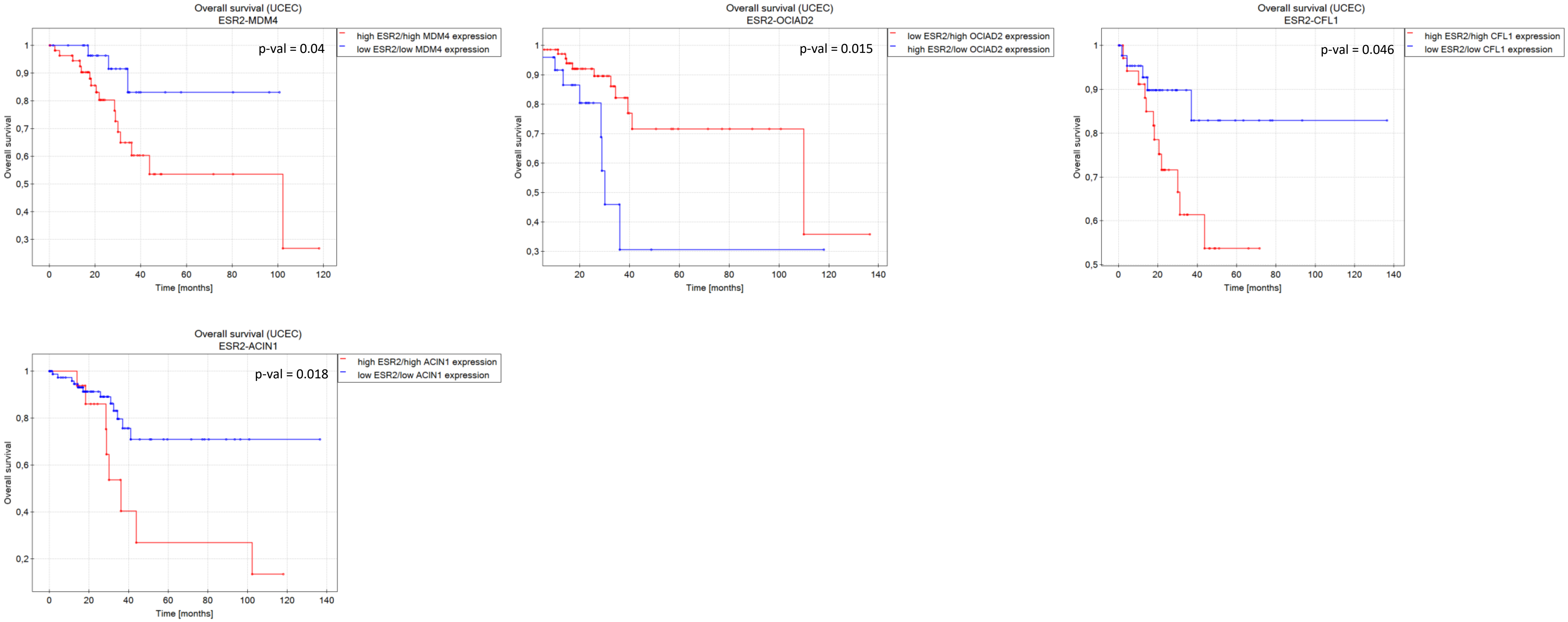

Figure S6 Kapplan-Meier plots of TCGA tumor types patients (with or without sex as a factor) with co-expression of *ESR2* and selected target genes as a factor in terms of OS. (a) COAD, (b) PRAD, (c) LUSC, (d) MESO, (e) SARC, (f) TGCT, (g) THCA, (h) UCEC. p-value <0.05, FDR <0.05

FigS7a

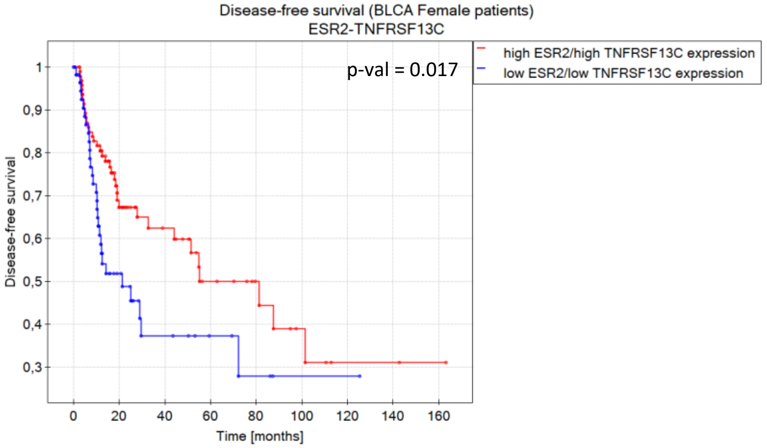

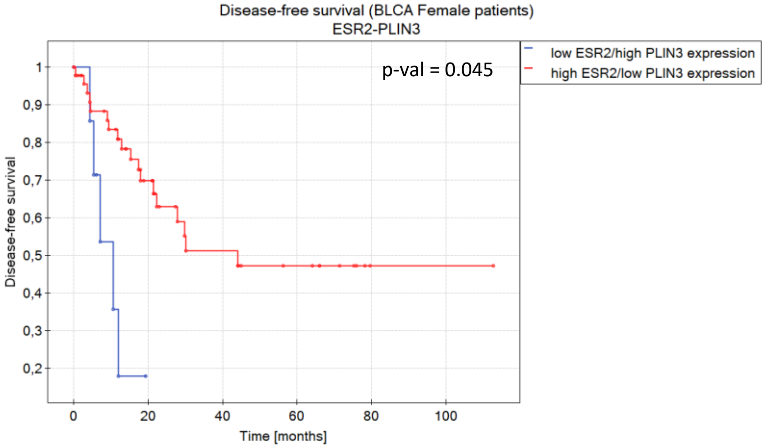

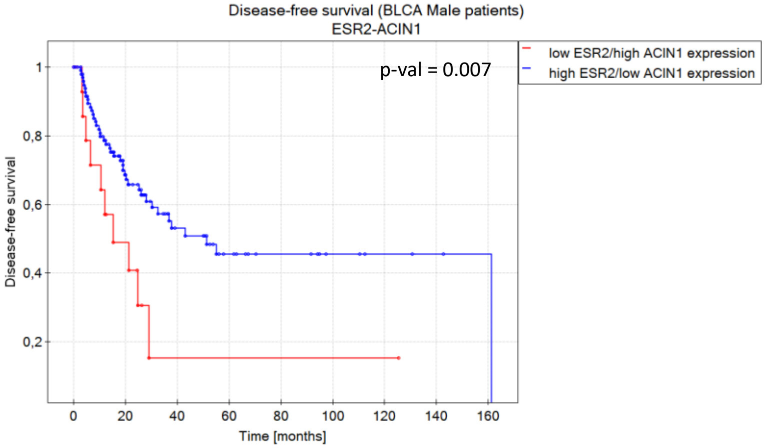

Figure S7 Kapplan-Meier plots of TCGA tumor types patients (with or without sex as a factor) with co-expression of *ESR2* and selected target genes as a factor in terms of DFS. (a) BLCA, (b) BRCA, (c) CESC, (d) HNSC, (e) CHOL, (f) COAD, (g) LUAD, (h) LUSC, (i) PAAD, (j) SARC, (k) SKCM, (l) THYM, (m) THCA. p-value <0.05, FDR <0.05

FigS7b

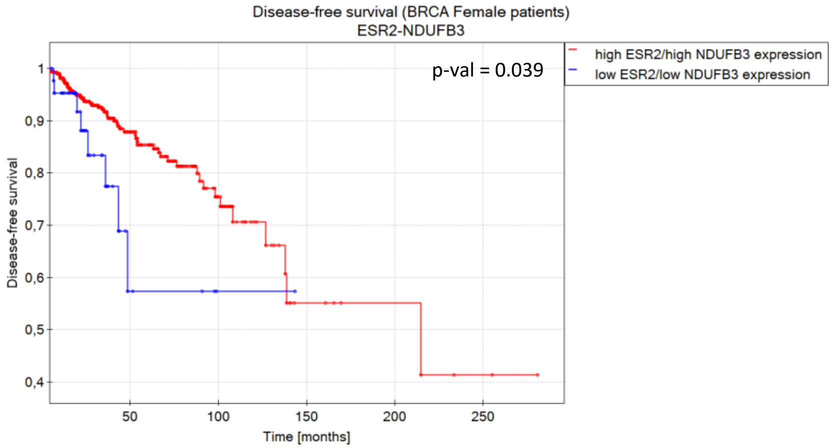

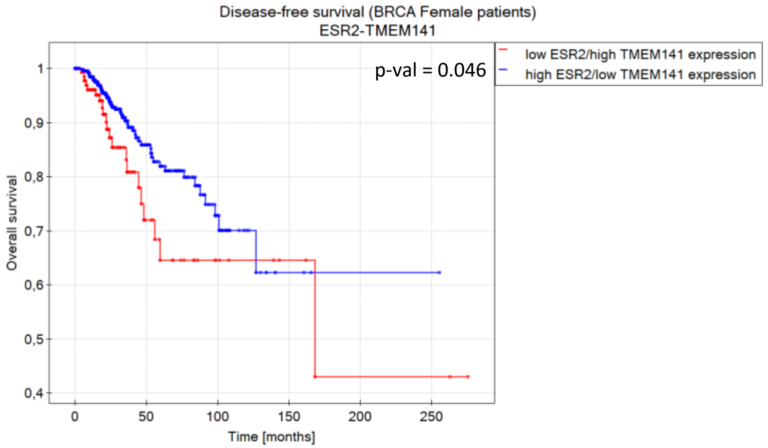

Figure S7 Kapplan-Meier plots of TCGA tumor types patients (with or without sex as a factor) with co-expression of *ESR2* and selected target genes as a factor in terms of DFS. (a) BLCA, (b) BRCA, (c) CESC, (d) HNSC, (e) CHOL, (f) COAD, (g) LUAD, (h) LUSC, (i) PAAD, (j) SARC, (k) SKCM, (l) THYM, (m) THCA. p-value <0.05, FDR <0.05

FigS7c

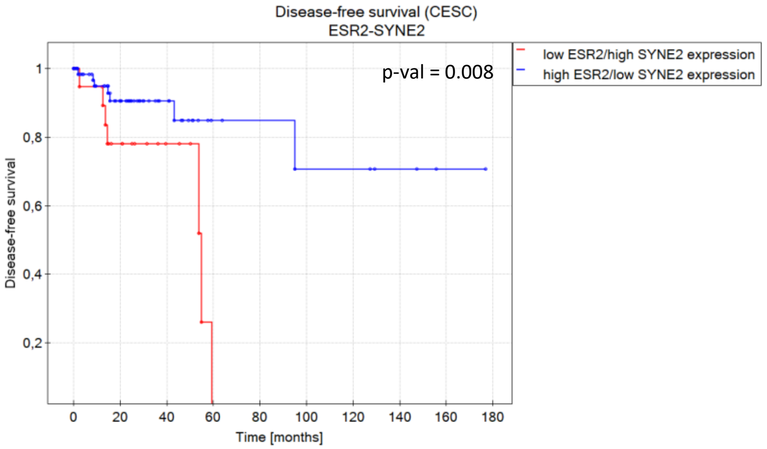

Figure S7 Kapplan-Meier plots of TCGA tumor types patients (with or without sex as a factor) with co-expression of *ESR2* and selected target genes as a factor in terms of DFS. (a) BLCA, (b) BRCA, (c) CESC, (d) HNSC, (e) CHOL, (f) COAD, (g) LUAD, (h) LUSC, (i) PAAD, (j) SARC, (k) SKCM, (l) THYM, (m) THCA. p-value <0.05, FDR <0.05

FigS7d

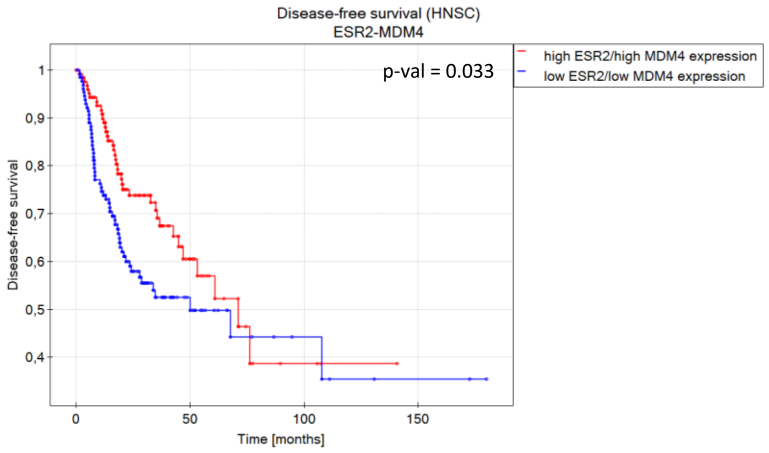

Figure S7 Kapplan-Meier plots of TCGA tumor types patients (with or without sex as a factor) with co-expression of *ESR2* and selected target genes as a factor in terms of DFS. (a) BLCA, (b) BRCA, (c) CESC, (d) HNSC, (e) CHOL, (f) COAD, (g) LUAD, (h) LUSC, (i) PAAD, (j) SARC, (k) SKCM, (l) THYM, (m) THCA. p-value <0.05, FDR <0.05

FigS7e

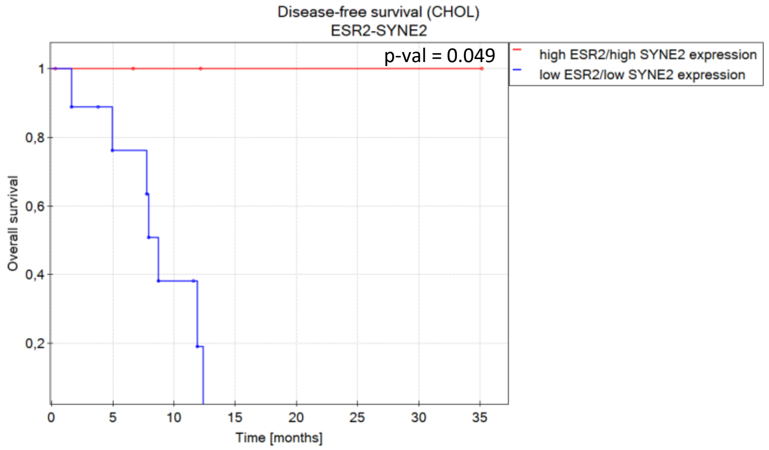

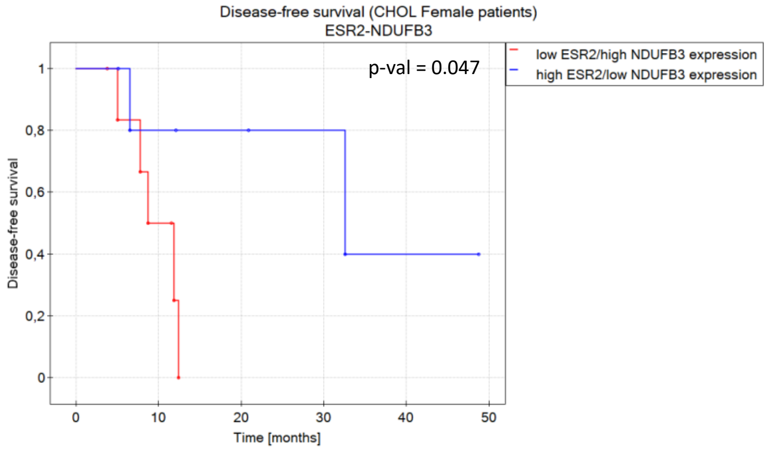

Figure S7 Kapplan-Meier plots of TCGA tumor types patients (with or without sex as a factor) with co-expression of *ESR2* and selected target genes as a factor in terms of DFS. (a) BLCA, (b) BRCA, (c) CESC, (d) HNSC, (e) CHOL, (f) COAD, (g) LUAD, (h) LUSC, (i) PAAD, (j) SARC, (k) SKCM, (l) THYM, (m) THCA. p-value <0.05, FDR <0.05

FigS7f

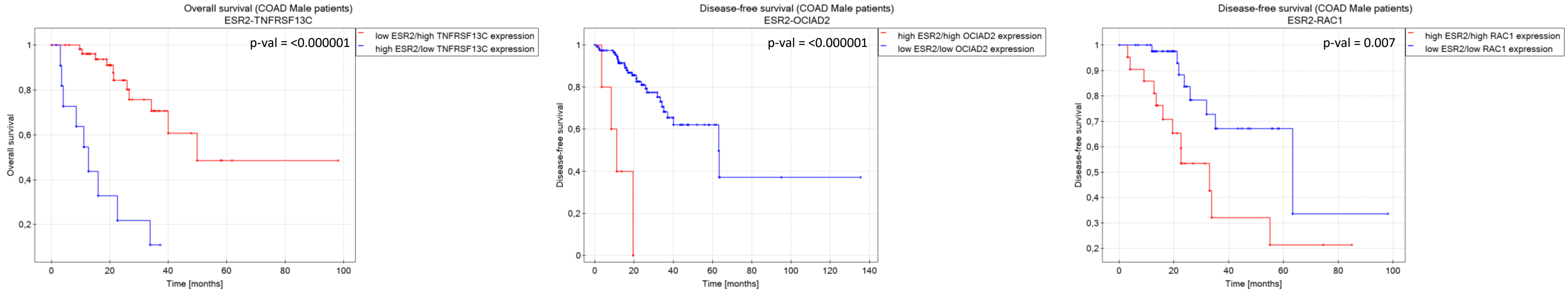

Figure S7 Kapplan-Meier plots of TCGA tumor types patients (with or without sex as a factor) with co-expression of *ESR2* and selected target genes as a factor in terms of DFS. (a) BLCA, (b) BRCA, (c) CESC, (d) HNSC, (e) CHOL, (f) COAD, (g) LUAD, (h) LUSC, (i) PAAD, (j) SARC, (k) SKCM, (l) THYM, (m) THCA. p-value <0.05, FDR <0.05

FigS7g

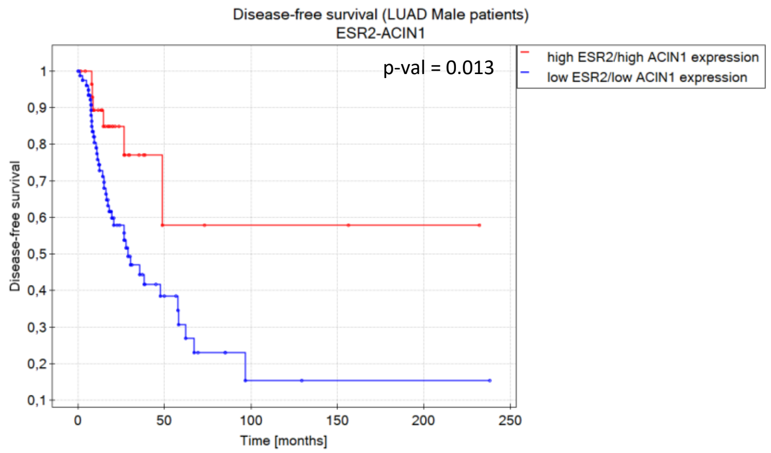

Figure S7 Kapplan-Meier plots of TCGA tumor types patients (with or without sex as a factor) with co-expression of *ESR2* and selected target genes as a factor in terms of DFS. (a) BLCA, (b) BRCA, (c) CESC, (d) HNSC, (e) CHOL, (f) COAD, (g) LUAD, (h) LUSC, (i) PAAD, (j) SARC, (k) SKCM, (l) THYM, (m) THCA. p-value <0.05, FDR <0.05

FigS7h

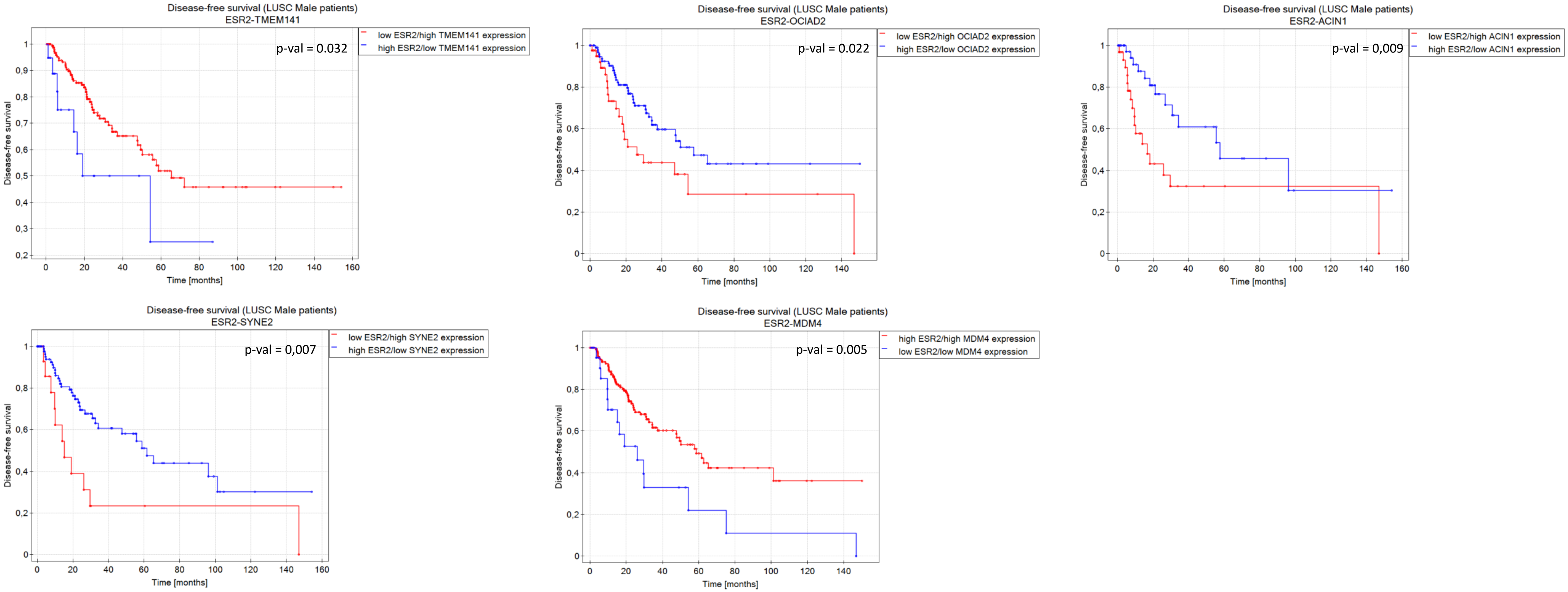

Figure S7 Kapplan-Meier plots of TCGA tumor types patients (with or without sex as a factor) with co-expression of *ESR2* and selected target genes as a factor in terms of DFS. (a) BLCA, (b) BRCA, (c) CESC, (d) HNSC, (e) CHOL, (f) COAD, (g) LUAD, (h) LUSC, (i) PAAD, (j) SARC, (k) SKCM, (l) THYM, (m) THCA. p-value <0.05, FDR <0.05

FigS7i

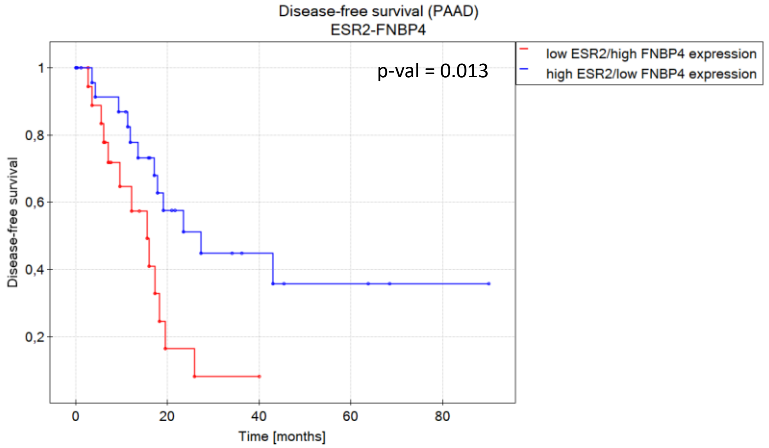

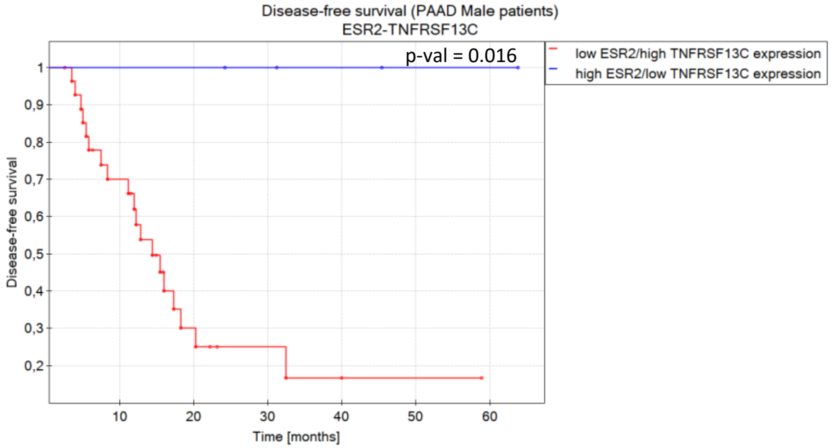

Figure S7 Kapplan-Meier plots of TCGA tumor types patients (with or without sex as a factor) with co-expression of *ESR2* and selected target genes as a factor in terms of DFS. (a) BLCA, (b) BRCA, (c) CESC, (d) HNSC, (e) CHOL, (f) COAD, (g) LUAD, (h) LUSC, (i) PAAD, (j) SARC, (k) SKCM, (l) THYM, (m) THCA. p-value <0.05, FDR <0.05

FigS7j

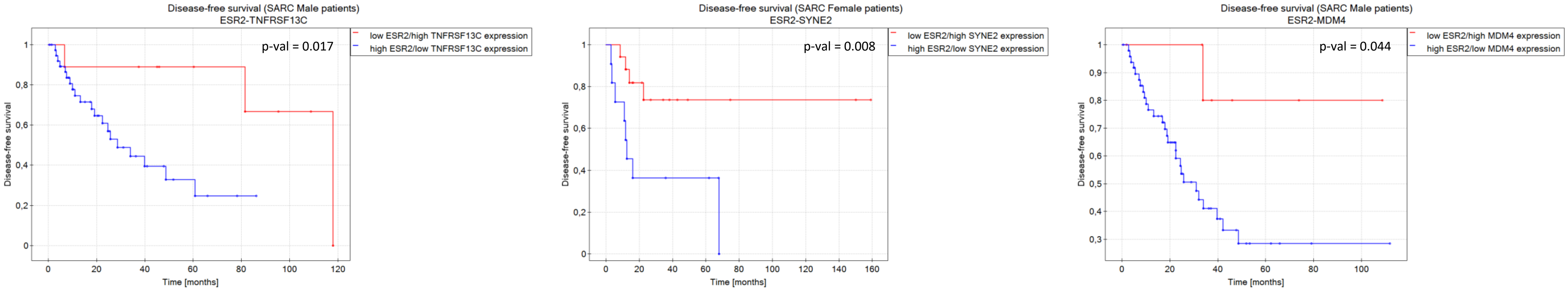

Figure S7 Kapplan-Meier plots of TCGA tumor types patients (with or without sex as a factor) with co-expression of *ESR2* and selected target genes as a factor in terms of DFS. (a) BLCA, (b) BRCA, (c) CESC, (d) HNSC, (e) CHOL, (f) COAD, (g) LUAD, (h) LUSC, (i) PAAD, (j) SARC, (k) SKCM, (l) THYM, (m) THCA. p-value <0.05, FDR <0.05

FigS7k

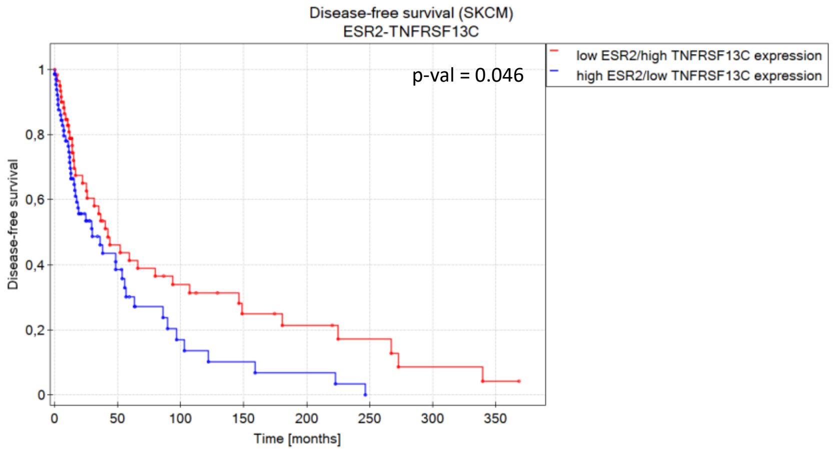

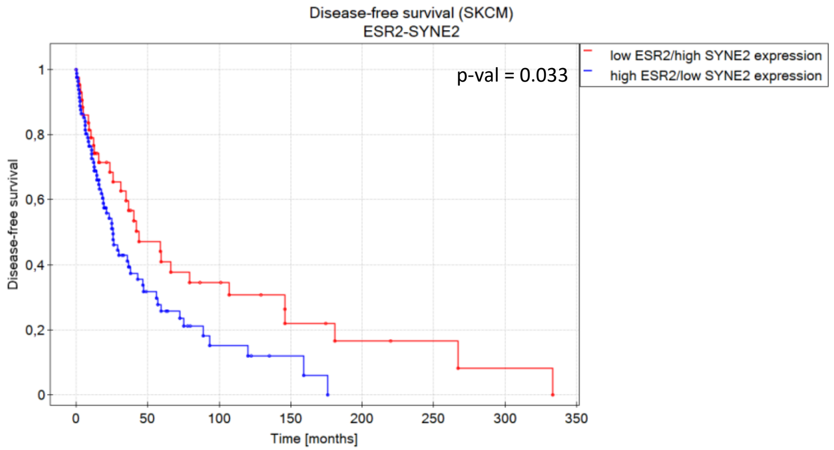

Figure S7 Kapplan-Meier plots of TCGA tumor types patients (with or without sex as a factor) with co-expression of *ESR2* and selected target genes as a factor in terms of DFS. (a) BLCA, (b) BRCA, (c) CESC, (d) HNSC, (e) CHOL, (f) COAD, (g) LUAD, (h) LUSC, (i) PAAD, (j) SARC, (k) SKCM, (l) THYM, (m) THCA. p-value <0.05, FDR <0.05

FigS7l

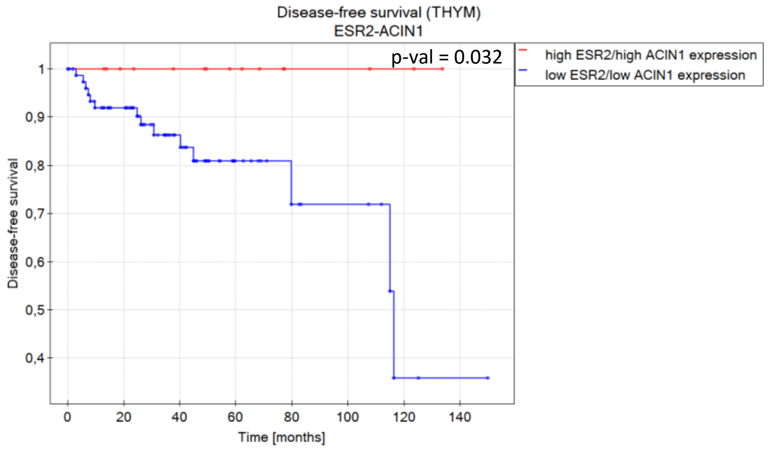

Figure S7 Kapplan-Meier plots of TCGA tumor types patients (with or without sex as a factor) with co-expression of *ESR2* and selected target genes as a factor in terms of DFS. (a) BLCA, (b) BRCA, (c) CESC, (d) HNSC, (e) CHOL, (f) COAD, (g) LUAD, (h) LUSC, (i) PAAD, (j) SARC, (k) SKCM, (l) THYM, (m) THCA. p-value <0.05, FDR <0.05

FigS7m

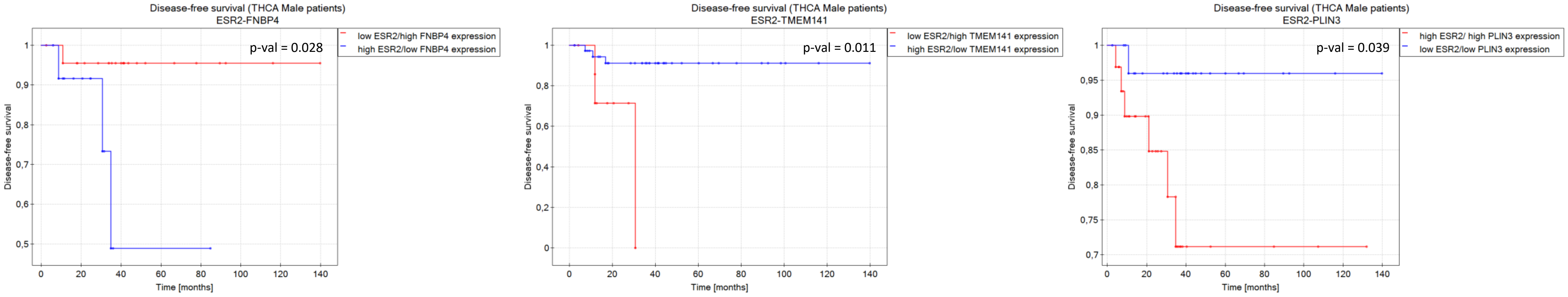

Figure S7 Kapplan-Meier plots of TCGA tumor types patients (with or without sex as a factor) with co-expression of *ESR2* and selected target genes as a factor in terms of DFS. (a) BLCA, (b) BRCA, (c) CESC, (d) HNSC, (e) CHOL, (f) COAD, (g) LUAD, (h) LUSC, (i) PAAD, (j) SARC, (k) SKCM, (l) THYM, (m) THCA. p-value <0.05, FDR <0.05
